# Dynamic protrusions mediate crawling motility in Asgard archaea

**DOI:** 10.1038/s41586-026-11063-9

**Published:** 2026-09-30

**Authors:** Philipp Radler, Tobias Viehboeck, Zhen-Hao Luo, Nevena Maslać, Katharina Schmidt, Robert Hauschild, Masaru K. Nobu, Silvia Bulgheresi, Theresia E. B. Stradal, Klemens Rottner, Hiroyuki Imachi, Michael Sixt, Christa Schleper

**Affiliations:** 1 Department of Functional and Evolutionary Ecology, Archaea Biology and Ecogenomics Unit, University of Vienna, Vienna, Austria; 2 Department of Cell Biology, Helmholtz Centre for Infection Research, Braunschweig, Germany; 3 Institute of Science and Technology Austria, Klosterneuburg, Austria; 4 Institute for Extra-cutting-edge Science and Technology Avant-garde Research of Life (X-star), Japan Agency for Marine-Earth Science and Technology (JAMSTEC), Yokosuka, Japan; 5 Division of Molecular Cell Biology, Technische Universität Braunschweig, Germany; Molecular Cell Biology Group, Helmholtz Centre for Infection Research, Braunschweig, Germany

**Keywords:** Cellular microbiology, Amoeboid migration

## Abstract

Crawling motility is a hallmark of eukaryotic cells and requires a dynamic actin cytoskeleton, regulated adhesion and spatially organized signalling pathways^1,2^. Asgard archaea, which are considered the closest known prokaryotic relatives of eukaryotes, potentially encode these functions within their large set of ‘eukaryotic signature proteins’^3–6^. The few cultivated members show a complex cell morphology, consisting of a central cell body from which several protrusions extend, filled with an actin-based cytoskeleton^7,8^. Here, live-cell microscopy of two organisms of the Lokiarchaea and Hodarchaea lineages^7,9^ showed that they dynamically and greatly change their cell shape on a minute time scale and grow and retract their extensive protrusions with a speed of 1.5–4.8 µm min^−1^, respectively. After adhering to a glass surface, cells use their protrusions to undergo active crawling motion. In the presence of selected actin inhibitors, however, the observed dynamics were arrested, suggesting a central role of actin in these processes. The observed cellular plasticity and motility are unique features among prokaryotes and might have been crucial for the emergence of the first eukaryotic cells that are thought to have formed through the association of a member of the Asgard archaea and an α-proteobacterium, the ancestor of mitochondria.

## Abstract (web summary)

Anoxic live-cell imaging shows actin-dependent crawling motility and dynamic protrusions in Asgard archaea, providing insights into cellular innovations that may have contributed to eukaryotic evolution.

## Main

Since their discovery a decade ago, Asgard archaea (now formally classified as the phylum Promethearchaeota or Asgardarchaeota^5,8^) have inspired new models for eukaryogenesis^10–14^. Their genomes encode numerous so-called eukaryotic signature proteins (ESPs), including, for example, ESCRT and ubiquitination machineries, an actin-based cytoskeleton and associated proteins, as well as many small GTPases that were never found to this extent in other prokaryotes^3,5,15^. A recent structure-based analysis showed another plethora of ESPs in metagenome-assembled genomes^16^. Members of Promethearchaeota are now regarded as the closest prokaryotic relatives of eukaryotes and possibly as their direct ancestors about 2 billion years ago (refs. ^6,9,17^). Although current models of eukaryogenesis differ in their mechanistic details, most of them agree that eukaryotes emerged from a symbiosis or merger between an archaeon of the Promethearchaeota lineage and at least one α-Proteobacterium (or related), which evolved into mitochondria^13,18,19^. The few so-far cultivated cells of the Asgard archaea^7,9,10,20–22^ have shown a distinctive cell morphology: a central cell body from which protrusions of varying number and length extend. Furthermore, the outer membrane was not covered by a cell wall but found to be decorated with proteins, potentially used for establishing cell–cell contacts or formation of cell–substrate adhesions. The cell bodies and protrusions are filled with a cytoskeleton formed by the conserved actin homologue of Asgard archaea, referred to as Lokiactin, which is one of the most highly expressed proteins in *Candidatus* Lokiarchaeum ossiferum (L. ossiferum)^7^ and is, based on its sequence, closely related to eukaryotic actins^7,23,24^. Biochemical studies have demonstrated that profilin and gelsolins from Asgard archaea can bind and modulate eukaryotic actin, further demonstrating the close functional and evolutionary relationship of these proteins^25–27^. By analogy to eukaryotic pseudopodia, dynamic regulation of Lokiactin filaments could enable protrusion growth and retraction, thereby contributing to morphological plasticity and motility. To directly study if these processes occur in Asgard archaea, we established anaerobic live-cell imaging and demonstrated that both L. ossiferum and *Ca*. Margulisarchaeum peptidophilum (M. peptidophilum) undergo striking morphological rearrangements within minutes. Furthermore, both Asgard archaea strains exhibit crawling motility that involves extensive dynamics of their protrusions and is sensitive to actin inhibitors.

## Highly dynamic shapes of L. ossiferum

**Fig. 1:**
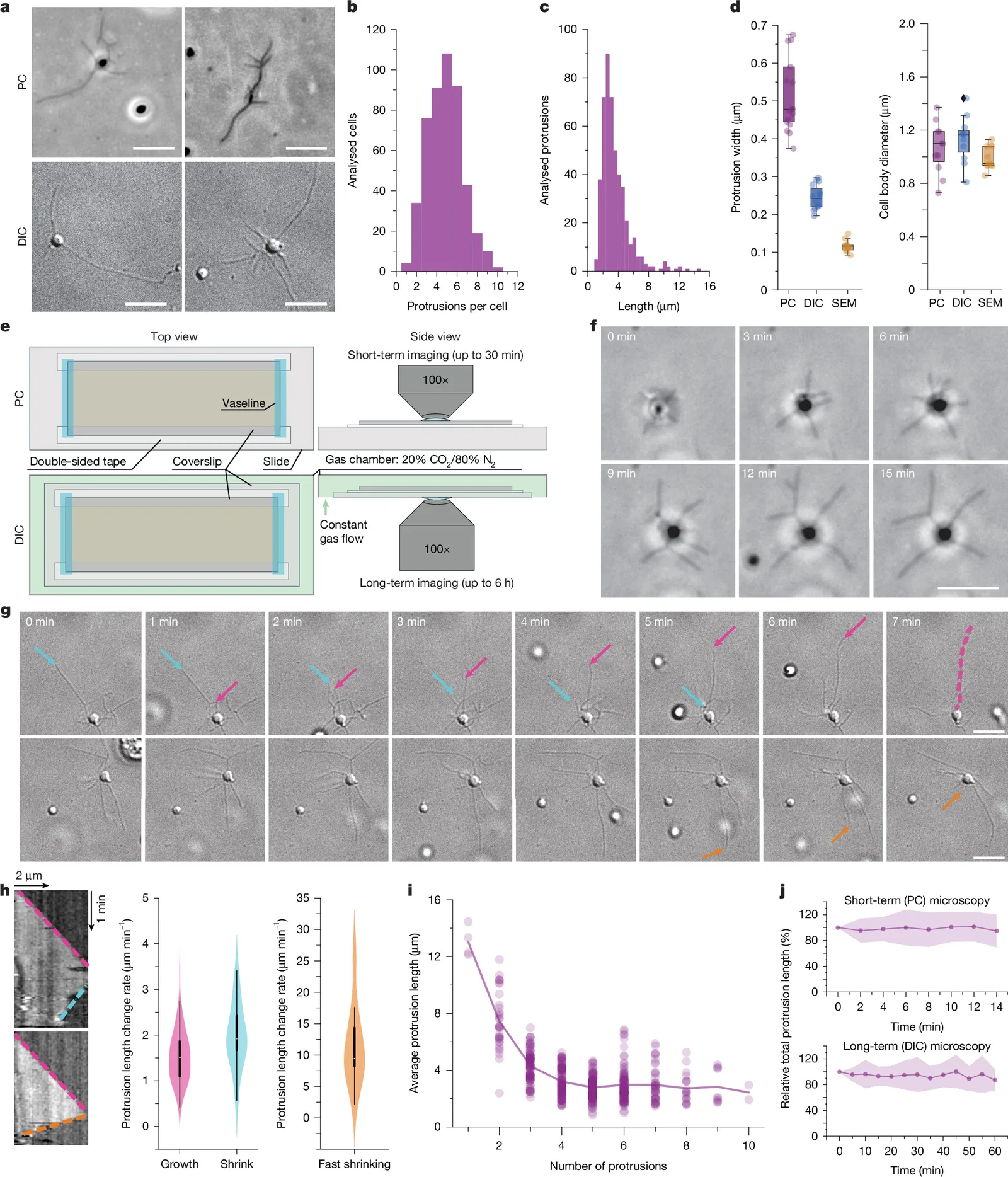
Anaerobic live-cell imaging of *Ca.* L. ossiferum reveals plastic cell shape. **a**, Representative micrographs from PC and DIC microscopy of L. ossiferum with different morphologies. **b**,**c**, Quantifications of protrusion numbers per cell (**b**) and protrusion lengths (**c**) from *n* = 479 cells. **d**, Quantification of the protrusion and cell body diameters from at least 10 cells using PC, DIC and scanning electron microscopy (SEM). Box plot components defined in the Methods (‘Image analysis’). **e**, Schematic of the anaerobic imaging setup with PC (top) for short-term imaging (up to 30 min) and DIC (bottom) for longer imaging experiments (up to 6 h). Representative experiments are shown in Supplementary Video 1. **f**, Representative PC time-lapse micrographs of an L. ossiferum cell adhering to the glass slide. The corresponding experiment is shown in Supplementary Video 2. **g**, DIC time-lapse micrographs of an L. ossiferum cell with growing and shrinking protrusions. The arrows indicate growing (pink), shrinking (cyan) and fast-shrinking (orange) protrusions. The dashed pink line is a representative line used for kymography. The corresponding experiment is shown in Supplementary Video 3. **h**, Representative kymographs of growing, shrinking and fast shrinking protrusions and the corresponding quantifications (*n* = 115 protrusions from 28 experiments of 13 different cultures). **i**, The average protrusion length and the number of protrusions per cell are inversely correlated (*n* = 479 cells). **j**, Quantification of the change in total protrusion length of single L. ossiferum cells in PC (top) and DIC (bottom) microscopy (*n* = 46 cells and 220 protrusions; thick line represents the mean and the shaded area represents the standard deviation). Scale bars, 5 μm (**a**,**f**,**g**). Source data

**Extended Data Fig. 1:**
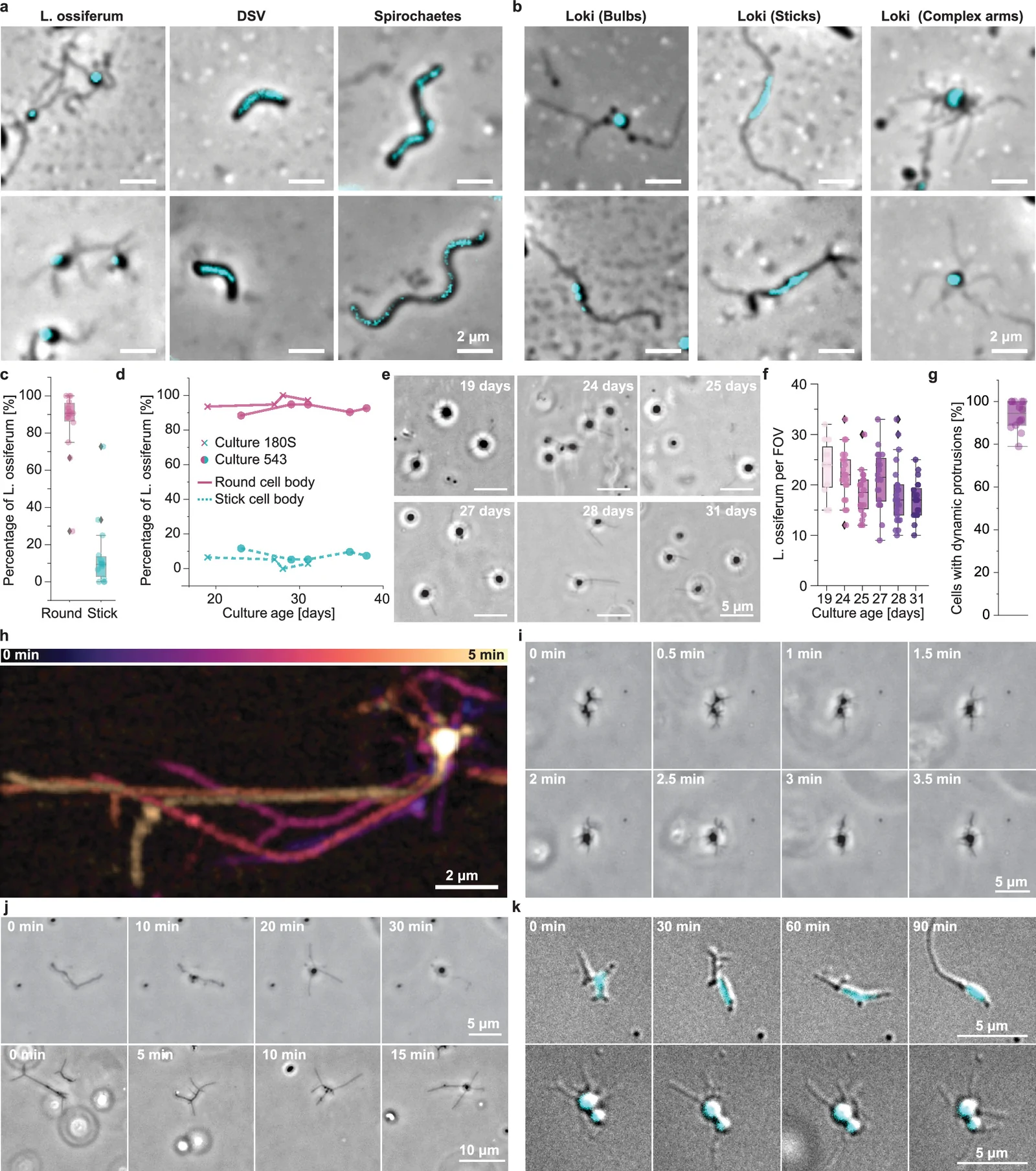
L. ossiferum undergo dynamic shape change on a minute time scale. **a**, Representative micrographs of glutaraldehyde (GA) fixed L. ossiferum and its syntrophic partners Halodesulfovibrio (DSV) and Spirochaetes stained with NucBlue (DNA, cyan). **b**, Representative micrographs of GA-fixed and NucBlue-stained L. ossiferum adopting different cell shapes. **c**, Percentage of round and stick shaped L. ossiferum cells in 9 cultures. **d**, Quantification of the relative abundance of round and stick-shaped L. ossiferum cells in two L. ossiferum cultures at different time points. **e**, Representative micrographs of one L. ossiferum culture repeatedly sampled at different time points. **f**, Quantification of the number of adhered L. ossiferum per field of view (FOV) at different time points. **g**, Quantification of the percentage of L. ossiferum with dynamic protrusions. **h**, A temporal projection of L. ossiferum, illustrating the rearrangement of protrusions over time. **i**,**j**, Representative phase contrast (PC) time - lapse micrographs of L. ossiferum cells undergoing dynamic shape changes. The corresponding experiments are shown in Video S4. **k**, Representative differential interference contrast (DIC) time-lapse micrographs showing L. ossiferum cells and their DNA-stained with NucBlue (cyan) either undergoing shape change (top) or retaining their shape (bottom). The corresponding experiment is shown in Video S5. Source data

L. ossiferum is cultivated as an enrichment culture^7^ and for imaging experiments we chose cultures with more than 1 × 10^6^ L. ossiferum cells per ml and a relative abundance of L. ossiferum of 20–60%. We found cells to adhere actively to glass surfaces within 2 h of incubation, which was more efficient than their syntrophic partners of the same culture (Halodesulfovibriae or Spirochaetes; Extended Data Fig. 1a). In this study, we describe the morphology and behaviour of adherent cells only, not including those that remained in the supernatant. Most adherent cells showed the characteristic morphology of a central cell body with protrusions extending in all directions (Fig. 1a). The number and length of protrusions varied greatly, with an average of five per adherent cell (from 1 to 10) and an average length of 3.5 µm (from 1 µm to 15 µm; Fig. 1b,c). Assuming protrusions as a regular tubus of 0.25 µm (0.2–0.3 µm) and a perfectly round cell body of 1.06 µm in diameter (0.8–1.4 µm) (Fig. 1d), we approximated the volume of a single protrusion to be one third of the cell body, but nearly doubling the available surface area. Thus, five protrusions of average length would increase the available surface area by 4.1-fold. Owing to the fineness of the protrusions, we are potentially overestimating the true increase in cell surface area, but even with the most conservative estimates from cryo-EM (average protrusion diameter of 83 nm; ref. ^7^), the presence of five protrusions doubles the available surface area. Apart from the above-described morphology, we also found L. ossiferum with deviating shapes, that is, particularly elongated, stick-like cells that were lacking a central cell body. They constituted the minority of cells (10.4 ± 13.0%; Fig. 1a and Extended Data Fig. 1b,c). Repeated sampling over the lifetime of a single culture showed that these morphological characteristics were largely stable (Extended Data Fig. 1d). The total number of attached L. ossiferum cells declined slightly over the course of 12 days (Extended Data Fig. 1e,f). The different cell shapes we observed suggested either the presence of different cell subtypes or a dynamic rearrangement of the cell morphology. To differentiate between these possibilities, we established time-lapse microscopy by upright phase contrast (PC) and inverted differential interference contrast (DIC) microscopy (Fig. 1e and Supplementary Video 1). For both methods, the flow chambers were prepared in anaerobic conditions in an N_2_:CO_2_ atmosphere and then sealed. PC microscopy was used without environmental control of the atmosphere for short-term experiments, lasting at most 30 min. For DIC microscopy, the surrounding atmosphere of the flow chamber was kept at 85:15% N_2_:CO_2_ by constant gas flow into the chamber, and experiments were recorded for up to 6 h. On contact with the glass surface, L. ossiferum cells rapidly spread their protrusions while remaining anchored on the coverslip (Fig. 1f and Supplementary Video 2). Despite the slow doubling time of the organism (about 7 days), cells changed their morphology rapidly on a minute time scale. In many cases, the growing protrusions branched off to form side arms resulting in two or sometimes more endings per protrusion. Moreover, protrusions grew and retracted constantly, possibly reacting to their environment (Fig. 1g, Extended Data Fig. 1g,h and Supplementary Video 3). Single protrusions reached up to 15 µm before they retracted either slowly or in a fast manner. We used kymography to quantify these dynamics and found protrusions to grow at 1.5 ± 0.6 µm min^−1^, and retractions to occur in two distinct categories, either at 2.0 ± 0.7 µm min^−1^ or at 11.4 ± 6.1 µm min^−1^ (Fig. 1h). The number and length of protrusions of adhered L. ossiferum cells were inversely correlated, suggesting precise control over the membrane area available for protrusion formation (Fig. 1i). During the observed rearrangements, the total protrusion length of single cells remained constant, further suggesting a controlled redistribution of the available membrane lipids. (Fig. 1j). Finally, we observed substantial shape changes of L. ossiferum cell bodies: merging two cell-body-like structures into one (Extended Data Fig. 1i) or switching from a stick-like to a spherical appearance (Extended Data Fig. 1j and Supplementary Video 4). These morphological changes were not restricted to the cell body alone, as the position of the chromosome, revealed by live staining with NucBlue, occasionally rearranged dynamically (Extended Data Fig. 1k and Supplementary Video 5).

## L. ossiferum protrusions drive migration

**Fig. 2:**
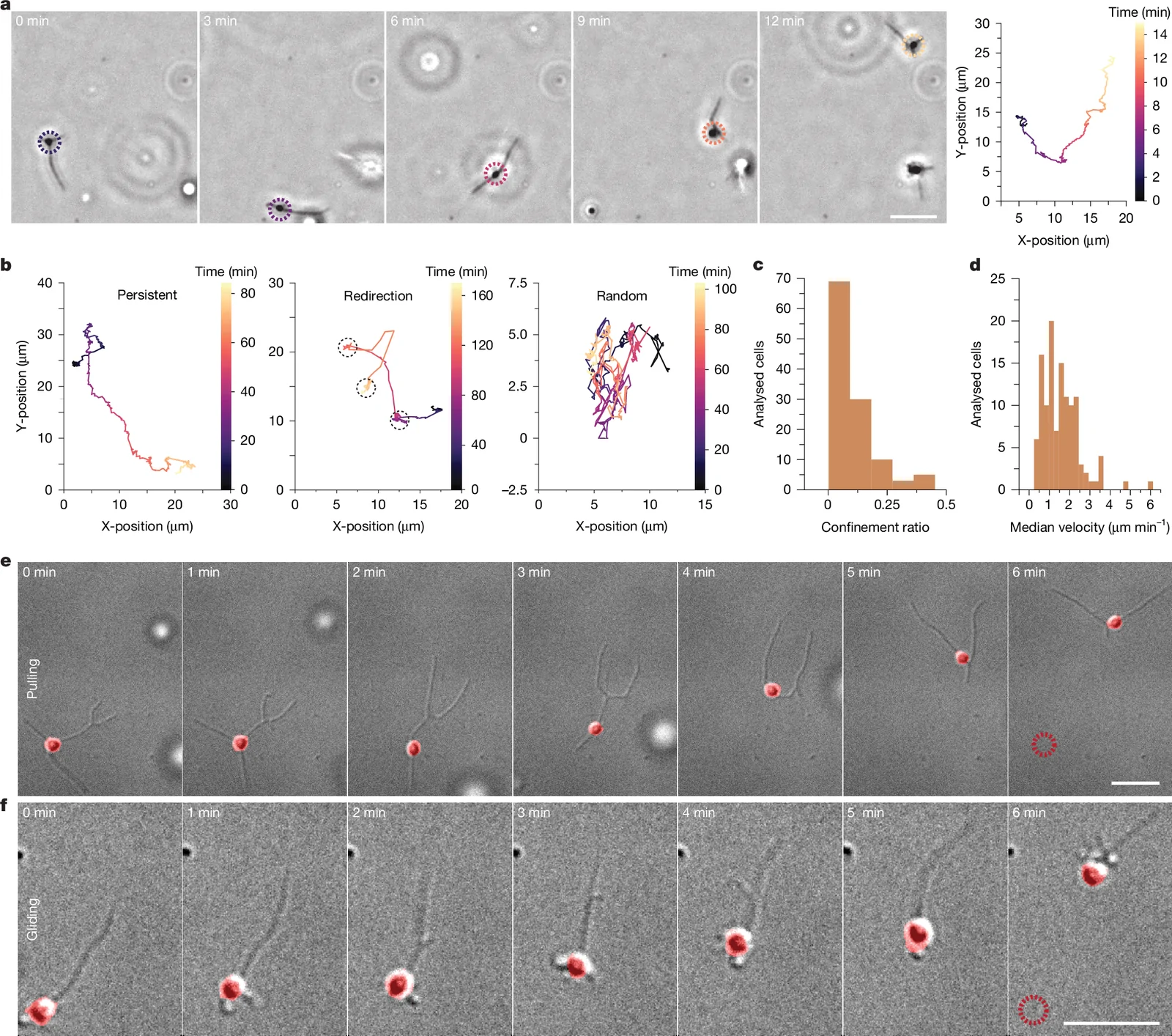
L. ossiferum migrates along glass by protrusion-based crawling motility. **a**, Representative PC time-lapse micrographs of migrating L. ossiferum and the corresponding trajectory. The dashed circles indicate the position of the cell body at different time points in the track. **b**, Example trajectories of representative L. ossiferum cells imaged with DIC microscopy moving persistently (left), undergoing several redirections (centre) and moving completely randomly (right). **c**,**d**, TrackMate quantification of the confinement ratio (**c**) and the median instantaneous velocity (**d**) of L. ossiferum (*n* = 117 cells from nine cultures). **e**,**f**, Representative DIC micrographs of L. ossiferum displacing their cell body via pulling (**e**) and gliding (**f**). The cell body is shown in red, and the red dashed circles (6 min time points) indicate the respective starting points of each cell body. The corresponding experiments are shown in Supplementary Videos 6 and 7. Scale bars, 5 μm (**a**,**e**,**f**). Source data

**Extended Data Fig. 2:**
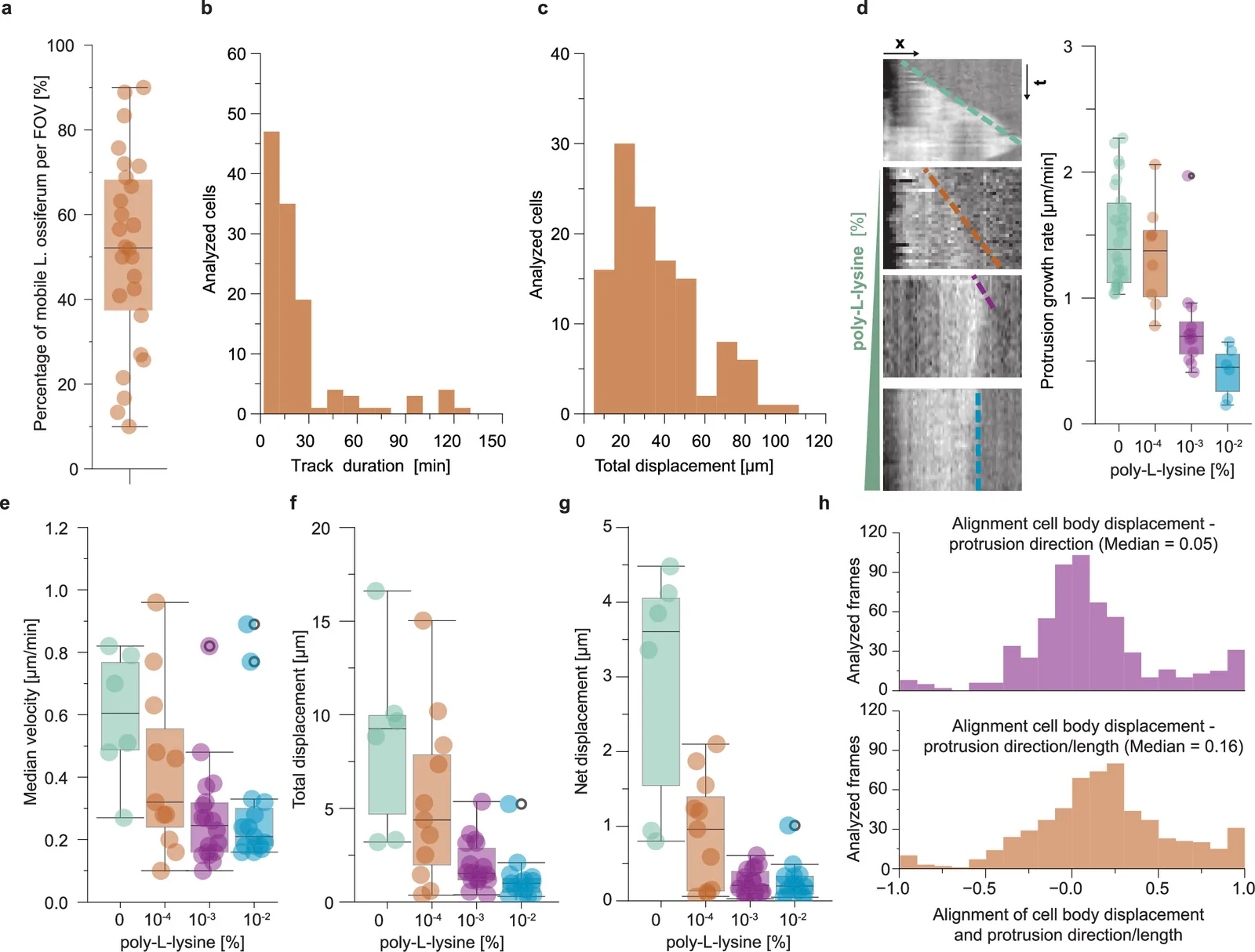
Poly-l-lysine and protrusion effect on L. ossiferum mobility. **a**, Quantification of the percentage of mobile L. ossiferum per field of view from 12 different cultures. **b**,**c**, Quantification of the track duration (**b**) and total displacement (**c**) of motile L. ossiferum cells. **d**, Representative kymographs of protrusions of L. ossiferum adhered to glass with increasing concentrations of poly-l-lysine and the corresponding quantification. Experiments were performed with two cultures and at least 6 protrusions were quantified for each condition. **e**–**g**, TrackMate quantification of L. ossiferum cells adhered to glass coated with increasing concentrations of poly-l-lysine measuring the median instantaneous velocity (**e**), the total displacement (**f**) and the net (start to end) displacement (**g**). **h**, Histograms of alignments between the displacement vector of cell bodies and the direction vector of protrusions considering the mean alignment across all protrusions (**top**) and considering the protrusion lengths (**bottom**). The measured alignment ranges from −1 (protrusions opposing movement) to +1 (protrusions aligned with movement). (n = 19 cells and 588 frames). Examples of the segmentation for this analysis are shown in Video S8. Source data

Apart from the plastic cell morphology and the rearrangement of protrusions, we found a considerable fraction of L. ossiferum translocating their central cell body (51.4 ± 22.5%; Extended Data Fig. 2a). We were able to distinguish distinct types of movement. On the one hand, sudden displacements of the cell body were observed over larger distances because of detachment and rapid forward pulling by attached protrusions in the direction of migration (Supplementary Video 6). On the other hand, comparably smooth and continuous movement over shorter distances appeared, reminiscent of the gliding or crawling motility observed in eukaryotes (Fig. 2a and Supplementary Videos 6 and 7). Most mobile cells migrated randomly over the glass surface, constantly changing directions, as reflected in a low confinement ratio (measuring the net displacement of the cell body from its starting point relative to total path length) (Fig. 2b,c). Only a small subset of L. ossiferum exhibited persistent motion into one direction (Supplementary Video 7, 6 of 117 cells). We measured the median speed of migrating L. ossiferum cell bodies to be 1.57 ± 0.93 µm min^−1^ (Fig. 2d and Extended Data Fig. 2b,c). This is much slower than swimming motilities exhibited by archaea using an archaellum (0.18–22.8 mm min^−1^; ref. ^28^), flagellated bacteria (1.5 mm min^−1^; ref. ^29^), protists using cilia or flagella (7–42 mm min^−1^; ref. ^30^) or bacterial twitching motility (60 µm min^−1^; ref. ^31^). Instead the motility of L. ossiferum is within the range of reported crawling speeds of eukaryotic cells (0.1–20 µm min^−1^; refs. ^32,33^) and the type IV pili-driven twitching motility of *Sulfolobus acidocaldarius* (1.1 µm min^−1^), the hitherto only described surface motility in Archaea^34^. Although twitching motility is dependent on the formation of extracellular pili proteins, the migration of L. ossiferum seemed dependent on the formation and retraction or pulling of membrane protrusions that are best characterized as thin, tube-shaped pseudopodia. The median migration velocity of cell bodies was remarkably similar to protrusion growth rates, indicating a potential connection between those motility parameters. When coating the glass with incrementally increasing poly-l-lysine concentrations, a concomitant decrease in both protrusion dynamics and migration speed was observed (Extended Data Fig. 2d–g), which further suggests potential mechanistic coupling between the efficiency of pseudopod protrusion and cell-body translocation. We investigated three conceivable mechanical modes of force transduction that can drive crawling motion: pulling, gliding and pushing. Microscopic observations suggest that L. ossiferum cells glide and pull. Pulling cells adhered with the tip of leading protrusions, and the cell body followed, sometimes after detaching a protrusion at the rear of the cell body (Supplementary Video 6). Furthermore, pulling was accompanied by the shortening of leading protrusions, as if membrane protrusions at the front acted as adhesive springs (Fig. 2e). Gliding motion occurred in the absence of these sudden shape changes, probably because the force transduction happened at a molecular scale, either by treadmilling of actin filaments^1,35–37^ or by simultaneous polymerization and depolymerization in front and rear of the cell, respectively. The latter movement dominated in cells with persistent motion, in which cell bodies followed a single protrusion pointed towards the direction of movement (Fig. 2f and Supplementary Video 7). To corroborate this view, we quantified the alignment of cell body movement with the direction into which protrusions extend (*n* = 19 cells). We found the movement direction to be predominantly determined by protrusions in front of the cell body and little evidence for pushing. The alignment was improved when also taking the length of protrusions into account (Extended Data Fig. 2h and Supplementary Video 8).

## Dynamics depend on Lokiactin filaments

**Fig. 3:**
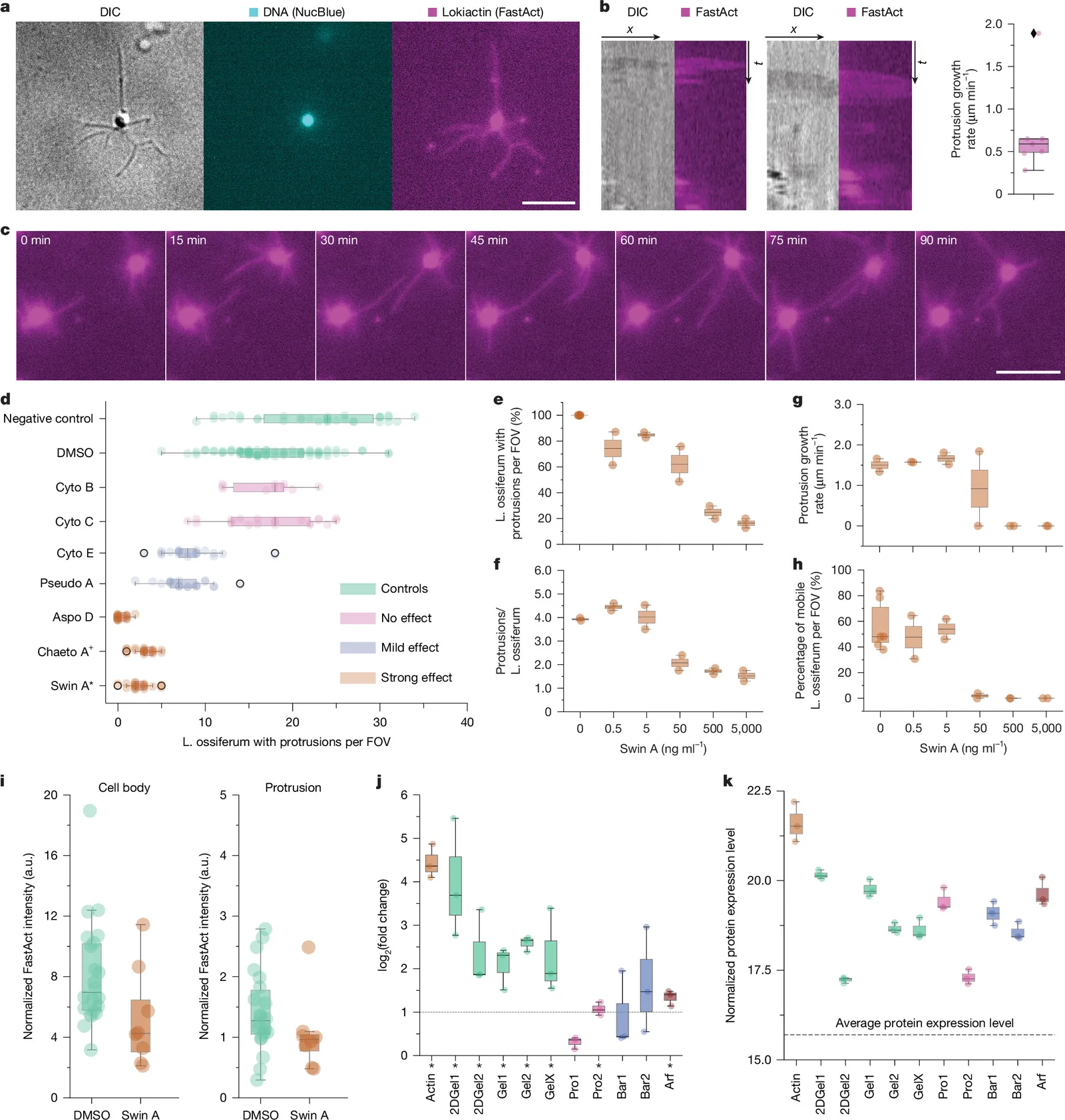
Protrusion dynamics require Lokiactin polymerization and core actin binding proteins. **a**, Representative DIC micrographs of L. ossiferum stained with NucBlue (DNA, cyan) and FastAct (Lokiactin, magenta). **b**, Representative kymographs of growing protrusions in DIC and FastAct channels and the corresponding quantification (*n* = 7 protrusions from 6 cells). **c**, Representative time-lapse micrographs of FastAct-stained L. ossiferum rearranging their protrusions (Supplementary Video 9). **d**, Effects of actin inhibitors on the number of adherent L. ossiferum with protrusions per field of view (FOV). The inhibitor concentrations were 25 (+), 12.5 or 5 (*) µg ml^−1^. Each dot represents all L. ossiferum cells in a single FOV. Experiments were performed with one L. ossiferum culture to select promising inhibitors for further characterization and titration (Supplementary Table 1). **e**–**h**, Effects of increasing Swin A concentrations on the number of L. ossiferum cells with protrusions per FOV (**e**), the number of protrusions per cell (**f**), the growth rate of protrusions (**g**) and the percentage of mobile L. ossiferum (**h**). Titrations were performed in duplicates with two cultures: 325 cells with protrusions counted and kymographs from 43 cells quantified. **i**, Quantification of normalized FastAct intensities in cell bodies and protrusions of L. ossiferum treated with DMSO or 100 ng ml^−1^ Swin A (*n* = 23 for DMSO and *n* = 8 for Swin-A-treated cells). **j**, Box plot showing the fold change of proteins detected by Co-IP experiments using a Lokiactin antibody. Proteins marked with an asterisk are significantly enriched (log_2_(fold change) < 1, *P* < 0.05; *n* = 3 cultures). **k**, Boxplot depicting the mean expression levels of proteins identified in **j** compared with the average expression level of all proteins from three L. ossiferum cultures (grey line). NCBI accession codes of proteins in **j** and **k** can be found in Supplementary Table 2. Scale bars, 5 μm (**a**,**c**). Source data

**Extended Data Fig. 3:**
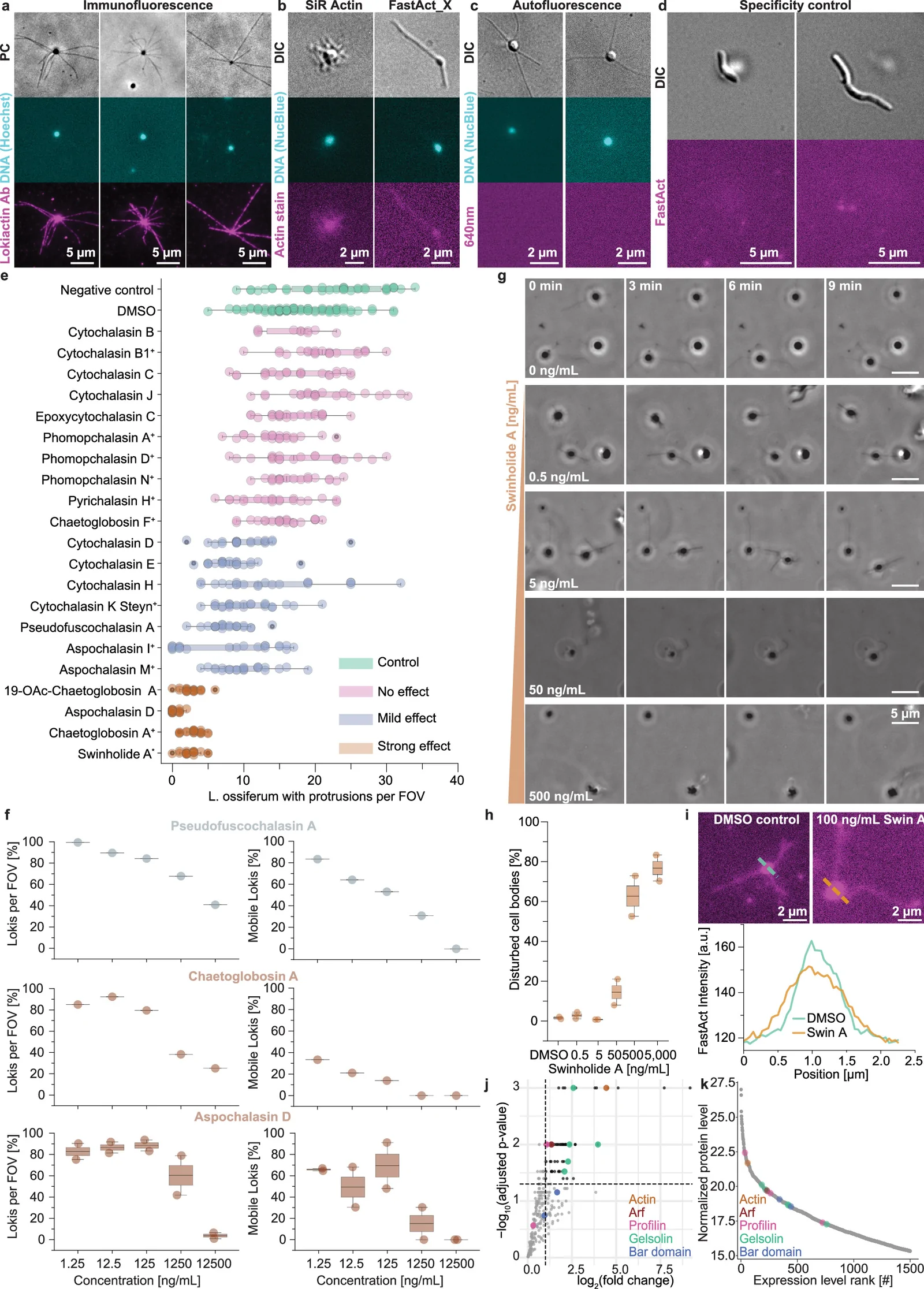
Controls for fluorescent imaging and actin inhibitor effects on L. ossiferum. **a**, Representative micrographs of immunofluorescence experiments of L. ossiferum stained with Hoechst (cyan) and a Lokiactin antibody (magenta). **b**, Representative micrographs of L. ossiferum cells stained with NucBlue, SiR-Actin and FastAct_X as indicated. **c**, Autofluorescence controls of DNA-stained L. ossiferum. **d**, Specificity control of the FastAct-stain on the syntrophic organisms within the culture. Halodesulfovibrio and Spirochaetes show no visible FastAct signal. **e**, List of all tested inhibitors of eukaryotic actin assembly and their effects on the number of adherent L. ossiferum cells per field of view (FOV, 124*93 µm). Each dot represents the number of L. ossiferum cells in a single FOV. Experiments were performed with one L. ossiferum culture on three subsequent days to select candidate inhibitors for further characterization and titration. The final concentrations used were 25 (+), 12.5 and 5 (*) µg/mL (Supplementary Table 1). **f**, Titrations of three selected actin inhibitors and a quantification of the average number of adherent L. ossiferum cells with protrusions (**left**) and their mobility (**right**). **g**, Representative micrographs of L. ossiferum in the presence of increasing concentrations of Swinholide A (Video S10). **h**, Quantification of the percentage of disturbed (=not spherical) cell body morphologies with increasing concentrations of Swinholide A. **i**, Representative micrographs of FastAct-stained L. ossiferum cells without (**left**) and with (**right**) 100 ng/mL Swinholide A and the line scan measurements of FastAct signal (**bottom**) from dashed, coloured lines in the micrographs. **j**, Volcano plot of proteins identified in Co-IP experiments. Proteins in the top right corner are enriched significantly (log_2_ fold change > 1, p-value > 0.05). **k**, Expression plot of the top 1500 expressed proteins with proteins identified in Co-IPs highlighted. Source data

Next, we investigated if and how far filaments formed by Lokiactin, which are consistently found in protrusions of L. ossiferum (Extended Data Fig. 3a; refs. ^7,38^), are involved in the observed cell dynamics. In the absence of genetic tools and fluorescent fusion proteins, we first tried to visualize Lokiactin with fluorescent stains developed for eukaryotic actin. SiR Actin or a derivative thereof, FastAct_X, showed a very weak fluorescent signal, suggesting a weak affinity to Lokiactin filaments (Extended Data Fig. 3b). Another SiR Actin derivative (FastAct) improved staining and signal on L. ossiferum, while retaining high specificity (Fig. 3a and Extended Data Fig. 3c,d). Although the rates of protrusion growth dropped (0.72 ± 0.49 µm min^−1^; Fig. 3b), protrusions still rearranged dynamically (Fig. 3c and Supplementary Video 9) and actin stain was observed in all dynamic protrusions as well as in the central cell body. Then, we wanted to arrest the polymerization dynamics of Lokiactin and tested different inhibitors of eukaryotic actin polymerization. Most of the tested compounds were cytochalasans, which are thought to bind to the fast-growing barbed ends of actin filaments, thus disrupting their polymerization^39^. Swinholide A (Swin A) is a macrolide toxin, shown to sequester actin dimers and sever eukaryotic actin filaments^40^. In initial tests, we used high concentrations of actin inhibitors to identify a compound suitable for L. ossiferum cells and avoid overlooking effects due to compromised affinities of the inhibitors to Lokiactin. Although some commonly used inhibitors, such as Cytochalasin B, affected the dynamics or morphology only slightly, we found three inhibitors (two cytochalasans, Aspochalasin D, Chaetoglobosin A and the macrolide toxin Swin A) to significantly reduce the number of L. ossiferum cells with protrusions adherent to glass (Fig. 3d, Extended Data Fig. 3e and Supplementary Table 1). We performed a more detailed titration of these inhibitors and found Swin A to affect L. ossiferum at concentrations as low as 50 ng ml^−1^ (36 nM), which is within the range of concentrations used in eukaryotic cells^40^ (Fig. 3e and Extended Data Fig. 3f,g). We analysed the Swin A effect in more detail and found the inhibitor to affect the morphology of the cell body (Extended Data Fig. 3h) and decrease the number of protrusions per cell to only one or two remaining ones (Fig. 3f). Moreover, the formation of new protrusions and growth of persisting ones were already arrested at 50 ng ml^−1^ Swin A (Fig. 3g). Finally, L. ossiferum lost the ability to migrate and remained stuck in place with Swin A concentrations at or above 50 ng ml^−1^ Swin A (Fig. 3h). Some cells remained attached with a single protrusion that lost internal stabilizing filaments. These protrusions became increasingly curved and swayed strongly with Brownian motion, which was in stark contrast to the controlled protrusion dynamics observed without the inhibitor (Supplementary Video 10). When we combined fluorescence microscopy of FastAct-stained Lokiactin with Swin A, we found the fluorescent signal in cell bodies and protrusions to decline but not disappearing completely (Fig. 3i and Extended Data Fig. 3i). Together, these inhibitor experiments suggest that protrusion dynamics and movement of cells are dependent on Lokiactin filaments. Next, we searched for candidate proteins that might regulate the dynamics of the Lokiactin cytoskeleton. In eukaryotic cells, more than 150 actin-binding proteins (ABP) are used to fine-tune actin polymerization. On the basis of comparative genomic studies, Asgard archaea seem to rely mainly on gelsolins and profilins, whose potential to regulate nucleation, bundling and severing of actin filaments has been shown in biochemical in vitro assays^25–27^. These homologues were identified in most of the eight closed, seven chromosome-level and 396 Asgard meta-genomes (according to GTDB in 2025)^3,41^ with up to five profilin and nine gelsolin homologues present in a single Asgard genome^24^. L. ossiferum encodes for nine proteins with gelsolin homology (GH) domains and three profilins. To confirm potential interactors with Lokiactin in vivo, we performed co-immunoprecipitation (Co-IP) with a previously validated Lokiactin antibody^7^. Apart from Lokiactin itself (log_2_(fold change) of 4.44), one profilin, five gelsolin homologues, as well as an ARF-GTPase were found to be significantly enriched (log_2_(fold change) > 1, *P* < 0.05). A second profilin and two BAR-domain proteins were also detected, although they did not reach statistical significance (Fig. 3j, Extended Data Fig. 3j and Supplementary Table 2). ARF GTPases and BAR-domain proteins in eukaryotes can couple membrane remodelling to actin cytoskeleton dynamics, with ARF GTPases regulating phosphoinositide signalling and actin nucleation pathways, and BAR proteins binding to sites of membrane deformations and recruiting actin regulatory factors^42,43^.

When exploring the abundance of these proteins within cells by analysing total cell protein contents of three L. ossiferum cultures, we found most gelsolins and profilins to rank among the top 500 of 3,061 expressed proteins (Fig. 3k, Extended Data Fig. 3k and Supplementary Table 2). A more detailed description of the identified proteins and a phylogenetic comparison with previously biochemically characterized gelsolins and profilins are found in the Supplementary Discussion and Supplementary Figs. 1 and 2. Together, these results indicate that membrane deformations and motility in Asgard archaea are controlled by a dynamic Lokiactin cytoskeleton, probably regulated by gelsolins and profilins.

## Dynamics are conserved among Asgard archaea

**Fig. 4:**
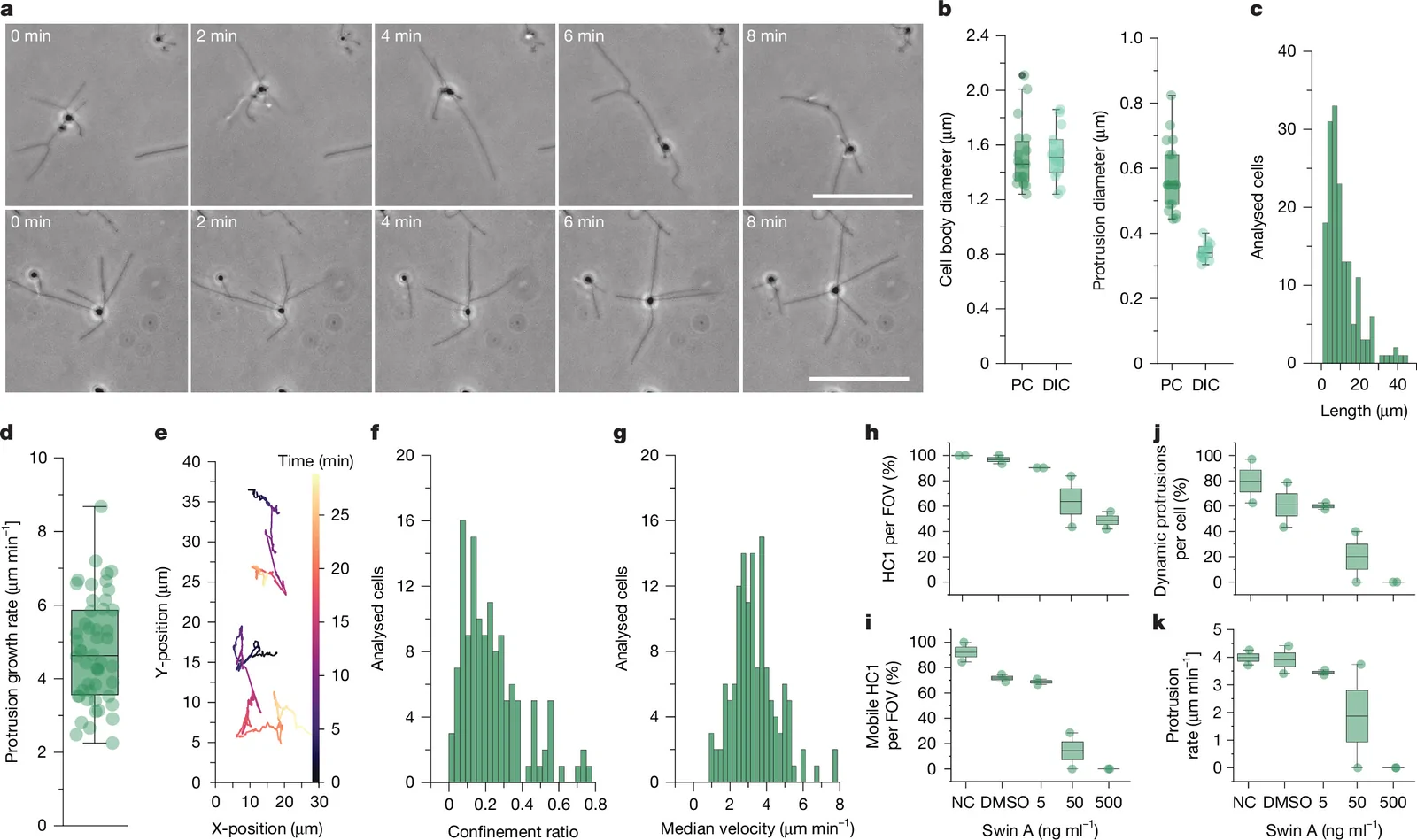
M. peptidophilum exhibits actin-dependent morphological rearrangements and surface motility. **a**, Representative PC time-lapse micrographs of M. peptidophilum cells rearranging their protrusions and migrating over the glass. The corresponding experiments are shown in Supplementary Video 11. **b**, Quantification of the diameter of cell bodies (left) and protrusions (right) from PC and DIC microscopy (*n* = 13 and 18 cells, respectively). **c**, Quantification of the protrusion length (*n* = 49 cells and 166 protrusions). **d**, Quantification of the protrusion growth rate by kymography (*n* = 50 protrusions from 30 cells and 6 cultures). **e**, Representative trajectories of two migrating cells. **f**,**g**, TrackMate quantification of the confinement ratio (**f**) and the migration speed (**g**) of migrating M. peptidophilum cells (*n* = 133 cells from 5 cultures). **h**–**k**, Effects of increasing concentrations of Swin A on the number of adherent cells with protrusions per FOV (**h**), the percentage of mobile M. peptidophilum per FOV (**i**) or of dynamic protrusions per cell (**j**) and the growth rate of protrusions (**k**). Experiments without DMSO or Swin A are labelled NC (negative control), and titrations were performed in duplicates with two cultures, and 118 cells with protrusions were counted and kymographs from 22 cells quantified. Scale bar, 20 μm (**a**). Source data

**Extended Data Fig. 4:**
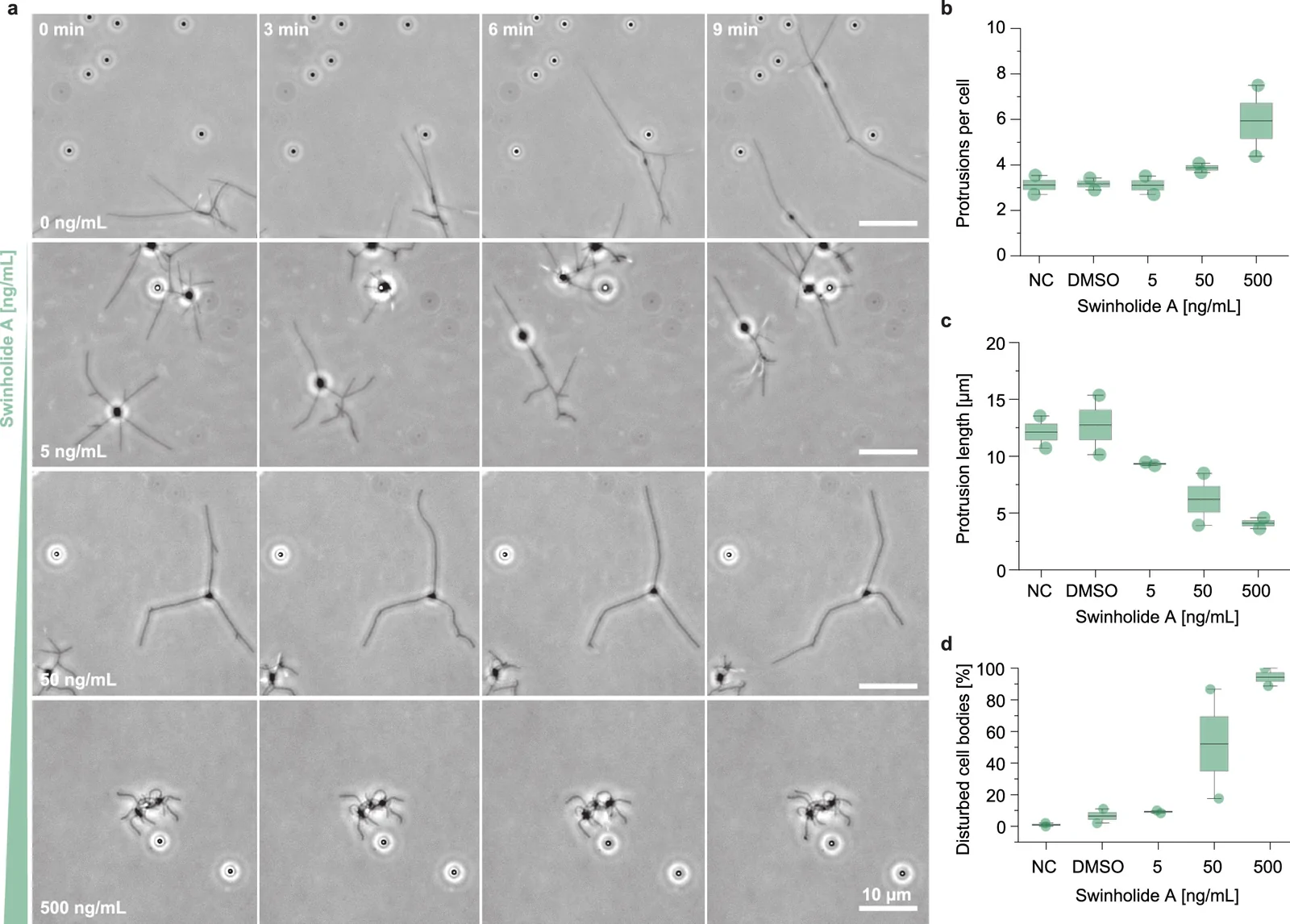
Swinholide A arrests dynamic rearrangements of M. peptidophilum. **a**, Representative phase contrast (PC) time-lapse micrographs of M. peptidophilum in the presence of increasing concentrations of Swinholide A. The corresponding experiments are shown in Video S12. **b**–**d**, Quantification of the number of protrusions per cell (**b**), the average length of protrusions (**c**) and percentages of disturbed M. peptidophilum cell bodies (**d**) with increasing Swinholide A concentration. Titrations were performed in duplicates with two M. peptidophilum cultures. Experiments without DMSO or Swinholide A are labelled NC (negative control). Source data

Next, we were interested in whether the observed dynamics are a common characteristic of Asgard archaea and performed experiments with the recently cultivated *Ca*. M. peptidophilum strain HC1^9^. This strain is a representative of the Hodarchaeales lineage and was isolated as a pure co-culture with Methanocalculus sp. MC2 with a stable population ratio of HC1:MC2 = 70:30% (ref. ^9^). As observed for L. ossiferum, M. peptidophilum cells rearranged their morphology drastically (Fig. 4a and Supplementary Video 11). In alignment with previous observations^9^, the cell body and protrusions were bigger and longer, respectively, than in L. ossiferum (Fig. 4b,c). When we measured protrusion dynamics, we found them to grow approximately three times faster than protrusions of L. ossiferum (4.8 ± 1.4 µm min^−1^ compared with 1.5 ± 0.6 µm min^−1^; Fig. 4d). M. peptidophilum cells also migrated along the glass randomly (confinement ratio 0.24 ± 0.17; Fig. 4e,f) and, in concordance with faster protrusion dynamics, displaced their cell body faster than L. ossiferum (3.4 ± 1.2 µm min^−1^ compared with 1.6 ± 0.9 µm min^−1^; Fig. 4g). We then performed a titration with Swin A to see if the motility of M. peptidophilum cells is also dependent on a dynamic actin cytoskeleton (Extended Data Fig. 4a). We found a decrease in the density (Fig. 4h) and motility (Fig. 4i) of adherent M. peptidophilum cells, similar to experiments with L. ossiferum. We did not find a reduction in the number of protrusions per cell, but we did find a decreased average protrusion length (Extended Data Fig. 4b,c). Furthermore, Swin A blocks the dynamic rearrangement of spherical to elongated cell bodies (and vice versa) and seems to generally cause a collapse of spherical cell bodies, quantified as percentages of disturbed cell bodies (Extended Data Fig. 4d). The formation of new protrusions and growth of persisting protrusions was arrested with concentrations higher than 50 ng ml^−1^ Swin A (Fig. 4j,k). Thus, the presence of Swin A arrested the rearrangement of cell morphology and migration as observed in L. ossiferum (Supplementary Video 12).

## Discussion

In this study, we established anaerobic live-cell microscopy of L. ossiferum and M. peptidophilum, representing two different lineages of Promethearchaeota. We find these cells to dynamically rearrange their cell bodies and constantly form and retract protrusions, which are often branched. This plasticity is unprecedented in prokaryotes and usually associated with eukaryotic cells^44,45^. As the membrane surface appeared constant, these shape changes do not seem to be facilitated by synthesis of new lipids, uptake of lipids from syntrophic partners or externalization of intracellular lipid compartments, such as caveolae^46^. Furthermore, the cells exhibit a unique type of prokaryotic motility, akin to amoeboid or crawling motion performed by immune cells or certain protists^47^. This motility is distinct from the swimming motion in archaea and bacteria, driven by an archaellum or flagellum, respectively^48^, and the twitching motility described in S. acidocaldarius, which is driven by type IV pili retraction^34^. Instead, L. ossiferum and M. peptidophilum use their dynamic protrusions to move while adhered to a surface, with a balance of adhesion strength and detachment being important for the migratory behaviour, as our experiments with poly-l-lysine suggest. Both the membrane rearrangements and the crawling motility involve the Lokiactin cytoskeleton, as we find several inhibitors of actin to arrest these dynamics. The various ranges of effects of different (eukaryotic) actin inhibitors may be caused by differences in protein sequence, the filament ultrastructure or perhaps impaired cell permeability. Different from other types of surface motility found in bacteria or archaea^34,49^, the motility in Asgard archaea depends on an internal cytoskeleton. The observed enormous membrane plasticity and crawling motility could be beneficial in the natural environment of Asgard archaea, the interstices of marine sediment^50^, as it might allow them to scan their environment for nutrients or interaction partners or to facilitate more efficient nutrient uptake.

Based on the similarities between the detected gelsolin and profilin homologues and those previously studied from other Asgard archaea^25–27^, we imagine the Lokiactin cytoskeleton to be dynamically regulated. However, we expect this regulation to be more intricate, as suggested, for instance, by the presence of BAR domain proteins or an ARF GTPase in our Co-IP experiments. Understanding Lokiactin polymerization and how it is affected by profilin and gelsolins requires more biochemical investigations and will be the subject of future work. The presence of Lokiactin, gelsolin and profilins in virtually all genomes of Asgard archaea^3^ strongly suggests their presence in the last common Asgard archaea ancestor. Therefore, archaea probably evolved the ability to use an actin cytoskeleton for motility early with the emergence of the Asgard archaea lineage and before the emergence of eukaryotes^12,17^. Current understanding of actin-based cell migration is heavily biased towards metazoans and amoebozoans with a more complex migration regulation machinery, including motor proteins, that is, myosins and filament polymerases such as formins or Ena-VASP family members^51–54^. However, examples of motility in the absence of these proteins are known, as in red algae and in *Trichomonas vaginalis*, but understood in less detail^55,56^. Further investigations on the mechanisms of Asgard archaea motility should thus give insights into an ancient form of actin-based cell migration. The described plasticity and motility might have played an important part in eukaryogenesis, the emergence of eukaryotes, which remains one of the great enigmas in evolution. Current models include a merger of an Asgard archaeon with at least one bacterial partner^10–12,57–59^, in which the bacterium is eventually engulfed by the archaeon. This process may necessitate plasticity of the archaeal membranes and cell shape, as shown by our study with L. ossiferum and M. peptidophilum. Although this study was performed with two extant model organisms that will differ from the archaeal ancestor of eukaryotes 2 billion years ago, it is conceivable that the latter used protrusions to move towards potential interaction partners, connect to them to facilitate nutrient exchange and eventually engulf them. Observing Asgard archaea for an extended time in anaerobic conditions was a first step in understanding their dynamics and migration capacities. Future studies will aim at revealing the principles behind the observed behaviour and hopefully shed light on the events that happened about 2 billion years ago, probably in marine sediments^60^, when the first eukaryotic cell arose.

## Methods

### Cultivation of *Ca*. L. ossiferum and M. peptidophilum

*Ca*. L. ossiferum enrichment cultures were grown protected from light in lokiarchaeal media in sealed, pressurized serum bottles in an 80:20% N_2_:CO_2_ atmosphere (0.3 bars) at 20 °C without shaking, as described previously^7^. The growth medium composition (per litre) was as follows: 20.7 g NaCl, 5 g MgCl_2_·6H_2_O, 2.7 g NaHCO_3_, 1.36 g CaCl_2_·2H_2_O, 0.54 g NH_4_Cl, 0.14 g KH_2_PO_4_, 0.03 g Na_2_S·9H_2_O, 0.03 g cysteine·HCl, 0.5 ml of acid trace element solution, 0.5 ml of alkaline trace element solution, 1 ml Se/W solution, 0.1% casein hydrolysate (w/v). The acid trace element solution contained (per litre): 1.49 g FeCl_2_·4H_2_O, 0.062 g H_3_BO_3_, 0.068 g ZnCl_2_, 0.017 g CuCl_2_·H_2_O, 0.099 g MnCl_2_·4H_2_O, 0.119 g CoCl_2_·6H_2_O, 0.024 g NiCl_2_·6H_2_O and 4.106 ml of 37% HCl. The alkaline trace element solution contained (per litre): 0.017 g Na_2_SeO_3_, 0.033 g Na_2_WO_4_, 0.021 g Na_2_MoO_4_ and 0.4 g NaOH. The pH was adjusted to 7.5, and the medium was supplemented with ampicillin, kanamycin and streptomycin (200 μg ml^−1^ each). The cultures were continuously passaged into serum flasks containing fresh medium—typically using an early stationary-phase culture as inoculum (1:10 dilution). Growth of L. ossiferum cultures was monitored by qPCR using lokiarchaeal-specific primers for 16S rRNA as described previously^7^. *Ca*. M. peptidophilum HC1 was grown in casamino acids–peptone–yeast extract medium in sealed serum bottles in an N_2_ atmosphere at 30 °C as described^9^. qPCR results were used to select cultures for live-cell imaging, and only cultures with densities above 1 × 10^6^ L. ossiferum per ml and a relative abundance of L. ossiferum above 20% were chosen. Cultures were in late exponential or stationary phase and between 15 days and 40 days old. Live imaging of M. peptidophilum was performed on cultures in the late exponential to early stationary phases with a population ratio of HC1:MC2 = 70:30%.

### Immunofluorescence and microscopy of fixed cells

For immunofluorescence experiments, L. ossiferum cultures were transferred onto poly-l-lysine (0.01%)-coated coverslips and incubated for 45 min in anaerobic conditions. Cells were then fixed with 3.7% PFA in PEM buffer (80 mM PIPES, 2 mM MgCl_2_ and 0.5 mM EGTA at pH 6.9). The cells were permeabilized with 0.1% Triton X-100 and blocked with 1% BSA solution in PBS-T. The primary antibody against Lokiactin and the secondary antibody (goat anti-rabbit AlexaFluor488, abcam ab150077) + 1 mg ml^−1^ Hoechst were diluted 1:1,000 in PBS-T + 0.1% BSA and either incubated for 1 h (primary) or 0.5 h (secondary) at room temperature, protected from light. Meanwhile, coverslips were washed gently with PBS by changing the solution twice. Finally, the coverslips were mounted using the VECTASHIELD antifading agent. The Lokiactin antibody was raised against a Lokiactin-specific peptide (CTFYTDLRVDPSEHPV) and previously validated by ELISA, western blot and immunogold localization^7^. To compare DNA signals and morphologies of L. ossiferum and their symbionts, cultures were fixed with 0.5% glutaraldehyde for 20 min, and DNA was simultaneously stained with Hoechst 33342 (1:1,000 dilution of 1 mg ml^−1^ stock solution). After fixation, cells were centrifuged 2× for 10 min at 15,000*g*, and the resulting pellet was resuspended in Milli-Q to remove traces of glutaraldehyde and media. The cell suspension was dried 6 min at 65 °C and subsequently mounted using VectaShield antifading agent. Fixed cells were imaged with a Nikon Eclipse upright microscope equipped with a 100× Nikon 1.45 PlanApo oil objective.

### Preparation of flow chambers and live imaging

For upright phase contrast imaging, flow chambers were prepared with a Soda Lime microscopy slide (1 × 24 × 75 mm, bottom) and a Soda Lime coverslip (0.17 × 24 × 40 mm, top, Fisher Scientific 11348503). Two strips of double-sided sticky tape were placed on the outer edges of the bottom glass and sealed. After assembly, flow chambers were incubated in a 20:80% N_2_:CO_2_ atmosphere for at least 2 h. A total of 100 µl of a Loki culture was sampled and transferred into a 2 ml Eppendorf tube filled with 20:80% N_2_:CO_2_. Subsequently, 50 µl of the culture were loaded into the flow chamber within an anoxic atmosphere. The flow chambers were sealed with Vaseline to avoid oxygen exposure in the phase contrast setup. The cells were incubated for at least 90 min before starting imaging experiments in a 20:80% N_2_:CO_2_ atmosphere. Time-lapse experiments were performed with an upright Nikon Eclipse microscope, equipped with a 100× Nikon 1.45 PlanApo oil objective and a phase contrast setup. Videos were acquired with a frame rate of 2 s and for a maximum of 30 min before placing the slide again into an anoxic chamber. For poly-l-lysine experiments, the bottom slide was coated with different concentrations of poly-l-lysine for 10 min, washed extensively with Milli-Q H_2_O and dried for 2 h at 60 °C. Flow chambers were assembled and imaged as described above. For inverted DIC microscopy, flow chambers were prepared as described above, but the bottom glass was exchanged with a thinner Soda Lime coverslip (0.17 × 24 × 50 mm, Fisher Scientific 12303148). DIC experiments were performed with a Nikon Ti2-E microscope, equipped with a 100× Nikon 1.45 PlanApo oil objective. The imaging slide was mounted with a custom-printed 3D slide holder and a custom slide holder stage. Next, a gas chamber was placed on top, enabling environmental control of the atmosphere surrounding the slide. The temperature was kept at 22 °C, and the atmosphere of the gas chamber was kept at 15:85% N_2_:CO_2_ by flowing in the gases at a flow rate of 13 l h^−1^ using an ibidi gas mixer. Oxygen levels were checked with Anaerostrips placed next to the flow chamber. The sealing with Vaseline minimized evaporation of the liquid over the course of the imaging experiment. DIC experiments were recorded with frame rates of 5–60 s, depending on the number of positions imaged and for a maximum of 6 h.

For live imaging experiments with fluorescently labelled DNA, 1 drop of NucBlue was added to 1 ml of culture before loading the flow chamber. Images were recorded every 60 s, with 20 ms exposure time and 1% power of the 405 nm Lumencor LED light source. For live fluorescence imaging of Lokiactin, we diluted the FastAct stock solution 1:1,000 directly with the L. ossiferum culture before loading the flow chamber, followed by incubation for at least 3 h. Images were recorded every 10–30 s, using a 50 ms exposure time and 25% power of the 640 nm Lumencor LED light source. Conditions for the experiments with SiR-Actin and FastAct_X were the same, and likewise for experiments with FastAct and Swin A except for the additional incubation with 250 ng ml^−1^ Swin A.

### Actin inhibitor experiments

Initial experiments of actin inhibitors were performed at 25 µg ml^−1^, 12.5 µg ml^−1^ or 5 µg ml^−1^ final concentrations (Supplementary Table 1). For these experiments, we used a single culture (24 days, 1.73 × 10^7^ L. ossiferum per ml and 58% relative abundance) and tested the effect of seven inhibitors each on three subsequent days, to ensure that inhibitor effects are not affected by changes in the cell cycle. We used concentrations higher than what is recommended for eukaryotic cells to compensate for potentially lower, hampered affinities for Lokiactin. Subsequent titration experiments were performed with inhibitors that had affected the morphology and protrusions of L. ossiferum in the initial screen. For this, we performed a serial dilution of the inhibitors (stock solutions either 1 mg ml^−1^ or 2.5 mg ml^−1^) in DMSO to reach concentrations from 250 to 0.25 µg ml^−1^ or 100 to 0.1 µg ml^−1^. We then added 1 µl from each inhibitor dilution to 199 µl of L. ossiferum culture in an N_2_:CO_2_ atmosphere. These titrations were performed with a single culture (25–28 days, 4.21 × 10^6^ L. ossiferum per ml, 22.57% relative abundance) for Chaetoglobosin A and Pseudofuscochalasin A. For Aspochalasin D and Swin A, titrations were performed with two L. ossiferum cultures (27 days old and 35 days old, 3.1 × 10^6^ and 4.3 × 10^6^ L. ossiferum per ml, 20% and 22% relative abundance). More details about the used actin inhibitors are provided in Supplementary Table 1.

### Image analysis

Image analysis in this study was performed with FIJI^61^. All plots have been created with Jupyter Notebook and all box plots indicate the median using the central line within the box, and the lower and upper boundaries of the box represent the first (Q1) and third (Q3) quartiles, respectively. Whiskers extend to the most extreme data points within 1.5 × interquartile range of Q1 and Q3. Individual points beyond the whiskers are shown as outliers. For colour coding the protrusion growth shown in Extended Data Fig. 1h, the time-lapse was first stabilized targeting the cell body, smoothed with a walking average of four frames and then subsampled every 30th frame. The resulting video was colour-coded using the Temporal-Color Code plugin and the mpl-magma lookup table. To measure the rates of protrusion growth and shrinking, regions of interest with single L. ossiferum cells were selected from videos and stabilized using the cell body as a matching template. Then, a line was drawn along the protrusion of interest, and a kymograph was created using the reslice command. The rate was determined by measuring the distance travelled over time.

### Measuring protrusion dimensions and the change of length

To measure the length of protrusions over time, single cells were selected, and the total length of all protrusions measured with the segmented line tool every 1 min or 5 min for a total of 15 min (phase contrast) or 60 min (differential interference contrast). The change in length (∆*L*) was calculated by $\Delta L=\frac{{L}_{0}}{{L}_{{i}}}\ast 100$, where *L*_0_ is the total length of the first time point and *L*_*i*_ the total length in subsequent frames. The diameter of protrusions and the cell body of cells was determined by extracting intensity line profiles from a line with line width = 5 and extracting the full width at half maximum from a Gaussian fit to the line profiles of either protrusions or cell bodies.

### Tracking cell migration

To quantify cell migration, we selected mobile cells and analysed the regions of interest. Videos were inverted, their contrast normalized for analysis, and cell bodies tracked with TrackMate^62^. Trajectories were built using the LAP tracker, allowing a linking maximum distance of 1 µm, a gap-closing max distance of 2 µm and a frame gap of a maximum of five frames. The resulting trajectories were manually curated, to avoid faulty linking due to focus shifts or floating of other, unbound cells into the field of view. Velocities, confinement ratio, track durations and displacements were recovered from corresponding .csv and .xml files. To determine the impact of protrusions on the migration direction, cells were manually segmented and used to train a pixel classification model in ilastik for automated detection of cell bodies and protrusion tips. Cell body trajectories were extracted with the TrackMate plug-in in Fiji. The movement direction was defined by the displacement vector between consecutive cell body centroids. For each frame, protrusion vectors were drawn from the cell centroid to each tip, and their alignment with the movement vector was calculated as the cosine of the included angle. Two metrics were derived: (1) the mean alignment, corresponding to the average cosine across all protrusions, and (2) the weighted alignment, given by the sum of cosines weighted by protrusion lengths (tip to centroid distances) and normalized by total protrusion length. Both indices range from −1 (protrusions opposing movement) to +1 (protrusions aligned with movement).

### Quantifying the effect of actin inhibitors on L. ossiferum

To assess the initial effect of inhibitors, we quantified the number of L. ossiferum cells with protrusions adhered to the glass slide after 90 min of incubation. For an in-depth analysis, the number of protrusions of each L. ossiferum cell in the presence of different concentrations of Swin A was counted, and we measured protrusion change length rates by kymography, as described above. To quantify the intensity of FastAct in the absence and presence of Swin A, we extracted line intensity profiles of the FastAct channel along the cell body or the protrusion with ImageJ. For each line profile, we estimated background fluorescence from the first five data points and subtracted it them from the entire profile, setting negative values to zero. The background-corrected signal was then integrated along the cell axis using trapezoidal integration. To account for differences in cell length, the integrated intensity was normalized by the profile length, yielding the mean fluorescence intensity per μm.

### Culture preparation for Co-IP experiments

For Co-IP, we selected three large-scale cultures (500 ml each) in stationary phase (1 × 47 days and 2 × 40 days old) at densities of 1.8–3.5 × 10^6^ L. ossiferum per ml and relative abundances above 20%. For fixation, formaldehyde was added to the cultures at a final concentration of 0.25% and incubated for 20 min with gentle agitation. Crosslinking was quenched by adding glycine to a final concentration of 250 mM for 5 min, followed by centrifugation and two washes with base medium. For immunoprecipitation, 10 µg of antibody was coupled using the Pierce Crosslink Magnetic IP/Co-IP Kit (Thermo Fisher), including crosslinking with DSS according to the manufacturer’s protocol. Cells were lysed in lysis buffer containing 50 mM Tris-HCl (pH 7.4), 150 mM NaCl, 10 mM MgCl_2_, 1 mM EDTA, 0.1% IGEPAL CA-630, and a protease inhibitor tablet. Lysates were sonicated (1 s on and 3 s off, 20% amplitude, 2 min) and centrifuged. Protein concentration was measured and 200 µg of protein from the supernatant of each culture incubated overnight with Lokiactin-antibody-coupled beads and IgG-coupled beads (Abcam) as a negative control. Beads were washed twice with a detergent-free buffer (20 mM Tris-HCl pH 7.5, 75 mM NaCl).

#### Sample preparation for mass spectrometry analysis of Co-IP experiments

The beads were resuspended in 30 µl 2 M urea and 50 mM ammonium bicarbonate. The beads were digested with 150 ng LysC (mass spectrometry grade, FUJIFILM Wako chemicals) at room temperature for 90 min. The supernatant was transferred to a new tube. The beads were rinsed with 30 µl 50 mM ammonium bicarbonate and combined with the supernatant. Disulfide bonds were reduced with 2.4 µl of 250 mM dithiothreitol (DTT) for 30 min at room temperature before adding 2.4 µl of 500 mM iodoacetamide and incubating for 30 min at room temperature in the dark. The remaining iodoacetamide was quenched with 1.2 µl of 250 mM DTT for 10 min. Proteins were digested with 150 ng trypsin (Trypsin Gold, Promega) in 1.5 µl 50 mM ammonium bicarbonate overnight. The digest was stopped by adding trifluoroacetic acid (TFA) to a final concentration of 0.5%, and the peptides were desalted using C18 Stagetips^63^.

#### Liquid chromatography-mass spectrometry analysis of Co-IP experiments

Liquid chromatography-mass spectrometry analysis was performed on a Vanquish Neo UHPLC system (Thermo Scientific) coupled to a timsTOF HT (Bruker). The system was equipped with a CaptiveSpray ion source (Bruker) and a column oven (Sonation). Peptides were loaded onto a trap column (PepMap Neo C18 5 mm × 300 µm, 5 μm particle size, Thermo Scientific) using 0.1% TFA as mobile phase, and separated on an analytical column Aurora Ultimate XT C18, 25 cm × 75 µm, 1.7 µm particle size, IonOpticks, applying a linear gradient starting with a mobile phase of 98% solvent A (0.1% FA) and 2% solvent B (80% acetonitrile, 0.08% FA), increasing to 35% solvent B over 60 min at a flow rate of 300 nl min^−1^. The analytical column was heated to 50 °C. The mass spectrometer was operated in data-dependent acquisition (DDA) parallel accumulation serial fragmentation (PASEF) mode. MS2 data were acquired with 10 PASEF scans per duty cycle. The ion mobility range was set to 0.64–1.42 V s cm^−1^, and the accumulation and ramp time was set to 100 m. Precursor ions were selected for fragmentation using an isolation window of 2*m*/*z* for *m*/*z* < 700 and 3*m*/*z* for *m*/*z* > 700. The precursor intensity threshold was set to 2,500, precursor repetitions were enabled with a target intensity of 20,000, and active exclusion was enabled with a 0.4 min release delay. Singly charged precursor ions were excluded using a polygonal filter applied in *m*/*z*-ion mobility space. TIMS elution voltages were calibrated linearly to obtain the reduced ion mobility coefficients (1/K0) using three Agilent ESI-L Tuning Mix ions (*m*/*z* 622, 922 and 1,222). Collision energy for fragmentation was scaled linearly with precursor mobility (1/K0), ranging from 20 eV (at 1/K0 = 0.6 V s cm^−1^) to 59 eV at (at 1/K0 = 1.6 V s cm^−1^).

#### Mass spectrometry data analysis of Co-IP experiments

Mass spectrometry raw data were analysed with FragPipe (23.1), using MSFragger (4.3)^64^, IonQuant (1.11.12)^65^ and Philosopher (5.1.2)^66^. The default FragPipe workflow for label-free quantification (LFQ-MBR) was used, except ‘Normalize intensity across runs’ was turned off. Cleavage specificity was set to Trypsin/P, with two missed cleavages allowed. The protein FDR was set to 1%. Carbamidomethyl was used as fixed cysteine modification; methionine oxidation and protein N-terminal acetylation were specified as variable modifications. MS2 spectra were searched against the *Ca*. L. ossiferum database with 5,119 sequences, concatenated with a database of 379 common laboratory contaminants (release 2025_01, https://github.com/maxperutzlabs-ms/perutz-ms-contaminants). Computational analysis was performed using Python and the in-house developed Python library MsReport (0.0.30) (https://doi.org/10.5281/zenodo.15309090)^67^. Only non-contaminant proteins identified with a minimum of two peptides and quantified in at least two replicates of one condition were considered for further analysis. LFQ protein intensities were log_2_-transformed and normalized across samples using the ModeNormalizer from MsReport. The ModeNormalizer method involves calculating log_2_ protein ratios for all pairs of samples and determining normalization factors based on the modes of all ratio distributions. Missing values were imputed by drawing random values from a normal distribution. *∑* and *µ* of this distribution were calculated per sample from the standard deviation and median of the observed log_2_ protein intensities (*μ* = median sample LFQ intensity – 1.8 standard deviations of the sample LFQ intensities, *σ* = 0.3 × standard deviation of the sample LFQ intensities). iBAQ intensities were calculated by dividing protein intensities by the number of theoretically observable tryptic peptides between 6 and 30 amino acids. To estimate the relative protein abundances within each sample, the iBAQ intensities were normalized by dividing them by the total sum of iBAQ intensities for all proteins in the sample. Statistical analysis was performed using the linear models for microarray analysis (limma) v.3.54.2 72 package in R. Moderated *t*-statistics were calculated using the limma-trend method^68^, and multiple testing correction was applied using the Benjamini–Hochberg method. The Python library XlsxReport (0.1.1; https://doi.org/10.5281/zenodo.15129818)^69^ was used to create formatted Excel files summarizing the results of the proteomics experiments.

#### Sample preparation for L. ossiferum proteome

To quantify expression levels of proteins in L. ossiferum, we selected three 500 ml (large scale), 56 days old cultures in stationary phase at densities of 1.1–2.4 × 10^6^ L. ossiferum per ml and relative abundances above 30%. Each culture was split into 5 × 50 ml flasks as technical replicates and subsequently centrifuged to harvest cell pellets. The samples were then processed with the iST kit (PreOmics) following the vendor’s protocol with slight adaptation. A total of 100 μl LYSE buffer (PreOmics) was added to the cell pellets and incubated in a shaker at 1,000 rpm for 10 min at 95 °C. The lysates were then sonicated in Biorupter for 5 cycles 30 s/30 s at high energy and subsequently heated in a shaker at 1,000 rpm for 10 min at 95 °C. Glass beads (30 µl) were added to the lysates, followed by sonication in the Biorupter with the same settings as before. The samples were then centrifuged at 14,000*g* for 5 min, and 50 µl of supernatant was transferred to new 0.2 ml tubes. Next, 50 μl DIGEST enzyme (PreOmics) resuspended in 210 μl RESUSPEND (PreOmics) were added and incubated for 3 h at 37 °C. Subsequently, digests were transferred to the cartridge and stopped by adding 100 μl of STOP solution (PreOmics) and peptides trapped on the iST Cartridges by spinning for 3 min at 2,250*g*. Two consecutive washes with WASH1 and WASH2 solutions (PreOmics, 200 μl each) were performed by spinning for 2 min at 2,250*g* followed by elution of peptides into 0.6 ml tubes with 2 × 60 μl ELUTE (PreOmics) by a final spin at 2,250*g*. Eluates were reduced in a vacuum concentrator at 45 °C. Dried peptides were taken up in 20 μl LOAD buffer (PreOmics).

#### Liquid chromatography-mass spectrometry analysis of proteome samples

Liquid chromatography-mass spectrometry analysis was performed on a Vanquish Neo UHPLC system (Thermo Scientific) coupled to an Orbitrap Exploris 480 mass spectrometer (Thermo Scientific). The system was equipped with an FAIMS Pro (Thermo Scientific), a Nanospray Flex ion source (Thermo Scientific), coated emitter tips (PepSep, MSWil) and a Butterfly Portfolio Heater (Phoenix S&T). Peptides were loaded onto a trap column (PepMap Neo C18 5 mm × 300 µm, 5 μm particle size, Thermo Scientific) using 0.1% TFA as mobile phase, and separated on an analytical column (Acclaim PepMap 100 C18 HPLC Column, 50 cm × 75 µm, 2 μm particle size, Thermo Scientific), applying a linear gradient starting with a mobile phase of 98% solvent A (0.1% FA) and 2% solvent B (80% acetonitrile, 0.08% FA), increasing to 35% solvent B over 60 min at a flow rate of 230 nl min^−1^. The analytical column was heated to 30 °C. The mass spectrometer was operated in data-independent acquisition (DIA) mode with FAIMS compensation voltage (CV) set to −45, with a 3 s cycle time. Survey scans were acquired from 350 to 1,200*m*/*z*, normalized AGC target of 300%, resolution of 60,000. Per cycle, 45 MS2 spectra were acquired across a mass range of 349.5–1,200.5*m*/*z* using variable isolation windows (9-248*m*/*z*) with 1*m*/*z* overlap between windows. Selected ions were analysed using automatic maximum injection time, normalized AGC target of 1,000% and resolution of 30,000 after HCD fragmentation with normalized collision energy of 30%.

#### Data analysis of proteomic samples

Mass spectrometry raw data were processed with Spectronaut (18.7, Biognosys). The library-free DirectDIA+ workflow was used for analysis of the raw files, the Lokiarchaeum_ossiferum_B-35 protein database (5119 sequences), concatenated with a database of 379 common laboratory contaminants (release 2023_03, https://github.com/maxperutzlabs-ms/perutz-ms-contaminants). The cleavage specificity was set to full trypsin specificity (Trypsin/P), with two missed cleavages allowed. Carbamidomethyl was used as fixed cysteine modification; methionine oxidation and protein N-terminal acetylation were specified as variable modifications. Cross-run normalization was disabled, and all other settings were used at their default values. Computational analysis was performed using Python and the previously developed Python library MsReport (0.0.24, https://doi.org/10.5281/zenodo.15309090 (ref. ^67^)) by the VBC Mass Spec Facility. Only non-contaminant proteins identified with a minimum of two peptides and quantified in at least two replicates of one condition were considered for further analysis. LFQ protein intensities were log_2_-transformed and normalized across samples using the ModeNormalizer from MsReport. Intensity less than 1,024 was set to NA. The ModeNormalizer method involves calculating log_2_ protein ratios for all pairs of samples and determining normalization factors based on the modes of all ratio distributions. Missing values were imputed by drawing random values from a normal distribution (*µ* = log_2_(1,024), *∑* = 0.75, seed = 64). iBAQ intensities were calculated by dividing protein intensities by the number of theoretically observable tryptic peptides between 6 and 30 amino acids. To estimate the relative protein abundances within each sample, the iBAQ intensities were normalized by dividing them by the total sum of iBAQ intensities for all proteins in the sample. Statistical analysis was performed using the linear models for microarray analysis (limma) v.3.54.2 package in R^68^. Moderated *t*-statistics were calculated using the limma-trend method considering the replicate as a batch variable, and multiple testing correction was applied using the Benjamini–Hochberg method. The Python library XlsxReport (0.1.1, https://doi.org/10.5281/zenodo.15129818)^69^ was used to create formatted Excel files summarizing the results of the proteomics experiments.

#### Gelsolin domain alignment and phylogenetic tree

Reference sequences for gelsolin-like homologues, including gelsolin, villins and scinderin^26,70–72^, were initially retrieved from the National Center for Biotechnology Information (NCBI) database. Further phylogenetic analysis was restricted to the first gelsolin homology domain (G1 core domain, encompassing cofilin, actin-depolymerizing, severin and scinderin domains) of all sequences analysed. The G1 domains of L. ossiferum gelsolins were identified and extracted based on InterPro annotations (Supplementary Table 3). Then, the extracted domains were clustered using MMseqs2 with a minimum sequence identity threshold of 0.5 and a coverage threshold of 0.8 (–min-seq-id 0.5 -c 0.8). Reference G1 domains were subsequently added to this clustered dataset, resulting in a final working database of 174 representative sequences from 123 organisms.

### Structure-based phylogeny

Protein structure files (.pdb) for all representative sequences were obtained from public databases or predicted de novo using HT-ColabFold^73–76^. The structural models were truncated based on the corresponding G1 domains. A structure-based phylogenetic tree was subsequently reconstructed using the recently reported 3DiPhy approach, which leverages a general substitution matrix optimized for structural phylogenetics to compute maximum-likelihood trees directly from 3D structural alignments^77^. The resulting tree was inferred with IQ-TREE 3.1.0 (ref. ^78^).

### Reporting summary

Further information on research design is available in the Nature Portfolio Reporting Summary linked to this article.

## Online content

Any methods, additional references, Nature Portfolio reporting summaries, source data, extended data, supplementary information, acknowledgements, peer review information; details of author contributions and competing interests; and statements of data and code availability are available at https://doi.org/10.1038/s41586-026-11063-9.

## Supplementary information

Supplementary Information This file contains Supplementary Discussion and Supplementary Figs. 1 and 2.

Reporting Summary

Supplementary Table 1 Actin inhibitors. Details regarding all the actin inhibitors used in this study, including supplier, references, their measured IC50 and the concentrations used throughout this paper.

Supplementary Table 2 Proteome and Co-IP results. Raw data from proteome and Co-IP experiments. Furthermore, the table contains information about protein identifiers used in the study and the corresponding NCBI accession numbers.

Supplementary Table 3 Domain identifiers for Gelsolin phylogeny. Identifiers of the key domains used to build the phylogenetic tree in Supplementary Fig. 2.

Supplementary Video 1 *Ca.* L. ossiferum in upright PC and inverted DIC microscopy. Representative full field of view from upright phase contrast (PC, left) and inverted differential interference contrast (DIC, right) microscopy experiments on adherent L. ossiferum cells. For further analysis, single cells were selected and processed. Experiments are shown at 60 s per frame.

Supplementary Video 2 L. ossiferum cells adhering to glass substrate. Representative phase contrast (PC) experiments of floating L. ossiferum cells adhering to the bottom glass using their protrusions. After landing, the protrusions extend to anchor the cells at the glass surface and explore their local environment. The video on the right corresponds to Fig. 1f, experiments were recorded with 4 s per frame (left) or 2 s per frame (right).

Supplementary Video 3 Protrusions of L. ossiferum extend and retract dynamically. Representative PC (left and centre) and DIC (right) experiments of L. ossiferum cells constantly rearranging, growing and retracting protrusions. The videos correspond to Extended Data Fig. 1h (left) and 1g (right). Experiments were recorded with 2 s per frame (PC) or 60 s per frame (DIC).

Supplementary Video 4 L. ossiferum substantially rearranges its cell morphology. L. ossiferum adopts different morphologies and dynamically changes its shape. Representative experiments show rearranging protrusions, shapeshifting cell bodies and two cell body-like structures merging. Experiments were recorded with 2–5 s per frame and correspond to Extended Data Fig. 1i,j.

Supplementary Video 5 L. ossiferum chromosome adapts to different shapes. Representative DIC experiments of L. ossiferum (grey) and stained DNA (cyan, NucBlue). Two cell bodies in proximity, potentially post division (left). DNA signal in a stick shaped cell is dynamically redistributed on a minute time scale (right). Experiments were recorded with 60 s per frame and correspond to Extended Data Fig. 1k.

Supplementary Video 6 L. ossiferum cells use different modes of motion. Representative PC and DIC experiments of migrating L. ossiferum cells. The cell body is translocated either by forward pulling by protrusions at the front (left) or by a mix of pulling and gliding (right). Experiments were recorded with 2 s per frame (PC) or 60 s per frame (DIC). The bottom left video corresponds to Fig. 2e.

Supplementary Video 7 L. ossiferum cells gliding along glass. Representative PC (left) and DIC (right) experiments of gliding L. ossiferum. In both examples, the cells extend a leading protrusion guiding the direction of migration. Experiments were recorded with 2 s per frame (PC) or 10 s per frame (DIC) and correspond to Fig. 2a,f.

Supplementary Video 8 Image segmentation of migrating L. ossiferum cells. Representative experiments of the segmentation result using Illastik. An overlay of the raw DIC video (grey), segmentation of the whole cell (blue), the cell body (red), the tips of the protrusion (green) and the vector of cell body displacement (yellow arrow) is shown. The videos were stabilized using the cell body as target. The length of the arrow is correlated with the length of displacement. The examples for pulling (left) and gliding (right) were recorded with 30 s per frame (left) or 10 s per frame (right), respectively. Experiments correspond to Fig. 2e,f and Extended Data Fig. 2h.

Supplementary Video 9 Live staining of Lokiactin using FastAct. Representative experiments of L. ossiferum cells stained with FastAct and NucBlue (left) or FastAct only (right). Experiments were recorded with 30 s per frame or 10 s per frame (bottom right) and correspond to Fig. 3c.

Supplementary Video 10 Effect of Swinholide A on L. ossiferum. Representative experiments ofL. ossiferum with increasing Swinholide A concentrations (0.5/5/50/500 ng ml). With concentrations higher than 50 ng ml^−1^, cells start to lose control over protrusions, which become increasingly curved and wavy. Experiments were recorded with 2 s per frame and correspond to Extended Data Fig. 3g.

Supplementary Video 11 M. peptidophilum exhibits similar dynamics. Representative PC experiments of adherent M. peptidophilum cells. Their protrusions are thicker and cell bodies larger than observed with L. ossiferum, but the dynamic rearrangements of their morphology are comparable. Experiments were recorded with 2 s per frame or 5  s per frame and correspond to Fig. 4a.

Supplementary Video 12 Effect of Swinholide A on M. peptidophilum cells. Representative experiments of M. peptidophilum with increasing Swinholide A concentrations (5/50/500 ng ml^−1^). At concentrations higher than 50 ng ml^−1^, cells start to lose control over their protrusions. Experiments were recorded with 5 s per frame and correspond to Extended Data Fig. 4a.

Peer Review File

## Source data

Source Data Fig. 1

Source Data Fig. 2

Source Data Fig. 3

Source Data Fig. 4

Source Data Extended Data Fig. 1

Source Data Extended Data Fig. 2

Source Data Extended Data Fig. 3

Source Data Extended Data Fig. 4

## Data Availability

The plots in this manuscript were created using Jupyter Notebook; the source data for all figures have been provided in data source tables. Microscopy raw data for supplementary videos, the Jupyter notebooks to generate the plots and the raw files for the structural phylogenetic analysis are available at https://doi.org/10.6084/m9.figshare.30676367. The remaining raw microscopy data generated for this manuscript are available from https://www.ebi.ac.uk/biostudies/bioimages/studies/S-BIAD3928. The mass spectrometry proteomics data from the Co-IP experiments have been deposited to the ProteomeXchange Consortium via the PRIDE partner repository^79^ and are available at https://www.ebi.ac.uk/pride/archive/projects/PXD068820. The mass spectrometry proteomics data of the proteome analysis is available at https://www.ebi.ac.uk/pride/archive/projects/PXD074767. Source data are provided with this paper.
