# Supplementary material for: Dynamic protrusions mediate crawling motility in Asgard archaea
Source: Nature. 2026 Sep 30;658(8138):1218–25. doi: 10.1038/s41586-026-11063-9 (PMC13716822; doi:10.1038/s41586-026-11063-9)
Supplement: Supplementary file 1 [file 41586_2026_11063_MOESM18_ESM.pdf]

nature portfolio

Peer Review File

Dynamic protrusions mediate crawling motility in Asgard Archaea

Corresponding Author: Professor Christa Schleper

Any redactions in this file are there to maintain patient confidentiality, the confidentiality of unpublished data, or to remove third-party material.

This file contains all reviewer reports in order by version, followed by all author rebuttals in order by version.

Version 0:

Reviewer comments:

Referee #1

(Remarks to the Author)
In this manuscript, Radler and colleagues achieve the first live-cell imaging of Asgard archaea, which are anaerobic and notoriously difficult to cultivate, and which are the proposed archaeal ancestors of eukaryotes. They provide novel insights into the behavior and cell biology of this unique group of microorganisms. In contrast to the slow growth rate of Asgard archaea, the authors found that these organisms rapidly and dynamically change their cell shape: constantly extending, retracting, and rearranging their membrane protrusions and cell body. Further, using automated cell tracking, the authors show that Asgard archaea are motile and provide evidence that the microbes can use their protrusions to pull their cell body across a surface and "crawl" in a manner reminiscent of eukaryotic motility using pseudopodia. The authors find convincing evidence that these observed dynamics are dependent upon the actin filaments found in Asgard archaea membrane protrusions, whose function is highly debated in the field, by showing that the presence of actin inhibitors in the media fully arrests both cell shape changes and movement. The authors show that these groundbreaking findings are consistent amongst different clades of Asgard archaea - both the Lokiarchaea and the Hodarchaea, the Asgard lineage most closely related to eukaryotes, indicating that these novel dynamics are a conserved hallmark of Asgard archaea and that they may have played a role in facilitating eukaryogenesis. Lastly, they propose a potential mechanism for the regulation of actin dynamics in Asgard archaea based on gelsolins and profilins. While the paper's conclusions are mostly supported by the data, more work is required to show that this crawling phenotype, which has not been observed before, is physiologically relevant in Asgard archaea (i.e. does not depend on the imaging conditions).

Major comments:
- While cells clearly seem alive in all the live-cell imaging assays shown, membrane protrusions dynamics may be the result of cellular stress (e.g. prolonged absence of physical interactions with bacterial symbionts due to differential adhesion to the glass substrate, leakiness of the imaging chamber leading to the presence of oxygen, etc). For instance, the authors fail to observe cell division events. While this may be due to the slow growth rate of Asgard archaea, it may also be due to the imaging conditions causing cellular stress. This could be ruled out by 1) the use of a redox-sensitive dye such as resazurin to indicate that the imaging conditions are, in fact, anaerobic, 2) a measurement of cell growth rates inside of the imaging chamber, 3) the observation of cell division events. This is a critical point since this behavior has not been observed previously.

- It is unclear what the authors mean by "long term imaging". Are cells continuously imaged for a prolonged period of time (weeks) inside of the imaging chamber, or simply sampled at longer time points after passaging a culture? This should be made clearer throughout the text and method sections. Related to this, it is unclear what data supports the authors' observations of a "two-week imaging experiment" as Fig. S1 reports the results from a "7-day imaging experiment". Also, it is unclear how imaging was done. The main text only describes a vaseline-sealed chamber, but the methods describe the flowing of gas mixes. Does this mean the authors poked a gas line needle through the Vaseline and made an exit hole on the opposite side to facilitate this gas flow? Or did they place the glass slide + double sided + coverslip into an additional chamber where they can control the atmosphere? Additionally, where specifically were the anaerostrips placed to monitor the presence of oxygen? Why 22C vs. the standard growth temp of 20C? Was this the temperature used for both L. ossiferum and M. peptidophilum? Why 15:85 N_2:CO_2 and not the standard 20:80 N_2:CO_2? Won't this alter the pH?

- Page 5 (and throughout the manuscript): In describing motility patterns and speeds in Asgard cells, the authors compare them with the crawling speed of eukaryotes and the swimming speed of bacteria, archaea and flagellates. This draws a rather misleading or incomplete picture as different, unrelated structures mediate swimming in archaea (archaealla), bacteria (flagella) and flagellates (cilia). Therefore, swimming speeds should be described separately. Moreover, the authors fail to compare Asgard cell motility with type IV pilus-mediated twitching motility in archaea (in the order of 18 µm/min; see Charles-Orszag et al. 2024, DOI 10.1038/s41467-024-49101-7) and bacteria (in the order of 60 µm/min; see Merz et al., 2000, DOI 10.1038/35024105). This should be added to the result section and discussed further.

- The authors state that Asgard cells move around by pulling and gliding. However, they do not explain why they rule out pushing. Also, it is mentioned that gliding may be due to actin treadmilling. However, this could also be explained by simultaneous actin polymerization in the front and active actin depolymerization in the rear of the migrating cell body. Lastly, movies of motile cells clearly show that cell bodies experience two distinct modes of displacements: slow displacements over short distances that seem to be mediated by gliding, and larger, sudden displacements that appear to be mediated by the rapid buckling and surface detachment of membrane protrusions located in the rear of the cell (relative to the direction of cell migration) followed by their reabsorption back into the cell body. In other words, it looks as though membrane protrusions act like dynamically adhesive springs under tension. All these should be more carefully described and reinterpreted.

- Fig. 4: Authors should also have plots similar to Fig. 3E-H to show that/if these dynamics were quantified. They don't appear to have quantified the number of M. petidophilum per FOV, protrusion per cell, protrusion growth rate, or % of mobile cells in the presence of Swin A. The latter three are important to quantify if they want to make the claim that SwinA arrested the rearrangement of cell morphology and migration.

- Page 10: The part addressing the potential role of gelsolins and profilins may not need to be an entire section in the main text. It could be moved to the supplement and/or the Co-IP experiments could be introduced briefly at the end of section 3 to provide insight into what proteins might be regulating the lokiactin cytoskeleton which appears to be driving the locomotion in these cells. This last section feels a little underdeveloped and out of place, it would make more sense to present it in the context of section 3 after it is shown that actin appears to be involved in the movement of the Loki cells.

- In the discussion, the authors claim that examples of cell motility in the absence of proteins such as actin polymerases or myosin have been described before. However, some of the three references cited do not actually support this. Ref. 55 actually reports actin-independent cell motility based on MSP (major sperm protein). Ref. 56 reports the mechanisms of myosin-independent contraction of actin rings, not myosin-independent actin-based motility. Please revise accordingly.

Minor comments:
- Throughout the manuscript: it is "poly-L-lysine" not "lysin"
- Page 1: It appears that the authors based their measurement of cellular protrusions diameter on FWHM values obtained through DIC imaging (Fig. 1D). Could the authors clarify why they did not use values obtained through electron microscopy instead? Even though SEM may lead to overly dehydrated cells and affect the aspect of membrane protrusions, these protrusions have been observed before through cryoEM imaging which likely represent cells in their near-native state (Rodrigues-Oliveira et al., 2023; Imachi et al., 2020; Imachi et al., 2025). Could the authors describe how their DIC measurements compare with previous cryoEM data? This is important as it impacts their estimate of increase in cell surface area. Also, please show representative SEM data that were used to measure protrusions diameter.
- Page 8: Similarly, please indicate how membrane protrusion diameter was measured for M. peptidophilum cells.
- Could the authors indicate the percentage of L. ossiferum enrichment cultures that were chosen for live-cell imaging in the main text? Similarly, please report the same value for M. peptidophilum as it is not reported in the methods section.
- Page 4: Replace "life staining" with "live staining".
- Page 4: No data in the main or supplementary figures seem to corroborate the observation that NucBlue-stained chromosomes undergo fusion. Please clarify.
- Fig. 1H: It would be visually easier to understand if the authors used more intermediate time points between 0 and 3 min, 3 and 6 min etc. If space is limiting, panel G could be removed as the timelapse clearly shows protrusions dynamics.
- Fig. 1I: The Y-axis can not read "protrusion growth rate" in the case of protrusion shrinkage and catastrophe rates unless these were represented as negative growth rates.
- Fig. 1K: Could the authors label the difference between top and bottom graphs? Alternatively, this could be described in the text.
- Fig. 1, legend B, C and D: Please indicate sample size for all three.
- Page 7: The rates of protrusion growth actually drop by ~50%, which is more than "slightly"
- Fig. 4, title: Replace with "actin-dependent" and add "and surface motility" or "crawling motility" to best describe the observations reported in the figure.
- Fig. 4H: The SwinA treatment does not seem to affect the number of protrusions in this example. Could the authors clarify?
- Page 7: SiR Actin and its FastAct derivatives are thought to specifically bind F-actin in eukaryotes and not monomeric actin. Based on previous cryo-EM images of L. ossiferum, the cell body does not appear to contain many actin filaments, especially compared to the protrusions. Why, then, do the author's think FastAct labels the central cell body so brightly? Please discuss.
- Page 7: Did the authors mean to say "low concentrations" here instead of high? Most of the actin inhibitors should work at low concentrations in eukaryotes.
- Page 7: The authors describe that the number of protrusions decrease upon treatment with SwinA. Since the length of protrusions remains constant when the cells are dynamically rearranging, is this loss of protrusions accompanied by an

increase in cell body diameter or an increase in length of the remaining protrusion?
- Page 7: Swin A works by sequestering actin dimers AND by severing existing F-actin. Since the fluorescent signal was not lost completely, does this suggest that the FastAct stain may not be specifically binding to F-actin and may also be binding to monomeric actin? This could explain why the cell body is so bright.
- Fig. 4D: Please indicate how many experiments were performed to measure the 31 protrusions.
- Page 10: Please remind the reader how many profilin and gelsolin homologs were known to be encoded in these genomes.
- Page 10: Only 8 complete genomes appear on GTDB. There are 8 genomes classified as complete and 7 genomes classified as a "chromosome" but that are allegedly not complete. But maybe this is the 15 the authors are referring to? Please clarify.
- Page 10: "myo-inositol-1-phosphate" refers to a molecule. What is the enzyme the authors are referring to?
- Fig.5, title: Replace with "Co-immuno"
- Discussion: It would be more exhaustive to also mention other known membrane reservoirs in eukaryotes such as caveoleae (Sinha et al. 2011, DOI 10.1016/j.cell.2010.12.031).
- Discussion: remove "e.g." in "as e.g. suggested"
- Discussion: replace by "will be THE subject of future work".
- Discussion: The authors propose that actin regulation may be calcium-dependent. Would it be possible to perform experiments using calcium ionophores to test the hypothesis?
- Discussion: Please discuss whether the last common Asgard ancestor is thought to have already possessed lokiactin, gelsolin, profilin, and enzymes involved in the synthesis of PIP-like headgroups.
- Discussion: Regarding current models for eukaryogenesis, please also cite the model by Krupovic et al., 2023 (DOI 10.1038/s41564-023-01378-y).
- Discussion: regarding "deep marine sediments", this is rather speculative. Why would the sediments need to be deep? Most eukaryogenesis models involve at least some oxygen which was not present in the deep ocean two billion years ago.

Referee #2

(Remarks to the Author)
I co-reviewed this manuscript with one of the reviewers who provided the listed reports.

Referee #3

(Remarks to the Author)
In their article "Dynamic protrusions mediate unique crawling motility in Asgard Archaea (Promethearchaeota)" Radler et al used live cell microscopy to investigate the potential of cell motility for the first cultivated members of the Asgard archaea. The authors provide stunning images/movie clips indicating that the investigated organisms can dynamically change cell shape and use their protrusions for active crawling motion powered by their actin cytoskeleton. Considering that a lineage within Asgard archaea is hypothesized to have given rise to the eukaryotic nuclear lineage about 2 billion years ago and that Asgard archaea encode various so called eukaryotic signature proteins, research shedding new light into the cell biology of members of this group is important: the authors employ among others advanced imaging techniques that suggest that cellular motility of extant representatives of the Asgard archaea resembles eukaryotic crawling motility while being unusual for prokaryotes.

The approach and data quality seem overall robust and reliable, with the caveat that I am not an expert in microscopy. I provide some minor comments regarding methods and data below.

Minor comments:
- consider to also acknowledge the alternative formal classification based on SecCode: Asgardarchaeota
- you mention: "the measured migration velocities of cell bodies were remarkably similar to the observed protrusion growth rates, suggesting a connection between these two."
I am wondering what the causal link would be and whether this could just be coincidence?
- how do you define "in front" of the body? Is there a way to determine the front in the absence of movement towards something?
- you mention "We found homologues of three enzymes involved in PIP headgroup synthesis". Where did you find those: i.e. encoded in the genome, proteome, in conncectin with the actins…? Which are these?
- The section on "Gelsolins and profilins as putative regulators of Lokiactin polymerization" could be presented a bit more clearly: i.e. which proteins were detected in the proteome, and which did you look at because they are encoded in the genome: "A phylogenetic analysis of L. ossiferum gelsolins…" Do you mean all gelsolins encoded in the genome? Or all gelsolins detected? In line with this, in Fig 5E: can you provide accession numbers to the previously characterised gelsolins?
- While I agree with the authors that "the described plasticity and motility might have played an important role in eukaryogenesis…", the authors could perhaps mention the caveat that these analyses are based on two extant model systems which may have different cell biological features than the archaeal ancestor of eukaryotes.

METHODS
- It would be great if you can provide more details about the media and cultivation conditions for the enrichment cultures here (instead of referring to previous articles). Likewise, it would be helpful if you can provide additional information about the

enrichments used for the various downstream experiments (some of these aspects are mentioned sometimes but not consistently): What growth phase were cultures in? How many replicates did you use? How do the enrichments change over time, i.e. regarding the percentage of Asgardarchaeota relative to other organisms or are these pure enrichments? Where are the inocula coming from: e.g. do you have a master enrichment, do cultures continuously evolve, or do you have cryostocks, etc? If the latter, can you provide a protocol? How much enrichment was needed for the various analyses. See also some concrete examples below:
- can you expand a bit on how the actin inhibitors were tested in the methods section “Inhibtor experiments”), including inhibitors and concentrations tested, growth state of culture, replicates etc? Or, what is the difference between these two sections; “Effect of actin inhibitors” and “Inhibitor experiments”. Should these sections be merged?
- regarding the Co-Immunoprecipitation methods section: can you add some information about the growth phase of the culture used for this analysis?
- please also add information about the cultures and replicates subjected to Liquid chromatography-mass spectrometry analysis and Mass Spectrometry data analysis
- Phylogenetic analyses: how were gelsolin sequences from Asgards selected? Did you test the more complex models of evolution such as C-series model that are not automatically tested by IQtree and perform H-like approximate likelihood? I would recommend adding more gelsolins from all Asgard archaea and assemble a comprehensive dataset for eukaryotic homologs (e.g. by identifying homologs using key protein domains) rather than just adding some of the homologs: specifically, I wonder whether the current phylogenetic analyses could be biased by LBA due to inclusion of only few long-branching Asgard gelsolins leading to misclassifications of the analysed Asgard gelsolins. It might also be easier to read the tree in supplementary Figure 5 if it was displayed vertically, eukaryotic and asgard homologs were clearly labelled and bootstraps shown. (in that case Fig 5b would not be needed). Please also provide information about the length of the alignment: is it long enough to resolve the relationships?
- Supplementary tables and movies:
I could not see the original file names of the supplementary tables and movies so it wasn’t entirely clear to me when they were cited in the manuscript: but this might just be due to the conversion of the files in the system. Perhaps references to the tables could be added to the method sections for clarification of some of the missing details. Are there legends to describe the Supplementary Tables somewhere (I could not find them)?
DATA AVAILABILITY
- it would recommend that you make all source and microscopy data available via a repository rather than via contact of the first author. Also, the sequence and alignment files and results from phylogenetic analyses as well as scripts should be made available.

Referee #4

(Remarks to the Author)
One fundamentally important question in evolutionary cell biology is how did the dynamic cytoskeleton of eukaryotic cells evolve? The acquisition of new modes of motility and contractility enabled cells to successfully hunt prey, divide and form multicellular clusters and tissues, for example. The recent exciting discoveries of archaeal actin along with a handful of actin regulators (e.g. profilin and gelsolin-like proteins) and identification of actin filaments in Archaeal cells raised the question of whether actin dynamics could drive the formation of dynamic protrusion and even motility in these organisms. Radler et al answer this with a resounding YES.
Live cell imaging of two different species of Archaea (L. ossiferum and M. peptidophilum) reveals a dynamic morphology, with a cell body that can change its shape and actively extend multiple thin, actin-filled protrusions that branch and retract. These can be visualized using the live cell stain FastAct, consistent with previous work identifying actin filaments in Archaeal extensions. A systematic screen of actin inhibitors showed that select cytochalasin derivatives or swinholide promote loss of protrusions and cell adherence. Interestingly, for both species the rate of protrusion extension matches the speed of the cell body, with the larger M. peptidophilum cells moving faster than the smaller L. ossiferum cells. Inhibition of actin dynamics results in a loss of protrusions, reduced motility and detachment, providing strong support for the conclusion that actin dynamics are critical for the observed motile activity. The analysis presented suggests that the cells move either by gliding or pulling from the tip of the protrusion, but not by pushing from the cell body. Co-IP using a Lokiactin antibody identified several actin regulators, profilins and gelsolin-like proteins, that have been shown in other studies to have polymerizing and severing activities in vitro, in the pellet. Together, these data reveal that Archaea can migrate and strongly suggest that they could do so via dynamic actin polymerization of elongated protrusions controlled by archaeal profilin and gelsolins.

The results here provide an exciting glimpse into the emergence and use of actin dynamics in early evolution. The work establishes methods for live cell imaging of Archaea, along with visualization and manipulation of the actin cytoskeleton, important technical advances that will propel future studies in the field and serve as a template for others investigating the cytoskeleton in evolutionarily divergent organisms. The motility is carefully described and the data showing long, thin protrusions are critical for it is consistent with actin dynamics playing a critical role in this unique mode of migration. The identification of actin regulators that co-precipitate with Lokiactin is interesting, but the work seems to be preliminary in the sense that a list of proteins is presented and the story ends there. This is not surprising given the challenges of working with a divergent cell type, but it leaves one with mostly speculation and not knowing what to make of it all. Thus, as with any significant advance, the findings prompt many questions that go beyond the scope of the present work. These include, but are not limited to, what are the identity and characteristics of the key players and the detailed molecular mechanisms responsible for actin dynamics, which proteins mediate substrate adhesion and how are they linked to actin filaments, how do the protrusions pull the cell body and/or do they mediate gliding, and how are the dynamics of Lokiactin controlled by the candidate regulators in vivo. The findings have set the stage for future work that will require continued development of

methods to analyze the proteins and cellular behavior that will surely provide the broader cytoskeletal and evolutionary cell biology fields with more intriguing and surprising discoveries.

SPECIFIC COMMENTS

1) The authors test a series of actin drugs and identify several that affect actin filament formation in L. ossiferum (Fig 3D, Supp Table 1). Many will be familiar with cytochalasins, but perhaps not the existence of so many variants, and may have only heard of swinholide A, but not know its mode of action. It would be helpful if the authors could provide a brief description of what is known about how these compounds disrupt actin polymers/polymerization.
If the structure of Lokiactin is similar enough to that of crenactin, can one predict if known interaction sites for swinholide A and cytochalasins (presuming their interaction with actin is the same as the widely use cytochalasins A and D) are the same as seen for eukaryotic actin?

2) Analysis of the motility of L. ossiferum shows different modes, including random and sometimes persistent. The authors comment that the persistent movement appears “as if aimed towards a signal”. This may be the case, but it is not unusual for migratory cells to have periods of persistent movement so this seems rather speculative.
Later, it is stated that M. peptidophilum cells “migrated along the glass randomly, most likely due to the lack of a target.” Again, the implication that motility is directed by a signal or target, while tempting, is not supported and without an analysis of the proportion of each type of motility the cells exhibit (both cell types) it is hard to say much about persistent motility, or lack thereof, here.
However, if the cells are actually moving with high persistence towards a signal, then what could it be? Is there any relationship between these persistent motile periods and a neighboring L. ossiferum or even a syntrophic partner?

3) Potential actin binding proteins are identified by a combination of proteomic analysis and co-immunoprecipitation using anti-Loki antibodies. One concern is that validation of the antibody is not provided - while the immunostaining strongly supports its specificity, anti-peptide antibody reactivity can be sensitive to context (e.g. works in western blots but not staining and vice versa) and its possible that the antibody could cross-react with other proteins in solution. More details about the validation should be provided.

4) The section describing the proteomics and co-IP (starting on the 2nd paragraph on page 10) is a bit confusing to follow. It starts off with “Our proteomic analysis confirmed the expression of gelsolins and profilins within the top 500 out of 3061 expressed proteins....”. At first read it is not clear how the authors arrive at expression levels from a proteomic analysis because none has been described at this point. Was this an RNA analysis or a proteomic analysis that looks at total cell proteins or something else? Please provide a more clear description of what was done here.

5) Mov S9 - FastAct treated cells
It appears as if protrusions interact with each other, adhering cells together - is that an accidental/coincidental interaction just seen is this movie or is it observed in other cases where two cells are nearby each other?

6) FastAct treatment decreases the protrusion extension rate rather significantly, from 1.5 µm/min to 0.7 µm/min (Fig 3b). Is there a corresponding slowing of the cell body speed?

7) Mammalian cells expressing the archaeal gelsolins 2D-Gel and 2D-Gel2 that have been treated with ionomycin to induce global increase of calcium results in a significant reduction in actin polymer (Akil et al, PNAS, 2020; Comm Biol, 2022). While its possible that ionomycin may not work with archaeal cells, have the authors tried applying it to cells or use any other manipulation that would alter calcium levels, to see if it might affect protrusion dynamics in any way?

8) Are actin filaments in protrusions are bundled or are they largely are single filaments based on the available structural information (e.g Rodrigues-Oliveira, Nature, 2022)?

MINOR POINTS

a) Fig 1e The imaging chambers illustrated here appear to be sealed at both ends with Vaseline but it appears that maintaining and anoxic atmosphere required flowing in a N_2:CO_2 mix. It might be useful to illustrate this in the lower diagram.

b) Fig 1i, third panel The description at the bottom is “Catastrophe” - using the descriptor “Catastrophe” may be confusing as this is typically associated with the rapid disassembly of microtubules
The y-axis for “Shrink” and Catastrophe are labeled “Protrusion growth rate (µm/min)”, shouldn’t they be retraction rates?

c) The concentrations of the inhibitors tested here are all in ng/mL - while that does convey what was used, it is the standard to use µM.

d) Fig S3E, legend Indicate what NC is (presumably it means negative control). Is each dot an individual cell? Please provide overall N and n values for these experiments (not needed for each separate one, a range should be fine).

e) Fig S3I Cells treated with swinholide A were stained with FastAct and then the signal quantified and compared with untreated cells. It does appear that the signal is reduced, consistent with loss of actin filaments, but the treated cell body

looks to be larger than that of the control. Has cell size been corrected for here?

f) The number of replicates done for the co-IP/mass spec analysis is not clearly described anywhere. It appears that at least two or more were carried out and that should be described in the methods or Figure legend.

g) Discussion The authors mention here that red algae are an example of an organism that exhibits motility in spite of its lack of “a more complex migration regulation machinery”. This is an important point, it might also be worth mentioning Trichomonas, although it does have a larger complement of actin regulators (including formins), it lacks myosin yet still appears to able to move in an amoeboid fashion (Kusdian et al, Cell Microbiol, 2013).

h) Supp Table 2 presents raw proteomic data. The first column in sheet 1 lists protein designators but does not provide protein names. Is there a database where one can look up these proteins or is everything listed in the third sheet?
It is not so easy to know what the titles of every column or the numbers mean in some cases (sheet 1, for example).
The authors are encouraged to provide a front sheet that explains what is being presented in each sheet in the table.

Version 1:

Reviewer comments:

Referee #1

(Remarks to the Author)
The authors addressed our concerns carefully and improved the manuscript significantly. The revised version is clearer and overall much stronger than the original submission. We appreciate the thorough efforts made by the authors to revise the manuscript and respond to the comments. This paper will be undoubtedly be highly cited.

Referee #2

(Remarks to the Author)
I co-reviewed this manuscript with one of the reviewers who provided the listed reports.

Referee #3

(Remarks to the Author)
I want to thank the authors for addressing most of my comments as well as those of the other reviewers. I also like the decision of the authors to move parts to the supplement as this has greatly improved clarity of the results. I have just a few remaining small comments/concerns:

- regarding the text on “Gelsolin domain alignment and phylogenetic tree”. What do you mean by this: “integrated framework for comprehensive sequence acquisition and stringent data curation?”. Isn’t this just the canonical/standard approach for building a phylogeny? For gelsolins, it can be helpful to cross-validate whether key interpro domains are present. While you mention the expansion “through a domain-based search across Asgard archaea and Eukaryota datasets”, it is not explained how this was done.
Regarding the phylogeny more specifically: I appreciate that the authors extended their phylogenetic analyses of gelsolin family proteins and used models of evolution that may be more realistic - though I don’t think the authors did model testing to identify the model most suitable to the data and confirm (C60 may be overfitting for single gene trees). Specifically, my concern with this phylogeny was/is that gelsolin homologs in Asgards are generally rather short (1-2 domains) such that there may not be sufficient information to reliably reconstruct the evolutionary relationship of Asgard and eukaryotic gelsolin homologs (and correctly align with eukaryotic sequences that have more than 2 domains). E.g. which part/domain of the various Asgard homologs (e.g. as illustrated in Figure S5 I) was actually aligned across all the sequences? (E.g. it is possible that the evolution of these genes involved duplications of gelsolins domains within taxa further complicating phylogenetic reconstructions)
The authors suggest that the alignment is longer than 5000 AA but it seems that they counted the positions in the raw alignment file, which includes various sequence stretches that occur only in one or a small number of taxa (i.e. unaligned/uninformative sites). Thus the alignment should be counted after trimming (e.g with Trimal and/or BMGE) which extracts aligned and informative sites (prior to phylogenetic analyses). A short alignment does not mean the phylogeny is useless, but its worth to caution that the number of informative sites is limited and inferring a reliable relationship of Asgard and eukaryotic homologs is therefore challenging and potentially impacted by artifacts. Filtering of sites for such short alignments may further decrease information. It is reassuring to read that the “topology remained remarkably stable across different modeling frameworks”, though it was not possible to verify this as only one tree was presented. Please note that various clades have extremely low bootstraps indicating that relationships are not well supported (this is expectable with these short domain proteins but should be presented as such).
Regarding data sharing: it would be helpful, if all alignments (untrimmed, trimmed, filtered) as well as phylogenetic output files were made available in the repository.
Side note: S-Fig. 6 was unreadable in the merged pdf

- legend Figure 4: what is NC?

- movie S10: can you clarify what the Swinholide A concentration is for each of the shown images? E.g. I assume it is sorted per column? Perhaps you could label each column for clarity and indicate concentrations.

- line 282: I would write “Asgard archaeon” here in line with the usage of Asgard archaea throughout most of your discussion and to avoid confusion because Prometheoarchaeon could also refer to the genus (or any taxonomic rank) of Prometheo rather than to the phylum as a whole. Considering that a Heimdall/Hod lineage is more likely to represent the closest sister lineage of eukaryotes, this could be misleading/ easily misunderstood.

- line 283: I would write “Such a process may necessitate…” since we currently do not know what the process of taking up a symbiont was.

- Code availability: will scripts (and/or jupyter notebook files) for data transformation and analysis (proteomics, phylogenetics, plotting etc) be made available as well?

- line 879: I would rephrase to “While we cannot say whether these...”

Referee #4

(Remarks to the Author)
The work addresses the fundamental question of how eukaryotes evolved the ability to translocate using a dynamic actin cytoskeleton. While it was known that Archaeal cells possess archaeal actin that can form filaments along with a small cohort of actin regulators (e.g. profilin and gelsolin-like proteins), whether these cells could make dynamic protrusions and move on surfaces in an actin-dependent manner as do many eukaryotic cells was unclear. Radler et al reveal the ability of two species of Archaea to make dynamic actin-filled protrusions and move either by gliding or protrusion-mediated pulling of the cell body in an actin-dependent manner. These are fundamentally important findings that add important new insights in the evolution of cells, in general, and the dynamic actin cytoskeleton, in particular.

The revised manuscript addresses all of this reviewer’s comments. It also reads more smoothly, making the story easy to follow, and the data and conclusions are clear and convincing. Notably, the description of the proteomics and the phylogenetic analysis (now in the Supplementary Materials) are much easier to follow and appreciate. Overall, this manuscript presents exciting results that provide important new insights into the evolution of eukaryotic cell migration and will leave readers eager for the next chapter of the story.

There are a few, small issues remaining that the authors may wish to address -

lines 206 - 208 (also line 267) - The authors note that an ARF GTPase is relatively enriched in the actin IP pellet, along with a profilin and gelsolins. It is also observed that a BAR domain protein and second profilin are detected, but at levels that are not statistically significant. It would be helpful to readers who may not be intimately familiar with how these proteins are related to the eukaryotic cytoskeleton and migration if the authors were to briefly state why these the ARF GTPase and BAR domain protein may be of particular interest.

line 235 - Treatment of M. peptidophilum with SwinholideA results in shorter, but more numerous protrusions as well as ‘.. deformation of the spherical cell body (Fig S4b-d).’ Since the cell body appears as a small dot in those panels it is not easy to appreciate the deformation that the authors describe. In the panels for cells treated with the highest concentration of the drug (Fig S4d) it almost looks as if two or three cell bodies are clustered together. Is this what is being referred to? Is it possible that the drug might be resulting in multinucleation (perhaps that’s to speculative at this point)? Regardless, it would be good if the authors describe what they mean by deformation here.

line 275 - When listing known classes of regulators of complex migration the authors simply say “myosin”. Given that different myosins can contribute to cellular motility it would be best to use ‘myosins’ here.

Fig 1h - The pink and red lines are not easy to distinguish from each other (the colors look quite similar to each other).

Supp Fig5 b - This panel is so small and rather faint, making it hard to see the plot itself and what is being shown. While reorganizing so many panels may be painful, it might be worth trying to find a way to make this interesting set of data much easier to see.

Legend, Supp Fig 5c - It describes showing ‘disordered domains’ when none are apparent in the panels (but are seen in the model for B35_GelX presented in panel g.

Supp Fig5 f - The orange box is close enough in color to the pink shaded amino acids that it is a bit hard to see.

Version 2:

Reviewer comments:

Referee #3

(Remarks to the Author)
I thank the authors for addressing my final concerns and want to congratulate them for this impressive work.

I have two small comments/questions but do not need to see the manuscript again:
- did you update the reporting summary yet to include the new software used for the gelsolin analyses?
- can you cite explicitly which figures show these new analyses in the main text?
" A more detailed description of the identified proteins and a phylogenetic comparison with previously biochemically characterized gelsolins and profilins is found in the supplementary information." E.g. is it Ext Data 6 or 3k?

Referee #4

(Remarks to the Author)
The authors have addressed all of the issues raised in the previous review. There are two, quite minor issues remaining that should be simple to fix.

Figure captions:

In the legend for Figure 1g, h (Line 471 - 473) says that the arrows indicating fast shrinking protrusions are red, while they appear to be orange in the image (lower row, right hand set of panels) and the plot for the corresponding rate of length change is shaded orange.

Supplementary Information file:

Lines 43-58 The authors describe their initial analysis of putative homologues of PIP lipid synthesis enzymes in the L. ossiferum genome. It would be useful to provide an identifier or accession number for the three enzymes so curious readers can easily find them.

Line 58 This line finishes the discussion of PIP lipids and is then abruptly followed by "The second type of actin binding protein commonly..." that moves into a discussion of gelsolins.
The authors should make this the beginning of a separate paragraph.

Open Access This Peer Review File is licensed under a Creative Commons Attribution 4.0 International License, which permits use, sharing, adaptation, distribution and reproduction in any medium or format, as long as you give appropriate credit to the original author(s) and the source, provide a link to the Creative Commons license, and indicate if changes were made.

In cases where reviewers are anonymous, credit should be given to 'Anonymous Referee' and the source.
The images or other third party material in this Peer Review File are included in the article's Creative Commons license, unless indicated otherwise in a credit line to the material. If material is not included in the article's Creative Commons license and your intended use is not permitted by statutory regulation or exceeds the permitted use, you will need to obtain permission directly from the copyright holder.
To view a copy of this license, visit https://creativecommons.org/licenses/by/4.0/

Reply to Reviewers (Replies in blue)

Referee #1 (Remarks to the Author):

In this manuscript, Radler and colleagues achieve the first live-cell imaging of Asgard archaea, which are anaerobic and notoriously difficult to cultivate, and which are the proposed archaeal ancestors of eukaryotes. They provide novel insights into the behavior and cell biology of this unique group of microorganisms. In contrast to the slow growth rate of Asgard archaea, the authors found that these organisms rapidly and dynamically change their cell shape: constantly extending, retracting, and rearranging their membrane protrusions and cell body.

Further, using automated cell tracking, the authors show that Asgard archaea are motile and provide evidence that the microbes can use their protrusions to pull their cell body across a surface and "crawl" in a manner reminiscent of eukaryotic motility using pseudopodia. The authors find convincing evidence that these observed dynamics are dependent upon the actin filaments found in Asgard archaea membrane protrusions, whose function is highly debated in the field, by showing that the presence of actin inhibitors in the media fully arrests both cell shape changes and movement. The authors show that these groundbreaking findings are consistent amongst different clades of Asgard archaea - both the Lokiarchaea and the Hodarchaea, the Asgard lineage most closely related to eukaryotes, indicating that these novel dynamics are a conserved hallmark of Asgard archaea and that they may have played a role in facilitating eukaryogenesis. Lastly, they propose a potential mechanism for the regulation of actin dynamics in Asgard archaea based on gelsolins and profilins. While the paper's conclusions are mostly supported by the data, more work is required to show that this crawling phenotype, which has not been observed before, is physiologically relevant in Asgard archaea (i.e. does not depend on the imaging conditions).

We thank the reviewer for the positive words and critical feedback. Please find a detailed response below.

Major comments:

- While cells clearly seem alive in all the live-cell imaging assays shown, membrane protrusions dynamics may be the result of cellular stress (e.g. prolonged absence of physical interactions with bacterial symbionts due to differential adhesion to the glass substrate, leakiness of the imaging chamber leading to the presence of oxygen, etc). For instance, the authors fail to observe cell division events. While this may be due to the slow growth rate of Asgard archaea, it may also be due to the imaging conditions causing cellular stress.

We agree that cells are most likely exposed to some sort of stress when taken out of their culture and imaged. However, we think that this probably applies to all strains imaged and the extent of stress depends on the treatment. L. ossiferum and M. peptidophilum grow protected from light and thus will be naturally stressed by light. We observe arrest of motility if we expose cells too strongly to light with long exposure times or high intensities or when they are constantly exposed to oxygen. It is unlikely that the observed motility is solely a result of stress:

a. If this was the case, stress should affect *all* cells imaged and these would behave more homogeneously and move towards a place of lower stress, such as less light exposure or lower oxygen (centre of the chamber).
b. The cells were handled as gently as possible and not centrifuged or exposed to pressure by e.g. placing a coverslip directly on top.

To see more clearly how oxygen stress affects cellular behavior, we prepared a new experiment with flow chambers in aerobic conditions (lab atmosphere) in parallel to an anaerobic flow

chamber. We found L. ossiferum cells to be motile and rearrange their morphology only in anaerobic conditions, arguing that the observed motility is not a result of O_2 induced stress.

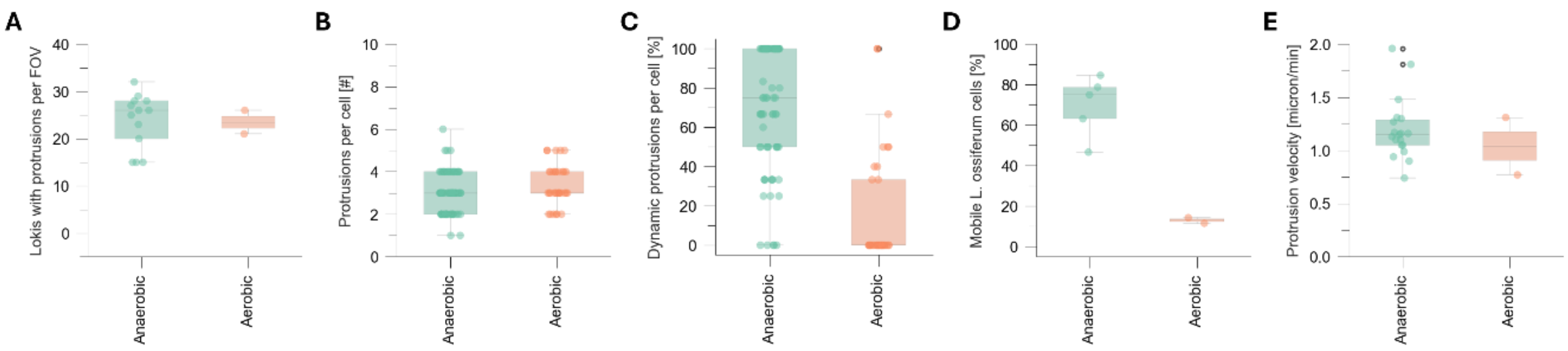

Figure 1: Effect of Oxygen on L. ossiferum motility

Comparison of the number of Lokis/FOV (**A**) and the number of protrusions per cell (**B**) in anaerobic (**green**) and aerobic (**orange**) conditions. Oxygen does not significantly affect the number of attached cells or the number of protrusions. Oxygen rather affects the motility of L. ossiferum: In aerobic conditions, the percentage of dynamic protrusions per cell (**C**) and the number of motile cells (**D**) is reduced. While we find fewer dynamic protrusions in aerobic conditions, the growth speed of those is unchanged (**E**).

This could be ruled out by 1) the use of a redox-sensitive dye such as resazurin to indicate that the imaging conditions are, in fact, anaerobic

As suggested here we have also used resazurin, in fact it was routinely added to the M. peptidophilum medium, also during cultivation. But we did not observe a color change while preparing the flow chamber. (see figure below)

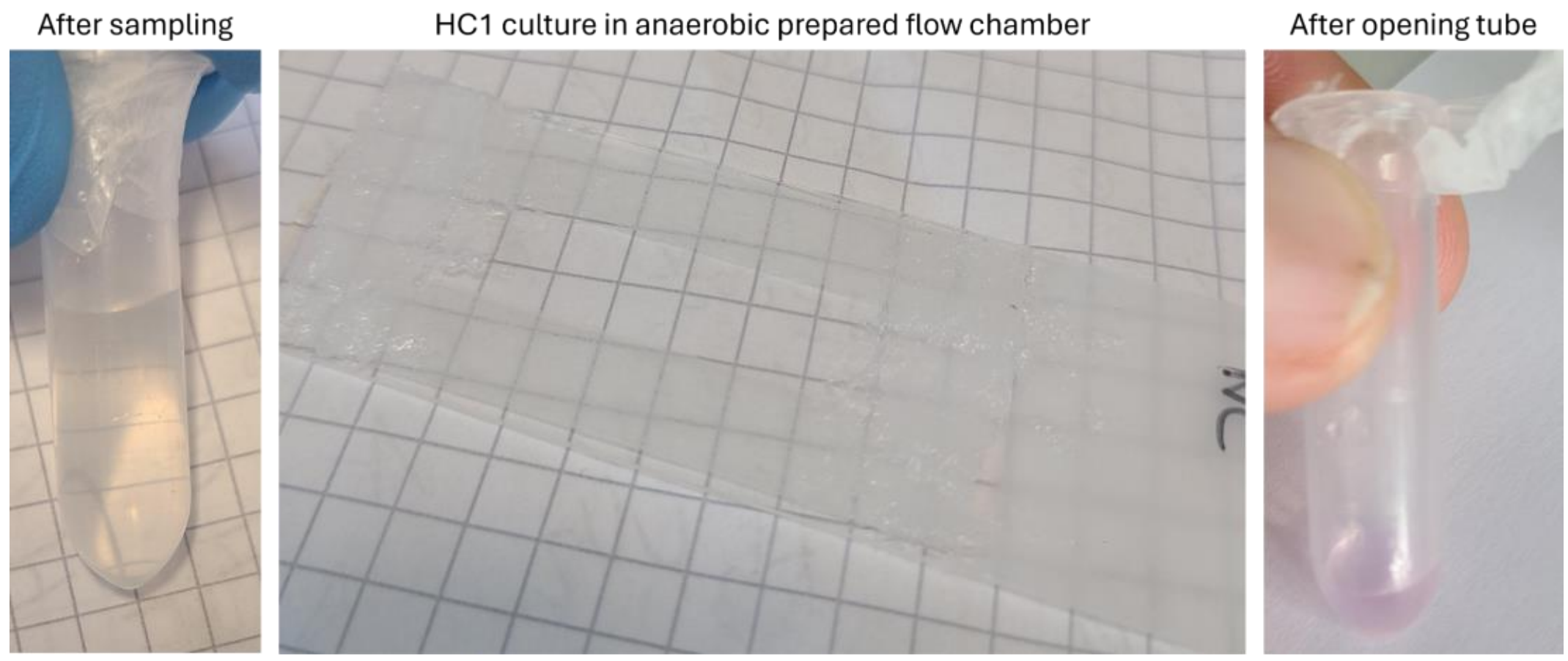

Figure 2: Flow chamber preparation with Resazurin

The pictures show a M. peptidophilum culture after sampling the culture from the anaerobic flask (**left**), after loading the culture into the flow chamber (**centre**) and after exposing the sampled culture to the lab atmosphere (**right**). The color changed (indicating oxygen presence) after opening the tube only.

2) a measurement of cell growth rates inside of the imaging chamber, 3) the observation of cell division events. This is a critical point since this behaviour has not been observed previously.

It is unlikely that cell division could be observed in our current setup due to the slow growth rate of L. ossiferum and M. peptidophilum, which divide approx. once per 7 days (or even less). Through the reviewers comment, we realized that we did not describe the method in sufficient detail: For each experiment, a small volume of culture (100µL) was sampled and kept in flow

chambers for a maximum of 6 hours for “long term imaging” (see below). Hence, growth or a division event would be unlikely to be observed in one field of view.

- It is unclear what the authors mean by “long term imaging”. Are cells continuously imaged for a prolonged period of time (weeks) inside of the imaging chamber, or simply sampled at longer time points after passaging a culture? This should be made clearer throughout the text and method sections. Related to this, it is unclear what data supports the authors’ observations of a “two-week imaging experiment” as Fig. S1 reports the results from a “7-day imaging experiment”. Also, it is unclear how imaging was done. The main text only describes a vaseline-sealed chamber, but the methods describe the flowing of gas mixes. Does this mean the authors poked a gas line needle through the Vaseline and made an exit hole on the opposite side to facilitate this gas flow? Or did they place the glass slide + double sided + coverslip into an additional chamber where they can control the atmosphere? Additionally, where specifically were the anaerostrips placed to monitor the presence of oxygen?

We apologize for not having described this more clearly. Short-term imaging refers to experiments where the flow chamber was sealed and kept in an anoxic environment for 2 hours after loading the culture. The chamber is then imaged in the upright phase contrast setup without environmental oxygen control for a maximum of 30 minutes, before being placed back in an anaerobic environment.

Long term imaging refers to experiments done with the anaerobic DIC setup for up to six hours (not longer). The flow chamber was mounted using a custom-printed 3D holder and a Nikon microscopy stage inset designed for the dimensions of a microscopy slide. To accurately control the conditions of the imaging environment, a gas chamber was placed on top of the imaging inset. The Anaerostrip was placed within the gas chamber and the atmosphere was controlled using an Ibidi Gas Mixer connected to N_2 and CO_2. The pictures below show the long-term imaging setup.

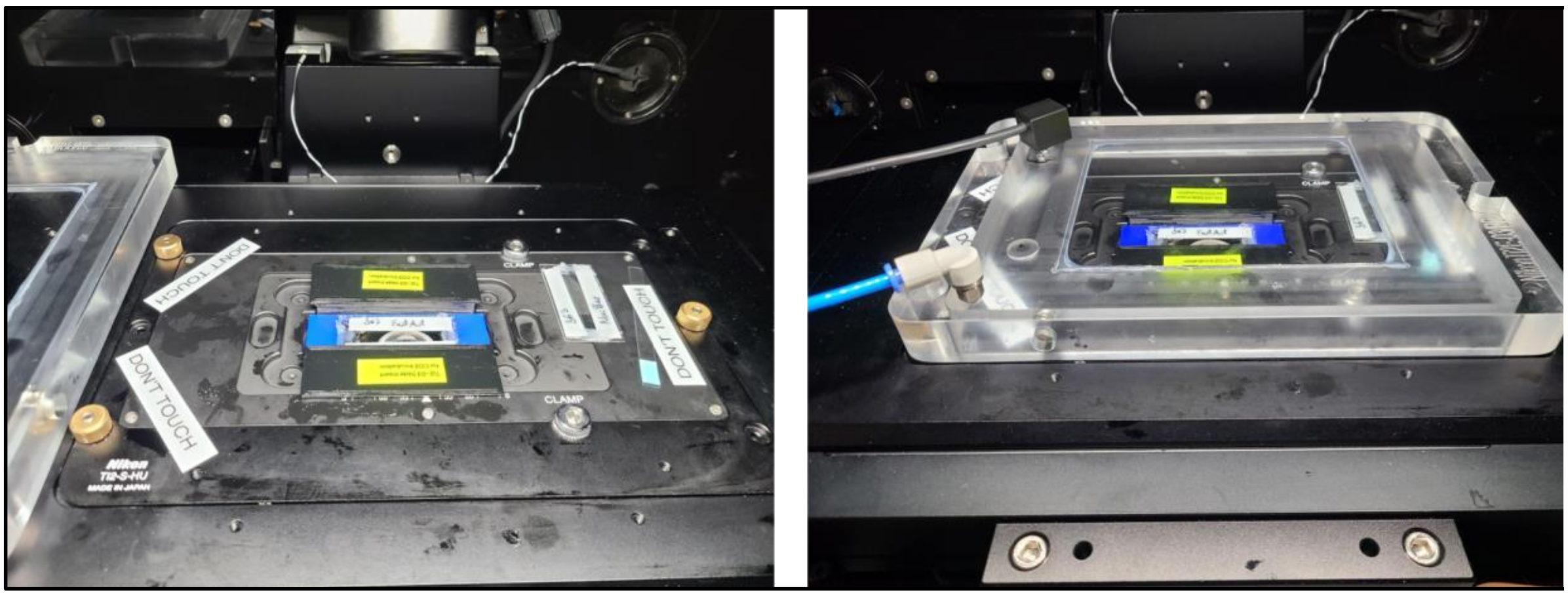

Figure 3: Anaerobic setup for inverted DIC microscopy

Pictures of the anaerobic imaging setup with an open and closed gas chamber. The Anaerostrip is placed within the gas chamber, as indicated by the red arrow.

When we refer to the two-weeks imaging experiment, we have repeatedly sampled the same culture for experiments, but we did not keep the culture for two weeks in the same flow chamber. **Fig. S1F** shows the results of repeated sampling of a single culture throughout the growth curve (sampling timepoints 19/24/25/27/28/31 days), which is what we referred to as 2 weeks in the original manuscript. We now describe this more precisely as “twelve days of repeated sampling of the same culture”. We have not established continuous cultivation and imaging within a flow chamber for several days (see arguments above for point 1)

We now adapted the scheme in **Fig. 1E** and describe the imaging setup as follows in the main text:

"To differentiate between these possibilities, we established time lapse microscopy by upright phase contrast (PC) and inverted differential interference contrast (DIC) microscopy (**Fig 1e, Movie S1**). For both methods, the flow chambers were prepared in anaerobic conditions in a N_2:CO_2 atmosphere and then sealed. PC microscopy was used without environmental control of the atmosphere for short term experiments, lasting at most 30 minutes. For DIC microscopy, the surrounding atmosphere of the flow chamber was kept at 85:15% N_2:CO_2 by constant gas flow into the chamber and experiments were recorded for up to six hours."

Why 22C vs. the standard growth temp of 20C? Was this the temperature used for both L. ossiferum and M. peptidophilum? Why 15:85 N2:CO2 and not the standard 20:80 N2:CO2? Won't this alter the pH?

The installed temperature control system was able to reliably cool down to 22°C, close to the optimal growth temperature of L. ossiferum (20°C). Gas settings were limited by the Ibidi mixer, which supports flow of maximum 15% CO_2. According to the Henderson Haselbach equation, the pH at 22°C and 15% CO_2 rises by 0.1, compared to 20°C and 20% CO_2. While this is not ideal, we think this minute potential change will not be significant enough to elicit such dramatic morphological changes in L. ossiferum or M. peptidophilum.

The presented phase contrast experiments of M. peptidophilum were also performed at 22°C. In the meantime, we also repeated DIC experiments at 30°C (growth temperature of M. peptidophilum) and observed similar, highly dynamic behavior. Representative micrographs are shown below.

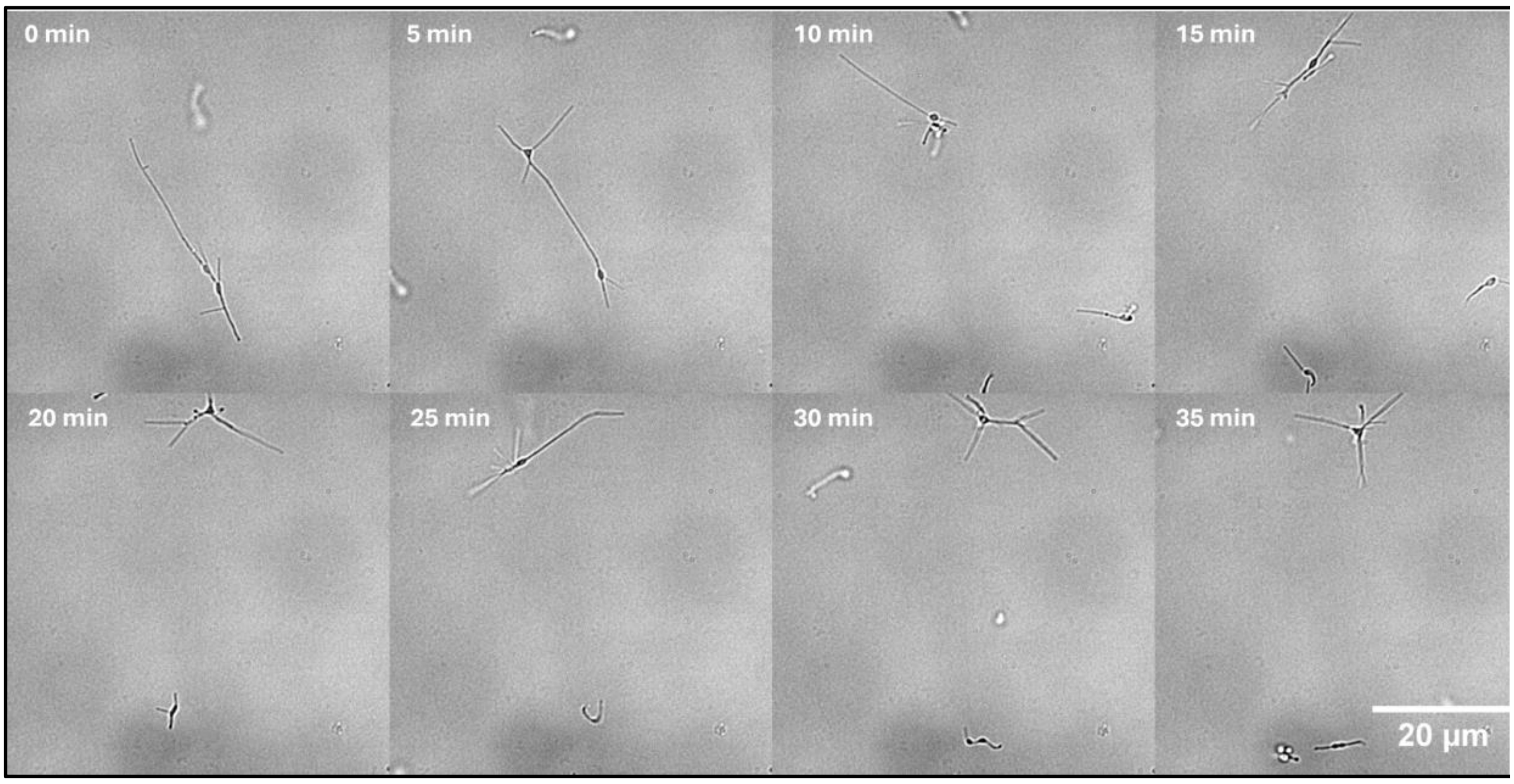

Figure 4: Representative micrographs of HC1 cells imaged at 30°C

Representative time lapse micrographs of HC1 cells at 30°C are shown in 5-minute steps. The cells still dynamically rearrange their morphology and crawl along the glass.

We have now added a more detailed description of the imaging setup in the Methods section (paragraph: Preparation of flow chambers and live imaging) and hope the reviewer finds this improved.

- Page 5 (and throughout the manuscript): In describing motility patterns and speeds in Asgard cells, the authors compare them with the crawling speed of eukaryotes and the swimming speed of bacteria, archaea and flagellates. This draws a rather misleading or incomplete picture as different, unrelated structures mediate swimming in archaea (archaealla), bacteria (flagella) and flagellates (cilia). Therefore, swimming speeds should be described separately. Moreover, the authors fail to compare Asgard cell motility with type IV pilus-mediated twitching motility in archaea (in the order of 18 µm/min; see Charles-Orszag et al. 2024, DOI 10.1038/s41467-024-49101-7) and bacteria (in the order of 60 µm/min; see Merz et al., 2000, DOI 10.1038/35024105). This should be added to the result section and discussed further.

Thank you for this comment. The reviewer is correct that we summarized swimming speeds from different domains of life, without pointing out the different underlying molecular machines and we have corrected this as follows:

"This is much slower than swimming motilities exhibited by archaea using an archaellum (0.18 – 22.8 mm/min), flagellated bacteria (1.5 mm/min), protists using cilia or flagella (7 - 42 mm/min) or bacterial twitching motility (60 µm/min)."

We also apologize for overlooking the reference describing twitching motility in archaea, which is the first description for surface-based motility in archaea and groundbreaking work. Thank you for pointing this out. We have included it now and we think it is better to compare the reported cell displacement speed of 1.1 µm/min (based on the reported displacement of "The average track displacement over 5 min was 5.7 ± 0.44 µm"[1]) instead of the 18 µm/min, which is the reported average pili retraction speed (Fig. 2d in [1]).

So, the cell displacement was within the same range as reported by us, even though the movement using pili or membrane protrusions is fundamentally different. We now compare our observed migration rates to the reported twitching motility:
The revised text reads: "Instead the motility of L. ossiferum is within the range of reported crawling speeds of eukaryotic cells (0.1 – 20 µm/min) and the type IV pili-driven twitching motility of Sulfolobus acidocaldarius (1.1 µm/min), the hitherto only described surface motility in Archaea."

- The authors state that Asgard cells move around by pulling and gliding. However, they do not explain why they rule out pushing. Also, it is mentioned that gliding may be due to actin treadmilling. However, this could also be explained by simultaneous actin polymerization in the front and active actin depolymerization in the rear of the migrating cell body. Lastly, movies of motile cells clearly show that cell bodies experience two distinct modes of displacements: slow displacements over short distances that seem to be mediated by gliding, and larger, sudden displacements that appear to be mediated by the rapid buckling and surface detachment of membrane protrusions located in the rear of the cell (relative to the direction of cell migration) followed by their reabsorption back into the cell body. In other words, it looks as though membrane protrusions act like dynamically adhesive springs under tension. All these should be more carefully described and reinterpreted.

We thank the reviewer for this comment! We ruled out pushing using the analysis shown in Fig. S2H: Here we correlate the movement direction of the cell body with the movement direction of protrusions. For pushing, these two movements would be directed in opposing directions (body away from protrusion) and result in a negative value measured. However, we observe a weak bias towards positive values, meaning that the direction of protrusions and cell bodies is positively correlated (toward the same direction).
The two other modes of motility have been quite accurately summarized by the reviewer, so we have rephrased the paragraph describing the two major types of motion observed in the revised

text accordingly (see text above Fig. 2). We also agree with the reviewer that some sort of treadmilling of individual filaments or filament bundles is complicated to prove or rule out without expression of tagged actin variants followed by their spatial bleaching and/or photoactivation, which is not feasible at present. So, we have also added the alternative mechanism suggested by the reviewer, leaving the precise mechanism to be determined in future investigations.

- Fig. 4: Authors should also have plots similar to Fig. 3E-H to show that/if these dynamics were quantified. They don't appear to have quantified the number of M. peptidophilum per FOV, protrusion per cell, protrusion growth rate, or % of mobile cells in the presence of Swin A. The latter three are important to quantify if they want to make the claim that SwinA arrested the rearrangement of cell morphology and migration.

As requested, we have now performed titration experiments with M. peptidophilum cells using 5/50/500 ng/mL Swinholide A and the appropriate controls. The results are displayed in the new Figure 4 and described in the text as follows:

"We then performed a titration with Swinholide A to see if the motility of M. peptidophilum cells is also dependent on a dynamic actin cytoskeleton **(Fig. S4a)**. We find a decrease in the density **(Fig. 4h)** and motility **(Fig. 4i)** of adherent M. peptidophilum cells, similar to experiments with L. ossiferum. Interestingly, we do not find a reduction in the number of protrusions per cell, but a decreased average protrusion length and deformation of the spherical cell body **(Fig. S4b-d)**. The formation of new protrusions and growth of persisting protrusions was arrested with concentrations higher than 50 ng/mL SwinA (**Fig. 4j, k**). Thus, the presence of Swinholide A arrested the rearrangement of cell morphology and migration as observed in L. ossiferum (**Supp. Movie S12**)."

- Page 10: The part addressing the potential role of gelsolins and profilins may not need to be an entire section in the main text. It could be moved to the supplement and/or the Co-IP experiments could be introduced briefly at the end of section 3 to provide insight into what proteins might be regulating the lokiactin cytoskeleton which appears to be driving the locomotion in these cells. This last section feels a little underdeveloped and out of place, it would make more sense to present it in the context of section 3 after it is shown that actin appears to be involved in the movement of the Loki cells.

We are grateful for this comment and agree. We have shortened this section and, following the reviewer's suggestion, moved the section up, placing it in shorter form after the Lokiactin inhibition experiments. We have moved the more detailed results and discussion about the different types of gelsolins into the supplementary information.

- In the discussion, the authors claim that examples of cell motility in the absence of proteins such as actin polymerases or myosin have been described before. However, some of the three references cited do not actually support this. Ref. 55 actually reports actin-independent cell motility based on MSP (major sperm protein). Ref. 56 reports the mechanisms of myosin-independent contraction of actin rings, not myosin-independent actin-based motility. Please revise accordingly.

We have adapted the quoted references to exclusively refer to papers that refer to actin-based motion without the presence of myosins or formins. The text reads: " However, examples of motility in the absence of these proteins are known, as in red algae and in Trichomonas vaginalis, but understood in less detail[2,3] "

Minor comments:

- Throughout the manuscript: it is "poly-L-lysine" not "lysin"

This has been replaced – thank you.

- Page 1: It appears that the authors based their measurement of cellular protrusions diameter on FWHM values obtained through DIC imaging (Fig. 1D). Could the authors clarify why they did not use values obtained through electron microscopy instead? Even though SEM may lead to overly dehydrated cells and affect the aspect of membrane protrusions, these protrusions have been observed before through cryoEM imaging which likely represent cells in their near-native state (Rodrigues-Oliveira et al., 2023; Imachi et al., 2020; Imachi et al.,2025). Could the authors describe how their DIC measurements compare with previous cryoEM data? This is important as it impacts their estimate of increase in cell surface area. Also, please show representative SEM data that were used to measure protrusions diameter.

For our light microscopy measurements, we quantified protrusions of adherent L. ossiferum, while previous SEM and cryoEM experiments were performed on floating L. ossiferum cells. We have measured protrusion diameters and the corresponding surface area increase (assuming a cell body surface area of 3.69 μm^2) from different methods and now have included additional protrusion measurements from recent PC and DIC microscopy experiments into the data shown in **Fig. 1d.** The detailed measurements and their effect on the surface area are summarized in the table provided below.

What	PC	DIC	SEM	cryoEM
**Protrusion diameter [nm]**	527 ± 117	245 ± 31	117 ± 16	83 ± 34
**Surface of one protrusion [μm^2]**	6.77	3.04	1.42	1.01
**Surface of five protrusions [μm^2]**	33.86	15.20	7.11	5.05
**Ratio of surface area increase**	9.18	4.12	1.93	1.37

Table 1: Comparison of protrusion diameters and corresponding surface areas from quantification with different imaging methods.

This shows that we did indeed find strong differences in the measured protrusion diameter, with light microscopy approaches certainly over-estimating their true thickness for technical reasons and with the vitrification prior to cryo-EM certainly constituting the gold standard for maintaining their near-native structure and configuration. In all cases, protrusions increased overall cell surface areas.

It is difficult to exclude at present that adhesion to the substrate might thicken these types of protrusions, at least to some extent, also contributing to the large differences between distinct measurements. We note that SEM and cryoEM experiments were performed on floating L. ossiferum cells, which seem to contain more protrusions as seen in the representative SEM images below.

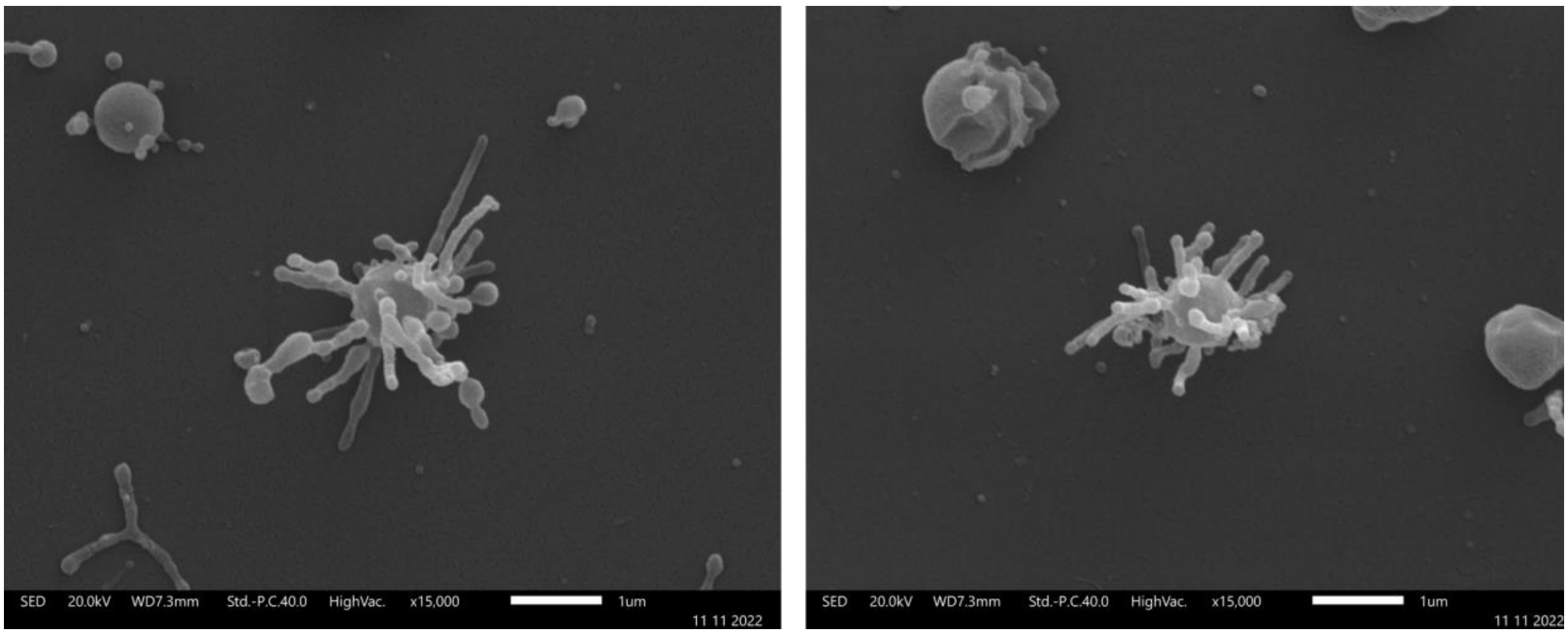

Figure 5: Representative SEM micrographs of non-adherent L. ossiferum cells

We have decided against inclusion of those images into the manuscript, as our focus is on dynamic processes (membrane deformation and motility), and the overall cell shape, as revealed by EM, has extensively been discussed in earlier papers.

Together, we discuss limitations of the quantification of protrusion diameters as follows:

"Due to the fineness of the protrusions, we are potentially overestimating the true increase in cell surface area, but even with the most conservative estimates from cryo-EM (average protrusion diameter of 83 nm), the presence of five protrusions doubles the available surface area."

- **Page 8:** Similarly, please indicate how membrane protrusion diameter was measured for M. peptidophilum cells.

In the previous manuscript version, the reported protrusion diameter of M. peptidophilum was quantified from phase contrast images only. We have now quantified M. peptidophilum cells imaged by DIC, offering apparent higher resolution. The measured diameter with DIC is 343 ± 25 nm compared to the PC estimation of 599 ± 10 nm. We adapted **Fig. 4b** to show both values in the manuscript for better comparison to L. ossiferum.

- Could the authors indicate the percentage of L. ossiferum enrichment cultures that were chosen for live-cell imaging in the main text? Similarly, please report the same value for M. peptidophilum as it is not reported in the methods section.

We have added the enrichment levels of both cultures now in the Results section, and the corresponding text reads:

"..for imaging experiments, we chose cultures with more than $1*10^6$ L. ossiferum cells/mL and a relative abundance of around 20 to 60%."

"This strain is a representative of the Hodarchaeales lineage and was isolated as a pure co-culture with Methanocalculus sp. MC2 with a stable population ratio of HC1:MC2 = 70:30."

And in the Methods section:

"Live imaging of M. peptidophilum was performed on cultures in the late exponential to the early stationary phases with a population ratio of HC1:MC2 = 70:30%."

- **Page 4:** Replace "life staining" with "live staining".

Done.

- **Page 4:** No data in the main or supplementary figures seem to corroborate the observation that NucBlue-stained chromosomes undergo fusion. Please clarify.

Here, we wanted to point out that the DNA signal is not static and sometimes undergoes dynamic rearrangements within the cell body, as seen in **Supplementary Movie 5 (right)**. We have now adapted the phrasing of the sentence to:

"Such morphological changes were not restricted to the cell body alone, as the position of the chromosome, revealed by live staining with NucBlue, occasionally rearranged dynamically."

- Fig. 1H: It would be visually easier to understand if the authors used more intermediate time points between 0 and 3 min, 3 and 6 min etc. If space is limiting, panel G could be removed as the timelapse clearly shows protrusions dynamics.

We adapted **Fig. 1h** according to the reviewer's suggestion. During this, we realized that the position of the blue arrow (=shrinking protrusion) was shifted in the last part of the montage. We have adapted the figure accordingly.

- **Fig. 1I**: The Y-axis can not read "protrusion growth rate" in the case of protrusion shrinkage and catastrophe rates unless these were represented as negative growth rates.

We thank the reviewer for pointing this out and we have changed the axis label to "**Protrusion length change rate**".

- **Fig. 1K**: Could the authors label the difference between top and bottom graphs? Alternatively, this could be described in the text.

We added subtitles to the graphs. The difference in this panel was the acquisition speed and the used microscopy modality. Top is an example of a movie recorded with phase contrast (2 sec/frame), the bottom image is data recorded with DIC microscopy (1 min/frame).

- **Fig. 1**, legend B, C and D: Please indicate sample size for all three.

We added the corresponding sample sizes.

- Page 7: The rates of protrusion growth actually drop by ~50%, which is more than "slightly"

We removed the word "slightly" to describe this observation more accurately.

- Fig. 4, title: Replace with "actin-dependent" and add "and surface motility" or "crawling motility" to best describe the observations reported in the figure.

We changed the title to "M. peptidophilum displays actin-dependent morphological rearrangements and surface motility".

- Fig. 4H: The SwinA treatment does not seem to affect the number of protrusions in this example. Could the authors clarify?

We have now performed Swinholide A titration experiments with M. peptidophilum and discussed our results in the comments to this reviewer under 'Fig. 4' (see above).

- Page 7: SiR Actin and its FastAct derivatives are thought to specifically bind F-actin in eukaryotes and not monomeric actin. Based on previous cryo-EM images of L. ossiferum, the cell body does not appear to contain many actin filaments, especially compared to the protrusions. Why, then, do the author's think FastAct labels the central cell body so brightly? Please discuss.

Lokiactin filaments in the cell body have been observed before for L. ossiferum (compare **Rodrigues et al., 2023, Fig 4d**) and M. peptidophilum (**Imachi 2025, Figs. 2l and 2n**). An important difference to take into account for cryo-EM is the thickness of the observed cell

compartment. The cell body is much thicker compared to the protrusions, and while a tomogram slice of a protrusion might show all actin filaments, filaments within the cell body might be missed. Furthermore, filaments in the cell body can be oriented at various angles compared to the electron beam and might thus be more easily missed. Based on these considerations, we might tend to underestimate the overall amount of filaments present in cell bodies as compared to the much thinner protrusions where they are tightly packed.

On the other hand, the relative thickness of the cell body will over-estimate fluorescence signals (relative to protrusions) when using widefield imaging, simply because the technique will pick up out-of-focus fluorescence in cell bodies missing in the thin protrusions. This problem will potentially be reduced by confocal imaging, but we have refrained from performing such experiments at this stage of the project as cells will likely be subjected to higher laser powers and thus more stress. Future experiments will address such issues making sure to get access to spinning disk as compared to point-scanning confocals and likely require additional optimization beyond the scope of the current study.

- Page 7: Did the authors mean to say “low concentrations” here instead of high? Most of the actin inhibitors should work at low concentrations in eukaryotes.

For initial actin inhibitor tests, high concentrations were used to preselect suitable compounds. After preselection, we performed titration experiments on selected candidates. To clarify this aspect, we have now adapted the text to:

“In initial tests, we used high concentrations of actin inhibitors to identify a compound suitable for L. ossiferum cells and avoid overlooking effects due to compromised affinities of the inhibitors to Lokiactin.”

- Page 7: The authors describe that the number of protrusions decrease upon treatment with SwinA. Since the length of protrusions remains constant when the cells are dynamically rearranging, is this loss of protrusions accompanied by an increase in cell body diameter or an increase in length of the remaining protrusion?

We did not explain well in the manuscript that the cell body morphology is affected by Swin A and we find more non-spherical (=disturbed) cell bodies. When we quantified the diameters of the remaining, spherical cell bodies we found no significant differences in the diameters of the cell bodies **(Figure 6 below, panels A, B),** neither for L. ossiferum **(orange)** nor for M. peptidophilum **(green).** We observed a strong increase in the percentage of deformed cell bodies **(Figure 6 below, panels C, D)**. The quantification of the percentage of deformed cell bodies of L. ossiferum and M. peptidophilum have now been added Supplementary Figures 3 and 4, respectively.

The protrusion lengths are hard to quantify, as protrusions at high SwinA concentrations do not tightly adhere to the glass and tend to lift up into the medium. However, in the case of M. peptidophilum, we managed to quantify the protrusions as they appeared thicker and are thus easier to quantify also in these particular conditions. We did find more protrusions at high SwinA concentrations, but their average lengths were markedly decreased, indicating impaired elongation of protrusions. This quantification is now included in **Supplementary Figure 4.**

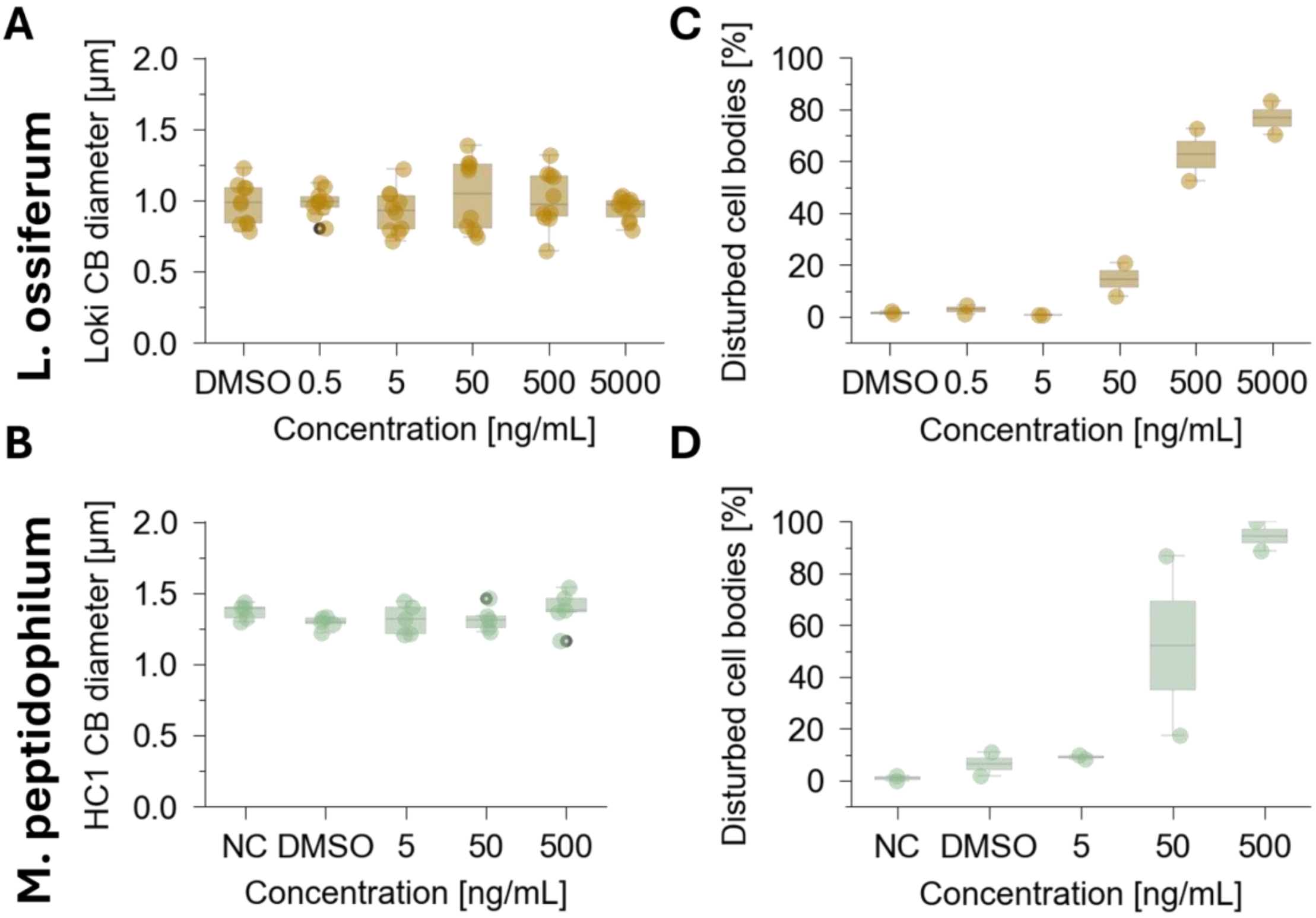

Figure 6: Effect of Swinholide A on cell body diameter and morphology

Quantification of cell body diameters and the percentage of disturbed cell body morphologies with increasing concentrations of Swinholide A for L. ossiferum (A, C) and M. peptidophilum (B, D).

- Page 7: Swin A works by sequestering actin dimers AND by severing existing F-actin. Since the fluorescent signal was not lost completely, does this suggest that the FastAct stain may not be specifically binding to F-actin and may also be binding to monomeric actin? This could explain why the cell body is so bright.

At this point we simply do not know enough about the ultrastructures of Lokiactin filaments and their monomers. That's why it's difficult to provide answers to such questions that can be substantiated by solid, citable data. However, it is clear from studies on eukaryotic actins that (i) FastAct constitutes a jasplakinolide derivative and (ii) the latter binds to eukaryotic actin filaments at a vertex point of three individual monomers, with the single binding surfaces exhibiting minimal to unmeasurable individual affinities and only interacting through the avidity of the three binding sites[4,5]. Based on these considerations, one would assume jasplakinolide-based probes to display no binding whatsoever to actin monomers, although one study reported at least a measurable affinity of Sir-actin and some comparable probes app. 100x lower than for F-actin[6]. So even if this were transferable to Lokiactin, how relevant can 100x decreased binding be in situ, i.e. in the presence of additional actin binding proteins – a definite answer to this and similar questions will first of all require more structural insights, both at the monomer and filament level and ideally in the context of respective actin binding compounds. In the absence of such obligatory pieces of evidence, we would like to refrain from speculating too much on such questions at the current stage of knowledge.

- Fig. 4D: Please indicate how many experiments were performed to measure the 31 protrusions.

We have added the respective sample sizes to the figure caption (50 protrusions from 30 cells and 6 cultures). During revision, we performed a few more experiments with M. peptidophilum and increased the sample size accordingly.

- Page 10: Please remind the reader how many profilin and gelsolin homologs were known to be encoded in these genomes.

We have adjusted the sentence, see the reply to the comment below.

- Page 10: Only 8 complete genomes appear on GTDB. There are 8 genomes classified as complete and 7 genomes classified as a "chromosome" but that are allegedly not complete. But maybe this is the 15 the authors are referring to? Please clarify.

The reviewer is correct that we referred to the 8 genomes and 7 "chromosomes" in the initial version. We now described this more accurately:

"These homologues were identified in the majority of the eight closed, seven chromosome-level and 396 Asgard meta-genomes (according to GTDB in 2025) with up to five profilin and nine gelsolin homologues present in a single Asgard genome."

- Page 10: "myo-inositol-1-phosphate" refers to a molecule. What is the enzyme the authors are referring to?

We are referring to the enzyme myo-inositol-1-phosphate-synthase (or **Inositol-3-phosphate synthase, MIPS)**. The "synthase" was missing and we thank the reviewer for catching this. The section in question has now been moved to the Supplementary Information.

- Fig.5, title: Replace with "Co-immuno"

The data from Figure 5 has been moved into Figure 3 and Supplementary Figure 5, thus this replacement is not necessary.

- Discussion: It would be more exhaustive to also mention other known membrane reservoirs in eukaryotes such as caveoleae (Sinha et al. 2011, DOI 10.1016/j.cell.2010.12.031).

We have cited the caveoleae references and have adjusted the text as follows:

"Since the membrane surface appeared constant, these shape changes do not seem to be facilitated by synthesis of novel lipids, uptake of lipids from syntrophic partners or externalisation of intracellular lipid compartments, such as caveolae."

- Discussion: remove "e.g." in "as e.g. suggested"

We adjusted the text accordingly.

- Discussion: replace by "will be THE subject of future work".

The text was amended as requested, thanks.

- Discussion: The authors propose that actin regulation may be calcium-dependent. Would it be possible to perform experiments using calcium ionophores to test the hypothesis?

We now performed experiments on L. ossiferum cells that were incubated with the calcium ionophore A23187 at increasing concentrations (1-1000 nM) for 1.5 hours.

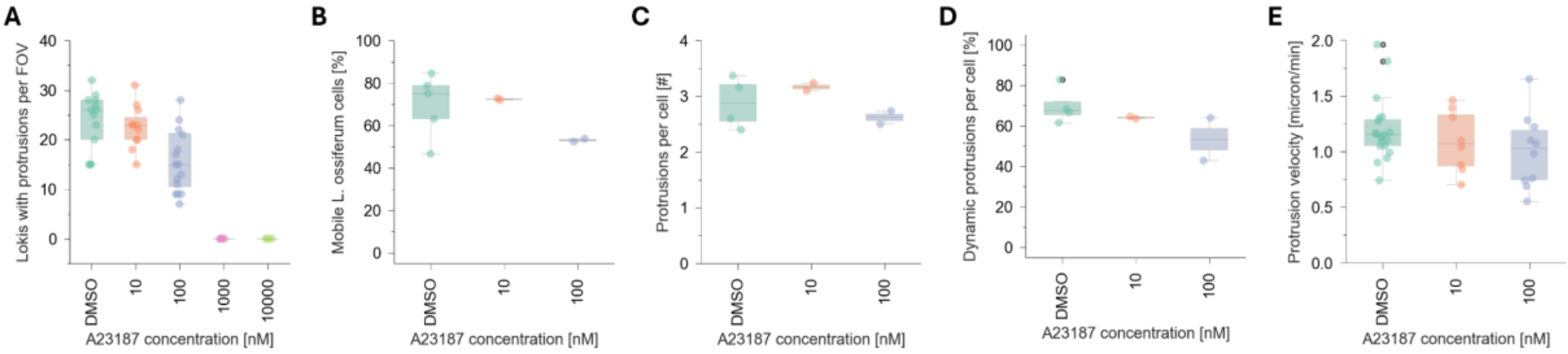

Figure 7: Effect of the calcium ionophore A23187 on L. ossiferum

Effect of increasing A23187 concentration on the density of L. ossiferum per FOV (A), the motility of L. ossiferum (B), the number of protrusions per cell (C) as well as the percentage of dynamic protrusion per cell (D) and the growth rate of protrusions (E).

Indeed, we have failed to find any adherent L. ossiferum cells with protrusions at concentrations above 100nM A23187 (**A**). We found a slight drop in the mobility (**B**) and the percentage of dynamic protrusions per cell (**D**) at 100nM A23187, but no significant change in the number of protrusions (**C**) nor the velocity of the growing protrusions (**E**).

We want to point out that it is unclear if the Ionophore A23187 works the same way for Archaea as it does for eukaryotes and what intercellular processes it affects. While there seems to be a small effect in terms of motility, we cannot be certain whether this is a specific effect of the ionophore. Hence, we decided to not include this dataset in the manuscript.

An additional aspect is that we might miss immediate effects of the ionophore, as the current method requires incubation before imaging because we can't access cells in the flow chamber through manipulation from outside. In future work, we aim to establish micromanipulation experiments, to study the immediate effect of compounds, such as A23187, on cell morphology and dynamics. We thank the reviewer for this insightful suggestion.

We have removed the sentence referring to $CaCl_2$ from the discussion and refer only to the results from the Robinson group in the results section concerning Figure 3, as we cannot precisely interpret the calcium ionophore on Loki cells and now find that this comment was too speculative.

- Discussion: Please discuss whether the last common Asgard ancestor is thought to have already possessed lokiactin, gelsolin, profilin, and enzymes involved in the synthesis of PIP-like headgroups.

Due to the high conservation of Lokiactin among all identified Asgard lineages to date, we think it is likely that the last common ancestor already possessed Lokiactin. Similarly, gelsolins and profilins have been found in most genomes of Asgard Archaea identified to date[7].

We refrain from discussing the more complicated evolutionary path of PIP-like headgroups, since we have shortened the whole paragraph in the text according to the reviewers request and have also moved gelsolin and profilin protein discussions to the supplementary material. In our view, adding the PIP-like headgroups is beyond the scope of the current study.

- Discussion: Regarding current models for eukaryogenesis, please also cite the model by Krupovic et al., 2023 (DOI10.1038/s41564-023-01378-y).

We added the reference as well as the recent paper by Kay et al. formulating the 'CALM' (Complexified Archaeon, Late Mitochondrion) model[8].

- Discussion: regarding “deep marine sediments", this is rather speculative. Why would the sediments need to be deep? Most eukaryogenesis models involve at least some oxygen which was not present in the deep ocean two billion years ago.

The idea of deep sediments stemmed from the observation that Asgard archaea tend to live at considerably higher abundances in deep marine sediments. However, to avoid speculation, we have now removed the word “deep” from the discussion.

Referee #2 (Remarks to the Author):

I co-reviewed this manuscript with one of the reviewers who provided the listed reports.

Referee #3 (Remarks to the Author):

In their article "Dynamic protrusions mediate unique crawling motility in Asgard Archaea (Promethearchaeota)" Radler et al used live cell microscopy to investigate the potential of cell motility for the first cultivated members of the Asgard archaea. The authors provide stunning images/movie clips indicating that the investigated organisms can dynamically change cell shape and use their protrusions for active crawling motion powered by their actin cytoskeleton. Considering that a lineage within Asgard archaea is hypothesized to have given rise to the eukaryotic nuclear lineage about 2 billion years ago and that Asgard archaea encode various so called eukaryotic signature proteins, research shedding new light into the cell biology of members of this group is important: the authors employ among others advanced imaging techniques that suggest that cellular motility of extant representatives of the Asgard archaea resembles eukaryotic crawling motility while being unusual for prokaryotes.

The approach and data quality seem overall robust and reliable, with the caveat that I am not an expert in microscopy. I provide some minor comments regarding methods and data below.

We thank the reviewer for their positive assessment of our work.

Minor comments:

- consider to also acknowledge the alternative formal classification based on SecCode: Asgardarchaeota

We have added this to the introduction and now write:

"Since their discovery a decade ago, Asgard archaea <u>(also Asgardarchaeota and now formally classified as the phylum Promethearchaeota)</u> have inspired novel models for eukaryogenesis."

- you mention: "the measured migration velocities of cell bodies were remarkably similar to the observed protrusion growth rates, suggesting a connection between these two."

I am wondering what the causal link would be and whether this could just be coincidence?

At the current stage we hypothesize that – in analogy to eukaryotic paradigm -both, the growth rates of protrusions and the displacement of the cell body depend on actin filament dynamics. To power protrusion growth, actin filaments are thought to insert monomers at the membrane. These insertions push the membrane forward and the filament backward. By coupling to the substrate, rear-ward gliding filaments can generate friction to pull the cell body forward. The types of motility discernible by our microscopy approaches are in line with this mechanism. Even if individual protrusions might diverge from the average translocation rate of the cell body, it is plausible that the collective rates of protrusion and that of cell body translocation will align with each other. Nevertheless, we agree with the reviewer that a simple coincidence between the two as opposed to a more mechanistic connection cannot be strictly excluded. We have thus toned down the wording in this context in the revised manuscript text, allowing further studies to confirm or refute these assumptions in the future.

We now write:
"The median migration velocity of cell bodies was remarkably similar to protrusion growth rates indicating a potential connection between those motility parameters."

- how do you define "in front" of the body? Is there a way to determine the front in the absence of movement towards something?

With front, we refer to protrusions directed towards the same direction as the cell body is moving. In the absence of movement, we can't determine a cell front.

- you mention “We found homologues of three enzymes involved in PIP headgroup synthesis”. Where did you find those: i.e. encoded in the genome, proteome, in conncectin with the actins...? Which are these?

We identified potential homologues of PIP headgroup synthesis enzymes by modelling the structure of all proteins encoded in the genome of L. ossiferum and a subsequent structural homology search with FoldSeek.

However, we have now moved this section on profilins and gelsolins into the supplementary information, as recommended by reviewer #1. Furthermore, since several reviewers pointed out that this last section was too preliminary, we have refrained from reporting or discussing PIP headgroups in the revised manuscript. Instead, we have moved the remaining results of Co-IP and proteome data to Figure 3, and a detailed discussion on these data into the supplementary information.

- The section on “Gelsolins and profilins as putative regulators of Lokiactin polymerization” could be presented a bit more clearly: i.e. which proteins were detected in the proteome, and which did you look at because they are encoded in the genome: “A phylogenetic analysis of L.ossiferum gelsolins...” Do you mean all gelsolins encoded in the genome? Or all gelsolins detected? In line with this, in Fig 5E: can you provide accession numbers to the previously characterised gelsolins?

We thank the reviewer for pointing this out. We have now moved the experiments from our Co-IP and proteomic analysis to Figure 3 and streamlined the discussion. We have added a more detailed explanation of our findings and the analysis pipeline into the supplementary information. We now also list accession numbers of all proteins mentioned in this study in **Supplementary Table 2** and have added references to the papers in which the gelsolins and profilins were initially characterized.

- While I agree with the authors that “the described plasticity and motility might have played an important role in eukaryogenesis...”, the authors could perhaps mention the caveat that these analyses are based on two extant model systems which may have different cell biological features than the archaeal ancestor of eukaryotes.

We thank the reviewer for this point and agree.

The text reads now: "While this study was performed with two extant model organisms, that will differ from the archaeal ancestor of eukaryotes 2 billion years ago, it is conceivable that the latter used protrusions to move towards potential interaction partners, connect to them to facilitate nutrient exchange and eventually engulf them."

METHODS

- It would be great if you can provide more details about the media and cultivation conditions for the enrichment cultures here (instead of referring to previous articles). Likewise, it would be helpful if you can provide additional information about the enrichments used for the various downstream experiments (some of these aspects are mentioned sometimes but not consistently): What growth phase were cultures in? How many replicates did you use? How do the enrichments change over time, i.e. regarding the percentage of Asgardarchaeota relative to other organisms or are these pure enrichments? Where are the inocula coming from: e.g. do you have a master enrichment, do cultures continuously evolve, or do you have cryostocks, etc? If the latter, can you provide a protocol? How much enrichment was needed for the various analyses. See also some concrete examples below:

We thank the reviewer for this comment. We added more details on the media composition and a better explanation of the passaging of the cultures. There is no master enrichment and we pass

our cultures continuously from flask to flask. Unfortunately, we have not yet been able to establish cryo stocks and cannot share protocols for this yet.

Regarding the reviewer's question about the change of the enrichment composition over time, we present a representative growth curve below, measuring the relative abundance (**left**) and the cell number/mL (**right**) of methanogens, Halodesulfovibro and L. ossiferum by qPCR using primers for mcrA, dsrA and Loki 16S rRNA, respectively. The shown trend is consistently observed in our cultures: After the initial inoculation, the enrichment is dominated by Halodesulfovibro and L. ossiferum reaches higher abundances of up to 70% after 2-3 weeks. This trend is reproducibly observed, but the rate at which L. ossiferum takes over in the culture varies.

Currently, we are working on establishing dual enrichments of L. ossiferum with only one additional symbiont, but this is challenging cultivation work.

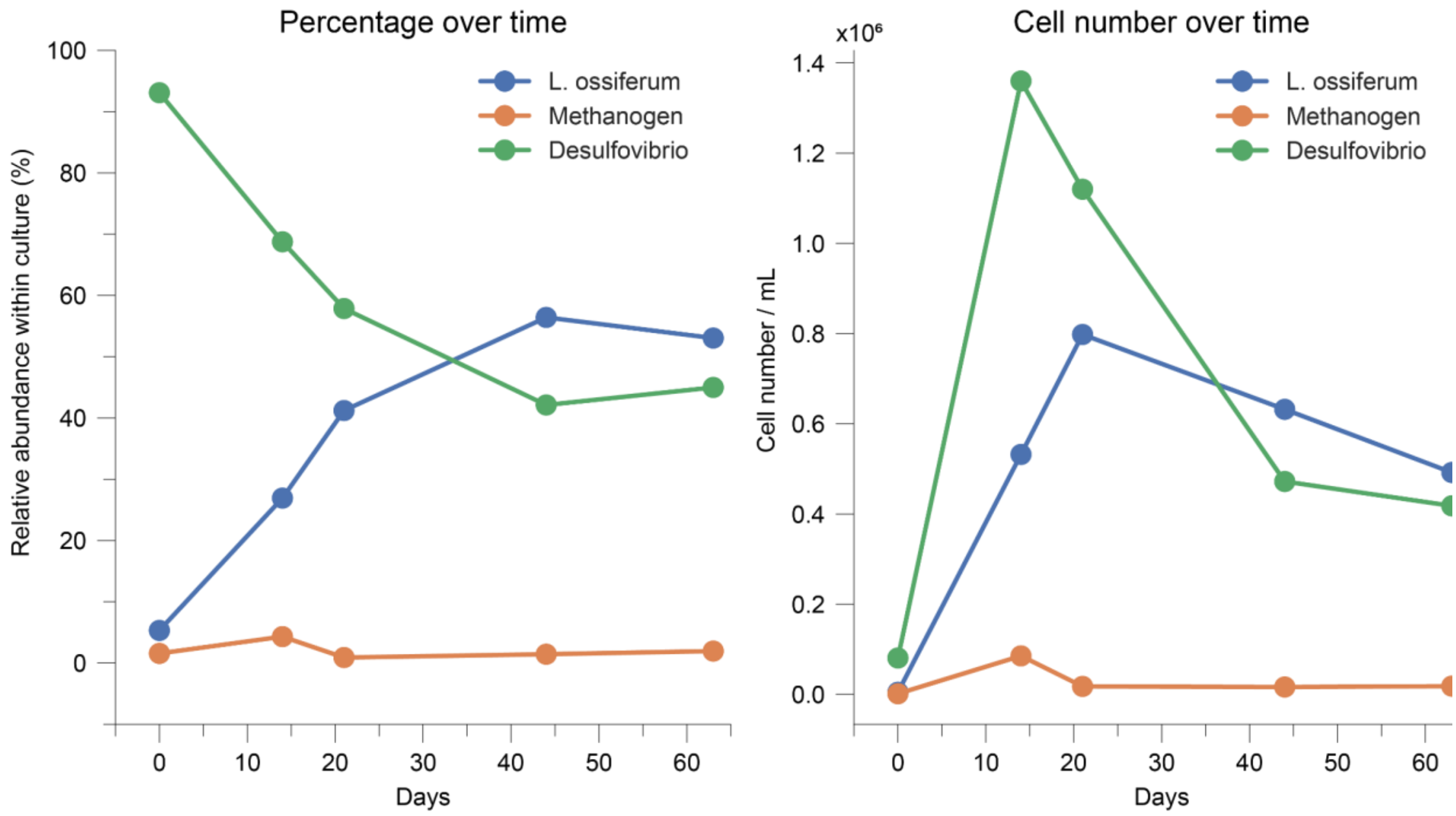

Figure 8: Representative growth curve of a L. ossiferum enrichment culture

Representative growth curves of a single L. ossiferum enrichment culture showing the relative abundance of L. ossiferum and its syntrophs (*left*) as well as their total cell numbers (*right*).

We are now describing the proteomic- and Co-IP analyses and the cultures used in more detail in the Methods section, please find the detailed responses below.

- can you expand a bit on how the actin inhibitors were tested in the methods section "Inhibitor experiments"), including inhibitors and concentrations tested, growth state of culture, replicates etc? Or, what is the difference between these two sections; "Effect of actin inhibitors" and "Inhibitor experiments". Should these sections be merged?

We improved the description of our inhibitor experiments in the methods section and have referred to **Supplementary Table 1**:

"Initial experiments of actin inhibitors were performed at 25, 12.5 or 5 µg/mL final concentrations **(Supplementary Table 1)**. For these experiments we used a single culture (24 days, $1.73*10^7$ L. ossiferum/mL and 58% relative abundance) and tested the effect of seven inhibitors each on three subsequent days, to ensure that inhibitor effects are not affected by changes in the cell

cycle. We used concentrations higher than what is recommended for eukaryotic cells to compensate for potential lower, hampered affinities for Lokiactin. Subsequent titration experiments were performed with inhibitors that had affected the morphology and protrusions of L. ossiferum in the initial screen. For this we performed a serial dilution of the inhibitors (stock solutions either 1 or 2.5 mg/mL) in DMSO to reach concentrations from 250 - 0.25 µg/mL or 100 - 0.1 µg/mL. We then added 1 µL from each inhibitor dilution to 199 µL of L. ossiferum culture in a N_2:CO_2 atmosphere. These titrations were performed with a single culture (25 - 28 days, 4.21*10^6 L. ossiferum/mL, 22.57% rel. abundance) for Chaetoglobosin A and Pseudofuscochalasin A. For Aspochalasin D and Swinholide A, titrations were performed with two L. ossiferum cultures (27 and 35 days old, 3.1 and 4.3*10^6 L. ossiferum/mL, 20 and 22% relative abundance). More details about the used actin inhibitors are provided in **Supplementary Table 1**."

The two different method sections the reviewer is referring to describe either the experimental setup or the image analysis. We renamed the second section to "**Quantifying the effect of actin inhibitors on L. ossiferum morphology and dynamics**" and hope that the reviewer will find this improved.

- regarding the Co-Immunoprecipitation methods section: can you add some information about the growth phase of the culture used for this analysis?

In short, Co-IPs were performed with three large scale cultures in the early stationary phase (2x40 and 1x47 days, 500mL Volume, 1.8 – 3.5 *10^6 L. ossiferum/mL and relative abundances above 20%). Each culture was split into two to perform Co-IP with Lokiactin-antibody-coupled beads and IgG-coupled beads as control.

We have added information about the cultures to the Methods section:

"For Co-IP, we selected three large scale cultures (500 mL each) in stationary phase (1x 47 days and 2x 40 days old) at densities of 1.8 – 3.5*10^6 L. ossiferum/mL and relative abundances above 20%."

- please also add information about the cultures and replicates subjected to Liquid chromatography-mass spectrometry analysis and Mass Spectrometry data analysis

For the proteome analyses, we used three large scale cultures (500mL) in the stationary phase (56 days old; 1.1 – 2.4 *10^6 L. ossiferum/mL and relative abundances above 30%) and split them into 5x50mL (technical replicates). We replotted **Fig. 3k** and **Fig. S5b** to show the mean protein expression level from each biological replicate instead of the technical replicates, as this might be misleading.

We have now added information about the cultures used to the Methods section:

"To quantify expression levels of proteins in L. ossiferum we selected three 500mL (large scale), 56 days old cultures in stationary phase at densities of 1.1 – 2.4*10^6 L. ossiferum/mL and relative abundances above 30%."

- Phylogenetic analyses: how were gelsolin sequences from Asgards selected? Did you test the more complex models of evolution such as C-series model that are not automatically tested by IQtree and perform H-like approximate likelihood? I would recommend adding more gelsolins from all Asgard archaea and assemble a comprehensive dataset for eukaryotic homologs (e.g. by identifying homologs using key protein domains) rather than just adding some of the homologs: specifically, I wonder whether the current phylogenetic analyses could be biased by

LBA due to inclusion of only few long-branching Asgard gelsolins leading to misclassifications of the analysed Asgard gelsolins. It might also be easier to read the tree in supplementary Figure 5 if it was displayed vertically, eukaryotic and asgard homologs were clearly labelled and bootstraps shown. (in that case Fig 5b would not be needed). Please also provide information about the length of the alignment: is it long enough to resolve the relationships?

Regarding the reviewer's concern about sequence sampling and model complexity, we have performed an extensive update to our phylogenetic pipeline. We appreciate the suggestion to use key protein domains for a more comprehensive search; we have now expanded our dataset to include a much broader range of Asgard archaea and eukaryotic supergroups. To address the possibility of long-branch attraction (LBA), we implemented the PMSF approach to approximate the C-series model as recommended, and we further validated our results by reconstructing the tree after removing the top 20% fastest-evolving sites. The consistency of the B35 gelsolin clustering across these rigorous tests confirms that our findings are not biased by limited sampling or model misspecification.

We have also addressed the reviewer's helpful feedback regarding the visualization of our results. Supplementary Figure 6 has been completely redrawn in a vertical orientation, which allows for a clearer presentation of the individual taxa. We have applied distinct color schemes to differentiate between Eukaryotic and Asgard homologs and have added bootstrap support values to all nodes. Finally, the alignment length of 5,821 sites has been documented in the revised manuscript, providing further evidence that the data is sufficiently informative to resolve the relationships in question.

- Supplementary tables and movies:

I could not see the original file names of the supplementary tables and movies so it wasn't entirely clear to me when they were cited in the manuscript: but this might just be due to the conversion of the files in the system. Perhaps references to the tables could be added to the method sections for clarification of some of the missing details. Are there legends to describe the Supplementary Tables somewhere (I could not find them)?

The original file names and captions have been lost in the file conversion. We have therefore added titles and captions for all supplementary tables and videos into the manuscript text now and are referring to the tables more often in the Methods section.

DATA AVAILABILITY

- it would recommend that you make all source and microscopy data available via a repository rather than via contact of the first author. Also, the sequence and alignment files and results from phylogenetic analyses as well as scripts should be made available.

We agree and have now uploaded the source data for the figures, the Python notebooks used to generate the figures together with the microscopy data for the supplementary videos as well as the sequence and alignment files from the phylogenetic analysis here: https://figshare.com/s/eae416d8759ae4228ece?file=59768339.

Referee #4 (Remarks to the Author):

One fundamentally important question in evolutionary cell biology is how did the dynamic cytoskeleton of eukaryotic cells evolve? The acquisition of new modes of motility and contractility enabled cells to successfully hunt prey, divide and form multicellular clusters and tissues, for example. The recent exciting discoveries of archaeal actin along with a handful of actin regulators (e.g. profilin and gelsolin-like proteins) and identification of actin filaments in Archaeal cells raised the question of whether actin dynamics could drive the formation of dynamic protrusion and even motility in these organisms. Radler et al answer this with a resounding **YES**. Live cell imaging of two different species of Archaea (L. ossiferum and M. peptidophilum) reveals a dynamic morphology, with a cell body that can change its shape and actively extend multiple thin, actin-filled protrusions that branch and retract. These can be visualized using the live cell stain FastAct, consistent with previous work identifying actin filaments in Archaeal extensions. A systematic screen of actin inhibitors showed that select cytochalasin derivatives or Swinholide promote loss of protrusions and cell adherence. Interestingly, for both species the rate of protrusion extension matches the speed of the cell body, with the larger M. peptidophilum cells moving faster than the smaller L. ossiferum cells. Inhibition of actin dynamics results in a loss of protrusions, reduced motility and detachment, providing strong support for the conclusion that actin dynamics are critical for the observed motile activity. The analysis presented suggests that the cells move either by gliding or pulling from the tip of the protrusion, but not by pushing from the cell body. Co-IP using a Lokiactin antibody identified several actin regulators, profilins and gelsolin-like proteins, that have been shown in other studies to have polymerizing and severing activities in vitro, in the pellet. Together, these data reveal that Archaea can migrate and strongly suggest that they could do so via dynamic actin polymerization of elongated protrusions controlled by archaeal profilin and gelsolins.

The results here provide an exciting glimpse into the emergence and use of actin dynamics in early evolution. The work establishes methods for live cell imaging of Archaea, along with visualization and manipulation of the actin cytoskeleton, important technical advances that will propel future studies in the field and serve as a template for others investigating the cytoskeleton in evolutionarily divergent organisms. The motility is carefully described and the data showing long, thin protrusions are critical for it is consistent with actin dynamics playing a critical role in this unique mode of migration. The identification of actin regulators that co-precipitate with Lokiactin is interesting, but the work seems to be preliminary in the sense that a list of proteins is presented and the story ends there. This is not surprising given the challenges of working with a divergent cell type, but it leaves one with mostly speculation and not knowing what to make of it all. Thus, as with any significant advance, the findings prompt many questions that go beyond the scope of the present work. These include, but are not limited to, what are the identity and characteristics of the key players and the detailed molecular mechanisms responsible for actin dynamics, which proteins mediate substrate adhesion and how are they linked to actin filaments, how do the protrusions pull the cell body and/or do they mediate gliding, and how are the dynamics of Lokiactin controlled by the candidate regulators in vivo. The findings have set the stage for future work that will require continued development of methods to analyze the proteins and cellular behavior that will surely provide the broader cytoskeletal and evolutionary cell biology fields with more intriguing and surprising discoveries.

We thank the reviewer for the very positive assessment and the excitement about our manuscript. We agree that our findings open up many more questions, which we hope to answer in the future by advancing our imaging setups further. To understand the regulation of Lokiactin polymerization, we are in an active collaboration with groups from ISTA to purify Lokiactin and its regulators to reconstitute the polymer dynamics. To improve our understanding of the role of

adhesions, we aim to adjust substrate topology and composition. However, these projects will need more time and, as the reviewer points out, will be most exciting to follow up in future work.

SPECIFIC COMMENTS

1) The authors test a series of actin drugs and identify several that affect actin filament formation in L. ossiferum (Fig 3D, Supp Table 1). Many will be familiar with cytochalasins, but perhaps not the existence of so many variants, and may have only heard of swinholide A, but not know its mode of action. It would be helpful if the authors could provide a brief description of what is known about how these compounds disrupt actin polymers/polymerization. If the structure of Lokiactin is similar enough to that of crenactin, can one predict if known interaction sites for swinholide A and cytochalasins (presuming their interaction with actin is the same as the widely use cytochalasins A and D) are the same as seen for eukaryotic actin?

We thank the reviewer for bringing up this point, which is very interesting and important. First of all, we did not want to deviate too much from the main results and thus refrained from explaining the mechanisms of the different actin inhibitors in the initial manuscript version. However, we have now added some more information about their modes of action, as follows:

"Most of the tested compounds were cytochalasans, which are thought to bind to the fast-growing barbed ends of actin filaments, thus disrupting their polymerization. Swinholide A is a macrolide toxin, shown to sequester actin dimers and sever eukaryotic actin filaments."

We are now also referring to a recent review elaborating in detail on current knowledge on the precise effects of distinct cytochalasans on actin filaments and associated actin-binding proteins, which is more complicated than commonly thought[9].

In brief, there are no structural studies on the precise binding of any cytochalasan to *bona-fide* actin filaments at present, for instance, using modern cryo-EM techniques, although we also know that highly relevant studies like this are on their way. What is available is a crystal structure of a single cytochalasan, CytoD with a non-polymerizable actin variant (A204E/P243K-mutant in Drosophila 5C actin[10], which does not, however, adopt the "true" structure of barbed ends in polymerized filaments, so information on the binding mode derived from this structure has to be treated with some care. Neither are there any structures from other cytochalasans with eukaryotic actin nor, of course, Lokactin or Crenactin filaments. Another problem is that any structural predictions, for instance using alphafold will currently always be biased towards monomer structures (irrespective of the actin used), with limited affinities likely for respective cytochalasin, so this approach did also not appear too promising. What is available though is information on the proposed residues on eukaryotic actins, so we have performed alignments to compare those interaction surfaces with cytochalasin D in eukaryotic actins with Loki- and Crenactin. The interaction surfaces with Swinholide A were derived from[11].

More specifically, we have blasted the sequences from rabbit Actin, Lokiactin from L. ossiferum and Crenactin from *Pyrobaculum calidifonti*s. The sequence of Crenactin is quite divergent from eukaryotic Actin, with a sequence conservation of only 22% compared to 59% for Lokiactin. The resulting alignment is shown below.

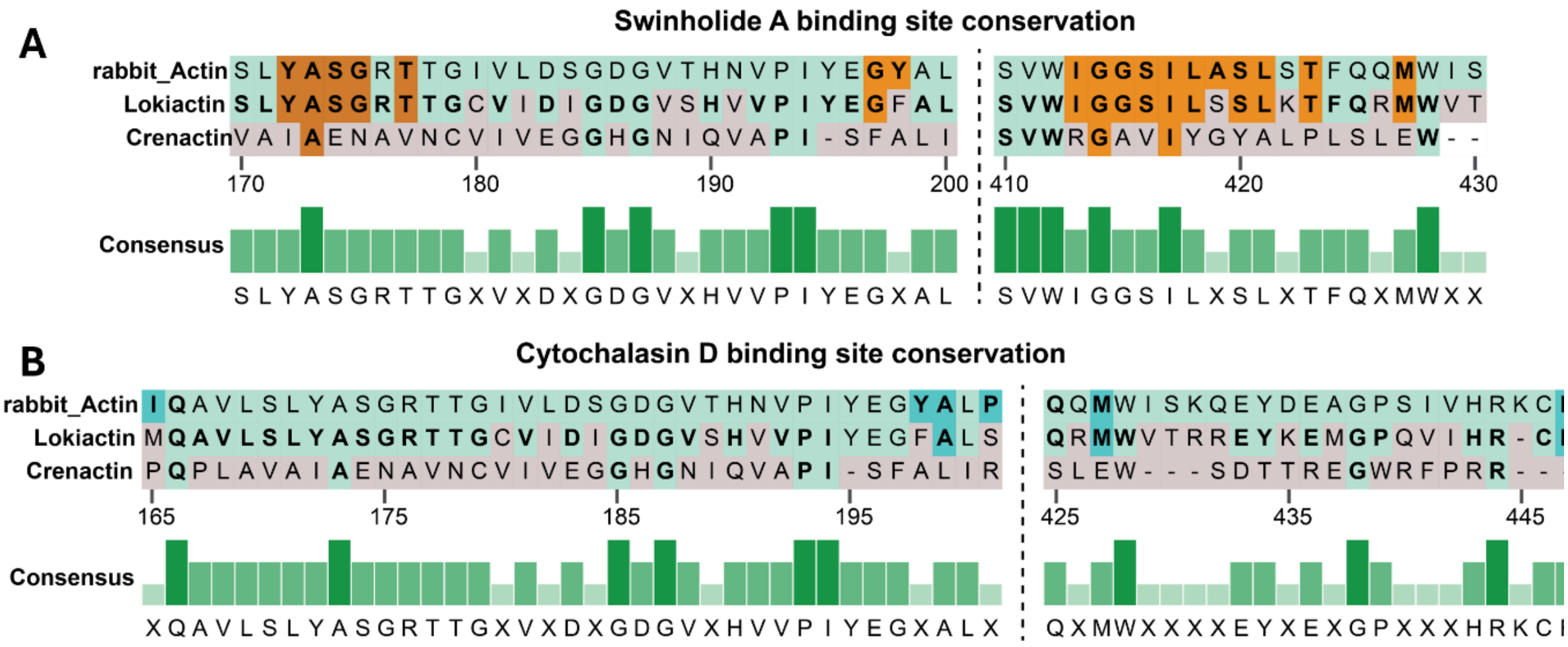

Figure 9: Conservation of Actin inhibitor binding residues in Lokiactin and Crenactin

Alignment of rabbit actin with Lokiactin from L. ossiferum and Crenactin from *P. calidifontis*. Only amino acid stretches important for interaction with Swinholide A (top) and Cytochalasin D (bottom) are shown. Important residues for interaction with SwinA and CytoD are colored orange or cyan respectively. Conserved residues are colored with turquoise and non-conserved residues are colored grey.

It appears that the Cytochalasin D binding surfaces are less conserved than those for Swinholide A binding, which could explain the varying effects for distinct cytochalasans.

However, it should also be pointed out again that these considerations for the latter are solely based on Cytochalasin D, with moderate activity on L. ossiferum protrusions. Together with the notion that cytochalasans constitute a group of broad and chemically highly diverse secondary metabolites, the activities of most of which are not fully characterized[9] our observations on differential inhibitory effects are comprehensible.

2) Analysis of the motility of L. ossiferum shows different modes, including random and sometimes persistent. The authors comment that the persistent movement appears "as if aimed towards a signal". This may be the case, but it is not unusual for migratory cells to have periods of persistent movement so this seems rather speculative. Later, it is stated that M. peptidophilum cells "migrated along the glass randomly, most likely due to the lack of a target." Again, the implication that motility is directed by a signal or target, while tempting, is not supported and without an analysis of the proportion of each type of motility the cells exhibit (both cell types) it is hard to say much about persistent motility, or lack thereof, here. However, if the cells are moving with high persistence towards a signal, then what could it be? Is there any relationship between these persistent motile periods and a neighboring L. ossiferum or even a syntrophic partner?

We thank the reviewer and agree that "a movement towards a potential signal" is rather a bit of an overstatement. We have now removed the statements in question. Observing persistent motility more often in the presence of neighboring cells would be exciting, but we have failed to see this so far. If attraction exists, we have hitherto missed it in our current assays. However, we have not yet been able to include any gradient of potential chemoattractant. As soon as a candidate emerges, experiments of these kinds will be performed. Indeed, future efforts will aim at defining this novel type of motility better, in particular in the context of potential attractants, both chemotactic or haptotactic and in the context of regulating adhesion and directed migration.

3) Potential actin binding proteins are identified by a combination of proteomic analysis and co-immunoprecipitation using anti-Loki antibodies. One concern is that validation of the antibody is

not provided - while the immunostaining strongly supports its specificity, anti-peptide antibody reactivity can be sensitive to context (e.g. works in western blots but not staining and vice versa) and its possible that the antibody could cross-react with other proteins in solution. More details about the validation should be provided.

We have used here the same antibody against Lokiactin that was already raised for a previous publication[12]. The antibody specificity was confirmed in this manuscript by Western Blotting (**Fig. 5f**), immunostaining (**Fig. 5g-h**) as well as immunogold localization (**ED Fig. 10a, b**), all of which suggested specific labeling of actin in protrusions and cell body.

For our Co-IP experiments, we have performed controls with beads coupled to IgG antibodies to only report proteins that are significantly enriched with the Lokiactin antibody. We are now referring to the validation of the antibody in the Methods section as follows:

"The Lokiactin antibody was raised against a Lokiactin-specific peptide (CTFYTDLRVDPSEHPV) and previously validated by ELISA, Western Blot and Immunogold localization."

4) The section describing the proteomics and co-IP (starting on the 2nd paragraph on page 10) is a bit confusing to follow. It starts off with "Our proteomic analysis confirmed the expression of gelsolins and profilins within the top 500 out of 3061 expressed proteins....". At first read it is not clear how the authors arrive at expression levels from a proteomic analysis because none has been described at this point. Was this an RNA analysis or a proteomic analysis that looks at total cell proteins or something else? Please provide a more clear description of what was done here.

As all reviewers pointed out that this section needs improvement, we shortened this section and, following the suggestion by reviewer #1, i.e. moved the results obtained from the Co-IP and proteomic analyses to Figure 3, directly following the Lokiactin inhibition experiments. We have also improved our description of the experimental details in the Methods section.

5) Mov S9 - FastAct treated cells

It appears as if protrusions interact with each other, adhering cells together - is that an accidental/coincidental interaction just seen is this movie or is it observed in other cases where two cells are nearby each other?

We have rarely observed L. ossiferum reaching out to each other. The majority of the cells that are in close proximity do not seem to interact with each other, at least in the time frames of our imaging experiments.

6) FastAct treatment decreases the protrusion extension rate rather significantly, from 1.5 μm/min to 0.7 μm/min (Fig 3b). Is there a corresponding slowing of the cell body speed?

Unfortunately, we don't have a sufficient amount of data with fluorescently labelled L. ossiferum cells to provide a reliable quantification of their migration rates at this stage. Unfortunately, staining L. ossiferum cells with FastAct decreases the overall number of adherent cells, thus further impeding this analysis. But this will be an interesting path for future analysis.

7) Mammalian cells expressing the archaeal gelsolins 2D-Gel and 2D-Gel2 that have been treated with ionomycin to induce global increase of calcium results in a significant reduction in actin polymer (Akil et al, PNAS, 2020; Comm Biol, 2022). While it is possible that ionomycin may not work with archaeal cells, have the authors tried applying it to cells or use any other manipulation that would alter calcium levels, to see if it might affect protrusion dynamics in any way?

We now performed experiments on L. ossiferum cells that were incubated with the calcium ionophore A23187 at increasing concentrations (1-1000 nM) for 1.5 hours.

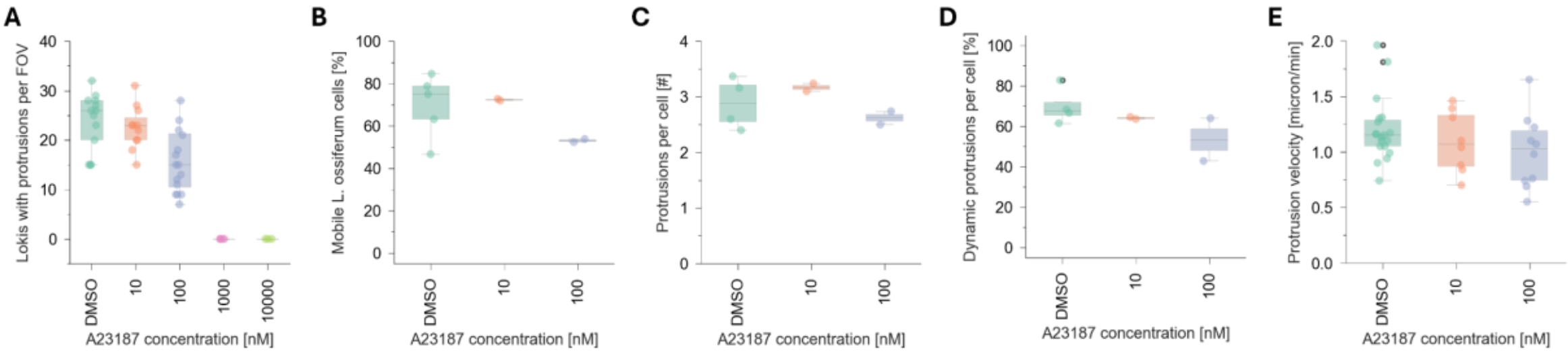

Figure 7: Effect of the calcium ionophore A23187 on L. ossiferum

Effect of increasing A23187 concentration on the density of L. ossiferum per FOV **(A)**, the motility of L. ossiferum **(B)**, the number of protrusions per cell **(C)** as well as the percentage of dynamic protrusion per cell **(D)** and the growth rate of protrusions **(E).**

Indeed, we have failed to find any adherent L. ossiferum cells with protrusions at concentrations above 100nM A23187 (**A**). We found a slight drop in the mobility (**B**) and the percentage of dynamic protrusions per cell (**D**) at 100nM A23187, but no significant change in the number of protrusions (**C**) nor the velocity of the growing protrusions (**E**).

We want to point out that it is unclear if the Ionophore A23187 works the same way for Archaea as it does for eukaryotes and what intercellular processes it affects. While there seems to be a small effect in terms of motility, we cannot be certain whether this is a specific effect of the ionophore. Hence, we decided to not include this dataset into the manuscript.

An additional aspect is, that we might miss immediate effects of the ionophore, as the current method requires incubation before imaging because we can't access cells in the flow chamber through manipulation from outside. In future work, we aim to establish micromanipulation experiments, to study the immediate effect of compounds, such as A23187, on cell morphology and dynamics. We thank the reviewer for this insightful suggestion.

We have removed the sentence referring to $CaCl_2$ from the discussion and refer only to the results from the Robinson group in the results section concerning Figure 3, as we cannot precisely interpret the calcium ionophore on Loki cells and now find that this comment was too speculative.

8) Are actin filaments in protrusions are bundled or are they largely are single filaments based on the available structural information (e.g Rodrigues-Oliveira, Nature, 2022)?

In both, existing and unpublished tomograms by collaborators, we so far find single or double filaments formed by Lokiactin. In their recent preprint, Imachi et al [13] show evidence for bundled Actin filaments in M. peptidophilum cell bodies by cryo EM in **Fig 2l, n.**

MINOR POINTS

a) Fig 1e The imaging chambers illustrated here appear to be sealed at both ends with Vaseline but it appears that maintaining and anoxic atmosphere required flowing in a N2:CO2 mix. It might be useful to illustrate this in the lower diagram.

We thank the reviewer for this comment. We have tried to improve the diagram and have explained our setup better in the Results and Methods section:

Results section

"For both methods, the flow chambers were prepared in anaerobic conditions in a N_2:CO_2 atmosphere and then sealed. PC microscopy was used without environmental control of the atmosphere for short term experiments, lasting at most 30 minutes. For DIC microscopy, the surrounding atmosphere of the flow chamber was kept at 85:15% N_2:CO_2 by constant gas flow into the chamber and experiments were recorded for up to six hours."

Methods section

"DIC experiments were performed with a Nikon Ti2-E microscope, equipped with a 100x Nikon 1.45 PlanApo oil objective. The imaging slide was mounted with a custom-printed 3D slide holder and a custom slide holder stage. Next, a gas chamber was placed on top, enabling environmental control of the atmosphere surrounding the slide. The temperature was kept at 22 °C and the atmosphere of the gas chamber at 15:85% N_2:CO_2 by flowing in the gasses at a flow rate of 13 l/h using an Ibidi Gas Mixer. Oxygen levels were checked with Anaerostrips placed next to the flow chamber. The sealing with Vaseline minimized evaporation of the liquid over the course of the imaging experiment."

b) Fig 1i, third panel The description at the bottom is "Catastrophe" - using the descriptor "Catastrophe" may be confusing as this is typically associated with the rapid disassembly of microtubules

We thank the reviewer for this comment and have renamed "Catastrophe" to "Fast shrinking".

The y-axis for "Shrink" and Catastrophe are labeled "Protrusion growth rate (μm/min)", shouldn't they be retraction rates?

We re-labelled the y axis to "**Protrusion length change rate**". The corresponding x axis adds further information, distinguishing between growing, shrinking and fast shrinking protrusions.

c) The concentrations of the inhibitors tested here are all in ng/mL - while that does convey what was used, it is the standard to use μM.

We now mention the molarity of 50ng/mL SwinA = 36nM in the main text. We would like to keep ng/mL for the x-Axis, as this enables a comparison of the different inhibitors side by side. Otherwise, we end up with different molarities due to differences in their respective molecular weights. Details on the inhibitors can be found in **Supplementary Table 1**.

d) Fig S3E, legend Indicate what NC is (presumably it means negative control). Is each dot an individual cell? Please provide overall N and n values for these experiments (not needed for each separate one, a range should be fine).

The reviewer is correct, NC stands for negative control, which we have now added to the axis. Dots represent individual field of views (FOV) from microscopy, which we used to count L. ossiferum cells/FOV. These initial tests were performed on a single culture to identify suitable compounds for more detailed titration. We have improved our reporting of the repetition of all experiments in the figure legends and the Methods section.

e) Fig S3I Cells treated with swinholide A were stained with FastAct and then the signal quantified and compared with untreated cells. It does appear that the signal is reduced, consistent with loss of actin filaments, but the treated cell body looks to be larger than that of the control. Has cell size been corrected for here?

The reviewer is indeed correct that the cell body of the representative example is bigger than what is shown for the control. We did not correct the measured fluorescent intensities for the differences in cell body size. We have thus adapted our analysis as follows:

We extracted line intensity profiles along the cell body/protrusion from ImageJ. For each profile, background fluorescence was estimated from the first five data points and subtracted from the entire profile, with negative values set to zero. The background-corrected signal was numerically integrated along the cell axis. To account for differences in cell length, the integrated intensity was normalized by the profile length, yielding the mean fluorescence intensity per micrometer for each cell.

This normalization has now changed the precise values but not the overall outcome and trend: we still see a slightly lower FastAct intensity for the SwinA-treated cells, but no statistically significant differences (p-value = 0.05 for the cell body and 0.31 for the protrusion).

We are now showing the results of this analysis in Fig. 3 and report the approach used in the Methods section.

f) The number of replicates done for the co-IP/mass spec analysis is not clearly described anywhere. It appears that at least two or more were carried out and that should be described in the methods or Figure legend.

We have now improved the description of replicates used for co-IP and proteome analyses in the Figure legends and Methods section. We have also changed the box plot presenting our proteomics data (now **Fig. 3k**) to report the average expression level from each of the three cultures used as opposed to the technical replicates.

g) Discussion The authors mention here that red algae are an example of an organism that exhibits motility in spite of its lack of “a more complex migration regulation machinery”. This is an important point, it might also be worth mentioning Trichomonas, although it does have a larger complement of actin regulators (including formins), it lacks myosin yet still appears to able to move in an amoeboid fashion (Kusdian et al, Cell Microbiol, 2013).

We thank the reviewer for pointing this out and have now included the reference in the discussion.

h) Supp Table 2 presents raw proteomic data. The first column in sheet 1 lists protein designators but does not provide protein names. Is there a database where one can look up these proteins or is everything listed in the third sheet? It is not so easy to know what the titles of every column or the numbers mean in some cases (sheet 1, for example). The authors are encouraged to provide a front sheet that explains what is being presented in each sheet in the table.

We thank the reviewer for this comment! We have now added a front sheet explaining the data within Supplementary Table 2 better and tried to improve data presentation for the table as a whole. In Sheet 2 (proteome), we have added another column listing the NCBI accession numbers for each protein. We also make it clearer which data the proteomes represent (technical or biological replicate). We have also moved the protein identifier codes from Supplementary Table 3, to have everything in one place. The raw data of the proteomic data can be found at PRIDE partner repository (https://www.ebi.ac.uk/pride/) using Project accession: PXD074767 and the Token: JtSjtHEEYJuN. The data of the Co-IP experiments is also deposited in the PRIDE repository and can be accessed using Project accession: PXD068820 and Token: Y0KKfVbIjWPZ.

References for Rebuttal

1. Charles-Orszag, A., Wolferen, M. van, Lord, S. J., Albers, S.-V. & Mullins, R. D. Adhesion pilus retraction powers twitching motility in the thermoacidophilic crenarchaeon Sulfolobus acidocaldarius. *Nat. Commun.* **15**, 5051 (2024).
2. Goodson, H. V., Kelley, J. B. & Brawley, S. H. Cytoskeletal diversification across 1 billion years: What red algae can teach us about the cytoskeleton, and vice versa. *BioEssays* **43**, e2000278 (2021).
3. Kusdian, G., Woehle, C., Martin, W. F. & Gould, S. B. The actin- based machinery of Trichomonas vaginalis mediates flagellate- amoeboid transition and migration across host tissue. *Cell. Microbiol.* **15**, 1707–1721 (2013).
4. Merino, F. *et al.* Structural transitions of F-actin upon ATP hydrolysis at near-atomic resolution revealed by cryo-EM. *Nat. Struct. Mol. Biol.* **25**, 528–537 (2018).
5. Pospich, S., Merino, F. & Raunser, S. Structural Effects and Functional Implications of Phalloidin and Jasplakinolide Binding to Actin Filaments. *Structure* **28**, 437-449.e5 (2020).
6. Gerasimaitė, R. *et al.* Efflux pump insensitive rhodamine–jasplakinolide conjugates for G- and F-actin imaging in living cells. *Org. Biomol. Chem.* **18**, 2929–2937 (2020).
7. Eme, L. *et al.* Inference and reconstruction of the heimdallarchaeial ancestry of eukaryotes. *Nature* **618**, 992–999 (2023).
8. Kay, C. J. *et al.* Dated gene duplications elucidate the evolutionary assembly of eukaryotes. *Nature* 1–12 (2025) doi:10.1038/s41586-025-09808-z.
9. Lambert, C. *et al.* Cytochalasans and Their Impact on Actin Filament Remodeling. *Biomolecules* **13**, 1247 (2023).
10. Nair, U. B. *et al.* Crystal Structures of Monomeric Actin Bound to Cytochalasin D. *J. Mol. Biol.* **384**, 848–864 (2008).
11. Klenchin, V. A., King, R., Tanaka, J., Marriott, G. & Rayment, I. Structural Basis of Swinholide A Binding to Actin. *Chem. Biol.* **12**, 287–291 (2005).
12. Rodrigues-Oliveira, T. *et al.* Actin cytoskeleton and complex cell architecture in an Asgard archaeon. *Nature* **613**, 332–339 (2023).
13. Imachi, H. *et al.* Eukaryotes' closest relatives are internally simple syntrophic archaea. *bioRxiv* 2025.02.26.640444 (2025) doi:10.1101/2025.02.26.640444.

Referee #1 (Remarks to the Author):

The authors addressed our concerns carefully and improved the manuscript significantly. The revised version is clearer and overall much stronger than the original submission. We appreciate the thorough efforts made by the authors to revise the manuscript and respond to the comments. This paper will be undoubtedly be highly cited.

We thank the reviewer for their comments, which improved the paper significantly. We also thank them for their kind words and hope their prediction holds true.

Referee #2 (Remarks to the Author):

I co-reviewed this manuscript with one of the reviewers who provided the listed reports.

Referee #3 (Remarks to the Author):

I want to thank the authors for addressing most of my comments as well as those of the other reviewers. I also like the decision of the authors to move parts to the supplement as this has greatly improved clarity of the results. I have just a few remaining small comments/concerns:

- regarding the text on "Gelsolin domain alignment and phylogenetic tree". What do you mean by this: "integrated framework for comprehensive sequence acquisition and stringent data curation?". Isn't this just the canonical/standard approach for building a phylogeny? For gelsolins, it can be helpful to cross-validate whether key interpro domains are present. While you mention the expansion "through a domain-based search across Asgard archaea and Eukaryota datasets", it is not explained how this was done.

We sincerely thank the reviewer for this feedback. We agree that our original phrasing ("integrated framework for comprehensive sequence acquisition and stringent data curation") was overstated for what is essentially a canonical phylogenetic pipeline. We also appreciate the reviewer's suggestion regarding the cross-validation of key InterPro domains. We have now changed the phylogenetic approach, following the reviewers second comment and will reply to this below.

- Regarding the phylogeny more specifically: I appreciate that the authors extended their phylogenetic analyses of gelsolin family proteins and used models of evolution that may be more realistic - though I don't think the authors did model testing to identify the model most suitable to the data and confirm (C60 may be overfitting for single gene trees). Specifically, my concern with this phylogeny was/is that gelsolin homologs in Asgards are generally rather short (1-2 domains) such that there may not be sufficient

information to reliably reconstruct the evolutionary relationship of Asgard and eukaryotic gelsolin homologs (and correctly align with eukaryotic sequences that have more than 2 domains). E.g. which part/domain of the various Asgard homologs (e.g. as illustrated in Figure S5 I) was actually aligned across all the sequences? (E.g. it is possible that the evolution of these genes involved duplications of gelsolins domains within taxa further complicating phylogenetic reconstructions)

We want to apologize to the reviewer as it seems we misunderstood their previous comments: After looking carefully through our alignment and result files, we agree that performing a phylogenetic analysis based on the full gelsolin sequence is limited by the varying protein lengths and the divergent sequences. We want to emphasize that we never aimed to describe a potential relationship between eukaryotic and Asgard gelsolin proteins but were focussing on clustering of Loki B35 gelsolins with previously characterized gelsolins from other Promethearchaeota. Still, we see our error and have redone the phylogeny of gelsolins using a domain and structural-based approach.

To address the issue of varying sequence lengths and repeated domains, we first extracted the individual core domains for all gelsolin sequences. We then used the core gelsolin G1 domain to construct structure-based phylogenetic trees utilizing two independent approaches: the FoldTree[1] pipeline and the recently developed 3Di maximum-likelihood method[2].

Next, to visualize the relationships without relying on complex maximum-likelihood (ML) substitution models, we generated a Sequence Similarity Network (SSN) for the extracted domains. The SSN for the extracted G1 domains was generated using the Enzyme Function Initiative - Enzyme Similarity Tool (EFI-EST)[3,4]. The network was constructed using a minimum sequence length of 80 and a maximum length of 155. To ensure only robust connections were visualized, an E-value filter of 15 (representing an E-value threshold of 10-15) was applied. This threshold indicates the statistical significance of the sequence alignments, and edges were only drawn between nodes (sequences) that shared structural homology fulfilling this threshold. Singletons (nodes without connecting edges at this threshold) were excluded from the final network visualization with Cytoscape.

Furthermore, to provide a robust sequence-based validation,the extracted, unaligned G1 sequences were subjected to the MAFFT-DASH server with “DASH” option to guide the multiple sequence alignment[5]. The resulting structure-informed alignment (354 aligned positions) was then used to construct a maximum-likelihood phylogenetic tree using IQ-TREE3[6].

Although the exact phylogenetic branching positions show some minor variations depending on the method, the overall clustering relationships are remarkably stable across all four independent approaches. Most importantly, all methods consistently demonstrate that 2DGel and other gelsolins clearly separate into distinct clusters.

In our manuscript (suppl. material) we now include the tree built with the 3Di maximum-likelihood method and replace the previous sequence-based tree (**ED. Fig. 6**). We prefer the 3Di method as its theoretical framework is better suited for our data. While FoldTree's neighbour joining (NJ) method is fast, it inherits all the limitations of classical NJ, it relies on the true distance between sequences being close to their observed distance (an assumption often violated in realistic AA datasets) and does not account for site rate variation. 3DiPhy, on the other hand, utilizes a full ML framework. By calculating a substitution matrix from a 3Di-derived BLOSUM-style scoring matrix, it fits the empirical structural data much better and accounts for site rate variations that FoldTree misses.

To avoid any compression artifacts of the figures of the other analysis pipelines performed (SSN, FoldTree and MAFFT), we uploaded the results together with ED Figure 6 here:
https://doi.org/10.6084/m9.figshare.32834969. We would like to avoid overloading the supplementary information with these results, as they are not central to the core message of our manuscript. We discuss the limitations of phylogenies of gelsolins in the supplementary information and hope the reviewer finds this improved.

- The authors suggest that the alignment is longer than 5000 AA but it seems that they counted the positions in the raw alignment file, which includes various sequence stretches that occur only in one or a small number of taxa (i.e. unaligned/uninformative sites). Thus the alignment should be counted after trimming (e.g with Trimal and/or BMGE) which extracts aligned and informative sites (prior to phylogenetic analyses).

We apologize for the lack of clarity in our original description. We completely agree that only aligned and informative sites should be counted and used for phylogeny analyses. We would like to clarify that the length of >5,000 columns reported in the manuscript was, in fact, the final length after trimming with TrimAl. The initial raw alignment was significantly longer, consisting of 11, 643 positions. We realize that because our final trimmed alignment is still quite long, it was entirely reasonable for the reviewer to assume this was the raw alignment. As we have now redone our analysis and perform a structure-based phylogeny using the core G1 domain, this line

was removed from the manuscript and the whole method section shortened significantly.

- A short alignment does not mean the phylogeny is useless, but its worth to caution that the number of informative sites is limited and inferring a reliable relationship of Asgard and eukaryotic homologs is therefore challenging and potentially impacted by artifacts.

We agree with the reviewer and have adapted our method and phrasing as described in an above response to the reviewer.

- Filtering of sites for such short alignments may further decrease information. It is reassuring to read that the "topology remained remarkably stable across different modeling frameworks", though it was not possible to verify this as only one tree was presented. Please note that various clades have extremely low bootstraps indicating that relationships are not well supported (this is expectable with these short domain proteins but should be presented as such).

We thank the reviewer for his critical and constructive feedback and have adapted our method. Additional trees and results from our bioinformatic analysis are attached to this rebuttal.

- Regarding data sharing: it would be helpful, if all alignments (untrimmed, trimmed, filtered) as well as phylogenetic output files were made available in the repository.

We now upload all files and scripts in the figshare referenced in the data availability statement (https://figshare.com/s/eae416d8759ae4228ece?file=59768339).

- Side note: S-Fig. 6 was unreadable in the merged pdf

We are aware of this and apologize. The size of the tree leads to problems with merging the files in Word/pdf. For the final submission, we will provide a high-resolution pdf of Figure ED6. Furthermore, we uploaded the tree together with the results from the additional analysis to figshare for better visualization.

- - legend Figure 4: what is NC?

NC (negative control) experiments are done on cultures without the addition of DMSO. We have added this information to the corresponding figure captions.

- - movie S10: can you clarify what the Swinholide A concentration is for each of the shown images? E.g. I assume it is sorted per column? Perhaps you could label each column for clarity and indicate concentrations.

We now explicit point out the exact concentrations used on top of each movie column and have also added the concentrations to movie **S12**.

- - line 282: I would write "Asgard archaeon" here in line with the usage of Asgard archaea throughout most of your discussion and to avoid confusion because Prometheoarchaeon could also refer to the genus (or any taxonomic rank) of Prometheo rather than to the phylum as a whole. Considering that a Heimdall/Hod lineage is more likely to represent the closest sister lineage of eukaryotes, this could be misleading/ easily misunderstood.

We have replaced Promethearchaeon with Asgard archaeon.

- - line 283: I would write "Such a process may necessitate…" since we currently do not know what the process of taking up a symbiont was.

We have adapted the sentence as suggested.

- - Code availability: will scripts (and/or jupyter notebook files) for data transformation and analysis (proteomics, phylogenetics, plotting etc) be made available as well?

The scripts for plotting the data have been deposited at the figshare. The analysis of the proteomics data was done by the Mass Spec Facility at the Max Perutz Laboratories, part of the Vienna Bio Center. These scripts are available as described in the method section "Data analysis of proteomic samples". The raw proteomic data has been deposited with the dataset identifier PXD074767 as described in the "Data availability" statement. The dataset is available with the token "JtSjtHEEYJuN". The alignment and output files for the phylogeny have also been uploaded to the figshare.

- - line 879: I would rephrase to "While we cannot say whether these…"

We have adapted the sentence as suggested.

Referee #4 (Remarks to the Author):

The work addresses the fundamental question of how eukaryotes evolved the ability to translocate using a dynamic actin cytoskeleton. While it was known that Archaeal cells possess archaeal actin that can form filaments along with a small cohort of actin regulators (e.g. profilin and gelsolin-like proteins), whether these cells could make dynamic protrusions and move on surfaces in an actin-dependent manner as do many eukaryotic cells was unclear. Radler et al reveal the ability of two species of Archaea to make dynamic actin-filled protrusions and move either by gliding or protrusion-mediated pulling of the cell body in an actin-dependent manner. These are

fundamentally important findings that add important new insights in the evolution of cells, in general, and the dynamic actin cytoskeleton, in particular.

The revised manuscript addresses all of this reviewer's comments. It also reads more smoothly, making the story easy to follow, and the data and conclusions are clear and convincing. Notably, the description of the proteomics and the phylogenetic analysis (now in the Supplementary Materials) are much easier to follow and appreciate. Overall, this manuscript presents exciting results that provide important new insights into the evolution of eukaryotic cell migration and will leave readers eager for the next chapter of the story.

We thank the reviewer again for their comments, which improved the paper significantly. We also thank them for their kind words and hope their prediction holds true.

There are a few, small issues remaining that the authors may wish to address -

- lines 206 - 208 (also line 267) - The authors note that an ARF GTPase is relatively enriched in the actin IP pellet, along with a profilin and gelsolins. It is also observed that a BAR domain protein and second profilin are detected, but at levels that are not statistically significant. It would be helpful to readers who may not be intimately familiar with how these proteins are related to the eukaryotic cytoskeleton and migration if the authors were to briefly state why these the ARF GTPase and BAR domain protein may be of particular interest.

To keep the text fluid, we omitted a description of these proteins in the original text, but agree that some information is necessary for readers from a broad audience to appreciate why these proteins are of interest. While it is difficult to compress all proposed functions of Arf GTPases and Bar domains , we added the following sentence to briefly describe their role in the context of coupling membrane deformations with Actin cytoskeleton dynamics:

"ARF GTPases and BAR-domain proteins in eukaryotes can couple membrane remodelling to actin cytoskeleton dynamics, with ARF GTPases regulating phosphoinositide signalling and actin nucleation pathways, and BAR proteins binding to sites of membrane deformations and recruiting actin regulatory factors."

- line 235 - Treatment of M. peptidophilum with SwinholideA results in shorter, but more numerous protrusions as well as '.. deformation of the spherical cell body (Fig S4b-d).' Since the cell body appears as a small dot in those panels it is not easy to appreciate the deformation that the authors describe. In the panels for cells treated with the highest concentration of the drug (Fig S4d) it almost looks as if two or three cell bodies are clustered together. Is this what is being referred to? Is it possible that the drug might be resulting in multinucleation (perhaps that's to speculative at this point)? Regardless, it would be good if the authors describe what they mean by deformation here.

In control experiments HC1 cells dynamically rearrange their cell body constantly, similar as observed with L. ossiferum. Swinholide A (i) arrests this shapeshifting and (ii) leads to a collapse of spherical cell bodies. The multinucleation effect the reviewer is referring to is not a result from the drug, as we occasionally observe HC1 cells with two cell bodies (compare **Movie S11**). We are not yet sure what is the underlying cause and did not describe this observation in more detail, as this will be subject of a future manuscript. We agree with the reviewer that our phrasing was imprecise and describe this now as follows:

"Furthermore, Swinholide A blocks dynamic rearrangement of spherical to elongated cell bodies (and vice versa) and seems to generally cause a collapse of spherical cell bodies, quantified as percentages of disturbed cell bodies (Fig. S4b-d)."

- line 275 - When listing known classes of regulators of complex migration the authors simply say "myosin". Given that different myosins can contribute to cellular motility it would be best to use 'myosins' here.

We thank the reviewer for catching this and have adapted the sentence as suggested.

- Fig 1h - The pink and red lines are not easy to distinguish from each other (the colors look quite similar to each other).

We have recoloured the red line and corresponding arrows to orange.

- Supp Fig5 b - This panel is so small and rather faint, making it hard to see the plot itself and what is being shown. While reorganizing so many panels may be painful, it might be worth trying to find a way to make this interesting set of data much easier to see.

We have adapted the figure panel (colouring and size) to improve the presentation of the data.

- Legend, Supp Fig 5c - It describes showing 'disordered domains' when none are apparent in the panels (but are seen in the model for B35_GelX presented in panel g.

We thank the reviewer for catching this and have adapted the figure caption.

- Supp Fig5 f - The orange box is close enough in color to the pink shaded amino acids that it is a bit hard to see.

We have recoloured the shaded amino acids (now mint green) and chosen a thicker line width for the box to improve visibility.

References

1. Moi, D. *et al.* Structural phylogenetics unravels the evolutionary diversification of communication systems in gram-positive bacteria and their viruses. *Nat. Struct. Mol. Biol.* **32**, 2492–2502 (2025).
2. Garg, S. G. & Hochberg, G. K. A. A General Substitution Matrix for Structural Phylogenetics. *Mol. Biol. Evol.* **42**, msaf124 (2025).
3. Oberg, N., Zallot, R. & Gerlt, J. A. EFI-EST, EFI-GNT, and EFI-CGFP: Enzyme Function Initiative (EFI) Web Resource for Genomic Enzymology Tools. *J. Mol. Biol.* **435**, 168018 (2023).
4. Zallot, R., Oberg, N. & Gerlt, J. A. The EFI Web Resource for Genomic Enzymology Tools: Leveraging Protein, Genome, and Metagenome Databases to Discover Novel Enzymes and Metabolic Pathways. *Biochemistry* **58**, 4169–4182 (2019).
5. Rozewicki, J., Li, S., Amada, K. M., Standley, D. M. & Katoh, K. MAFFT-DASH: integrated protein sequence and structural alignment. *Nucleic Acids Res.* **47**, W5–W10 (2019).
6. Wong, T. K. F. *et al.* IQ - TREE 3: Phylogenomic Inference Software using Complex Evolutionary Models. *EcoEvoRxiv* (2025) doi:10.32942/x2p62n.

Referees' comments:

Referee #3 (Remarks to the Author):

I thank the authors for addressing my final concerns and want to congratulate them for this impressive work.

We thank the reviewer for their critical and helpful comments which signficantly improved this manuscript.

I have two small comments/questions but do not need to see the manuscript again:

- did you update the reporting summary yet to include the new software used for the gelsolin analyses?

  Yes, we updated the reporting summary accordingly.

- can you cite explicitly which figures show these new analyses in the main text?
  - A more detailed description of the identified proteins and a phylogenetic comparison with previously biochemically characterized gelsolins and profilins is found in the supplementary information."
- E.g. is it Ext Data 6 or 3k?

  We now reference Ext. Data 5 and 6 in the main text, which contain the detailed description of the identified gelsolins and profilins.

Referee #4 (Remarks to the Author):

The authors have addressed all of the issues raised in the previous review. There are two, quite minor issues remaining that should be simple to fix.

We thank the reviewer for all their critical and helpful comments that improved this manuscript.

Figure captions:

- In the legend for Figure 1g, h (Line 471 - 473) says that the arrows indicating fast shrinking protrusions are red, while they appear to be orange in the image (lower row, right hand set of panels) and the plot for the corresponding rate of length change is shaded orange.
  We corrected the caption and refer now to an orange arrow.

Supplementary Information file:

- Lines 43-58 The authors describe their initial analysis of putative homologues of PIP lipid synthesis enzymes in the L. ossiferum genome. It would be useful to provide an identifier or accession number for the three enzymes so curious readers can easily find them.
  We refer now to Supplementary table 2, which contains all protein identifiers.

- Line 58 This line finishes the discussion of PIP lipids and is then abruptly followed by “The second type of actin binding protein commonly...” that moves into a discussion of gelsolins. The authors should make this the beginning of a separate paragraph.
  We have adjusted the text accordingly.
