# Supplementary material for: Dynamic protrusions mediate crawling motility in Asgard archaea
Source: Nature. 2026 Sep 30;658(8138):1218–25. doi: 10.1038/s41586-026-11063-9 (PMC13716822; doi:10.1038/s41586-026-11063-9)
Supplement: Supplementary file 2 [file 41586_2026_11063_MOESM1_ESM.pdf]

nature portfolio

https://doi.org/10.1038/s41586-026-11063-9

# Supplementary information

# Dynamic protrusions mediate crawling motility in Asgard archaea

In the format provided by the
authors and unedited

# Supplementary Information of manuscript "Dynamic protrusions mediate crawling motility in Asgard Archaea"

Philipp Radler[1], Tobias Viehboeck[1], Zhen-Hao Luo[1], Nevena Maslać[1], Katharina Schmidt[2], Robert Hauschild[3], Masaru Nobu[4], Silvia Bulgheresi[1], Theresia E.B. Stradal[2], Klemens Rottner[5], Hiroyuki Imachi[4], Michael Sixt[3], Christa Schleper*[1]

[1]Department of Functional and Evolutionary Ecology, Archaea Biology and Ecogenomics Unit, University of Vienna, Austria
[2]Department of Cell Biology, Helmholtz Centre for Infection Research, Braunschweig, Germany
[3]Institute of Science and Technology Austria, Klosterneuburg, Austria
[4]Institute for Extra-cutting-edge Science and Technology Avant-garde Research (X-star), Japan Agency for Marine-Earth Science and Technology (JAMSTEC), Yokosuka, Japan
[5]Division of Molecular Cell Biology, Technische Universität Braunschweig, Germany; Molecular Cell Biology Group, Helmholtz Centre for Infection Research, Braunschweig, Germany
*Corresponding author: christa.schleper@univie.ac.at

## Table of content

Supplementary Discussion: Co-IP, proteome and phylogenetic analyses ..... 2
Proteins and lipid interacting with actin filaments ..... 2
Phylogenetic analysis of gelsolin proteins in L. ossiferum ..... 3
Supplementary references ..... 4
Supplementary Figures ..... 5

# Supplementary Discussion: Co-IP, proteome and phylogenetic analyses

## Proteins and lipid interacting with actin filaments

To predict potential functions of proteins identified in Co-IP experiments potentially perform within L. ossiferum, we compared profilins and gelsolins encoded in the L. ossiferum genome with those previously characterized from other Asgard archaea[1–3].

The single profilin of L. ossiferum, which is highly expressed and found to be enriched in Co-IP experiments (B35_Pro2) shares high structural similarity with a profilin from Odinarchaeota (**Supplementary Figure 1a**, $RMSD_{pruned}$ = 0.896). This profilin was shown to slow actin polymerization in *in vitro* assays and its effect was reversible in the presence of phosphatidylinositol phosphate (PIP) lipids[1]. PIP lipids are commonly found in signalling pathways of cytoskeletal organization in eukaryotic cells, often by regulating binding of actin binding proteins, such as profilins[4]. It is still a topic of debate if bona fide phosphatidylinositol phosphate lipids originated from bacteria or from Archaea[5], but it is generally accepted that PIP-like headgroups are found in all domains of life[6].

To assess whether PIP signalling could mediate Lokiactin dynamics, we performed a structural homology search of the L. ossiferum genome and looked for putative homologues of enzymes involved in PIP lipid synthesis. By doing this, we identified three enzymes that could potentially be involved in PIP signalling: Inositol-3-phosphate synthase (MIPS), which converts D-glucose 6-phosphate to 1D-*myo*-inositol 3-phosphate. Phosphatidylinositol-phosphate synthase (PIPS), which catalyses the conjugation of the 1'-hydroxyl group of 1D-*myo*-inositol 3-phosphate with a lipid tail of cytidine diphosphate diacylglycerol (CDP-DAG), forming phosphatidylinositol phosphate (PIP), and a class I Phosphoinositide 3-kinase (PI3K), which usually catalyzes the conversion of phosphatidylinositol (4,5)-bisphosphate ($PI(4,5)P_2$) into phosphatidylinositol (3,4,5)-trisphosphate ($PI(3,4,5)P_3$) *in vivo*. It has been shown *in vitro* to convert phosphatidylinositol (PI) into phosphatidylinositol 3-phosphate (PI3P). The protein identifiers can be found in **Supplementary Table 2**.

While we cannot say whether these enzymes perform the annotated functions, all candidates were stably expressed in L. ossiferum in all three cultures (**Supplementary Figure 1b**), indicating at least the possibility for PIP dependent cytoskeletal reorganization within Asgard Archaea. Ultimately, lipidomics applied to denser, not yet available Asgard cultures will be required to confirm the presence of PIP lipids.

The second type of actin binding protein commonly found in Asgard Archaea are gelsolins: These differ from eukaryotic gelsolins by having only one to two gelsolin homology (GH)

domains, compared to eukaryotic gelsolins with 6 GH domains[7]. Even though the Asgard gelsolins are missing some of these domains, several gelsolin homologues from Thor, Odin, Heimdall and Lokiarchaea were biochemically characterized previously and shown to affect actin filaments with different capabilities to sever, cap, nucleate and bundle them in *in vitro* assays[2,3].

# Phylogenetic analysis of gelsolin proteins in L. ossiferum

To see if the five gelsolin homologues from L. ossiferum enriched in Co-IP experiments cluster together with previously characterized homologues from other Promethearchaeota we performed a phylogenetic analysis (**Supplementary Figure 2**). Compared to eukaryotic gelsolins, their Asgard homologs are shorter in length, contain less GH domains and their sequences are highly divergent, which significantly hampers sequence based phylogenetic approaches as previously reported[2]. To circumvent these issues, we extracted the G1 core gelsolin domains and performed a structural-based phylogeny based using the recently reported 3DiPhy approach[8] (**Supplementary Figure 2**).

The two gelsolin candidates, containing two GH domains (B35_2DGel1 and B35_2DGel2, **Supplementary Figure 1d**), formed a sister group to other 2D gelsolins characterized previously. These gelsolins were able to either sever preformed actin filaments or to significantly bundle actin filaments. Furthermore, the effect of some 2D gelsolins was sensitive to the presence of calcium, indicating intricate regulation of these gelsolins[2,3]. This finding was linked to the presence of WH2-like motifs, which play a key role in nucleating actin filaments and were shown to be sensitive to calcium signalling in their actin assembly activity[3]. We confirmed the presence of this motif (LKDV) between the two GH domains of both B35_2DGel proteins (**Supplementary Figure 1e, f**).

The GH domain of the three remaining gelsolins localized in another cluster with several other Promethearchaeota gelsolins (**Fig. ED 6**). We note that one of the enriched gelsolins (B35_GelX) shares similar domains with Thor_GelX, previously shown to disassemble preformed actin filaments[2] (**Supplementary Figure 1i)**. B35_GelX contains a single GH domain and a disordered domain ("X") of unknown function and another disordered domain at the C-terminus (**Supplementary Figure 1g**). The fold of the "X" domain is similar to the domain in Thor_GelX (**Supplementary Figure 1c**), but its function is unclear and requires further biochemical characterization by, for example, deleting these domains and observing the potential effects on actin filaments *in vitro*. The GH domains of the remaining gelsolins identified in Co-IP (B35_Gel1 and B35_Gel2, **Supplementary Figure 1h, i**) fell in the same cluster, but we could not identify clear homology to previous characterized gelsolins.

Altogether these observations support the presence of a dynamic cytoskeleton within L. ossiferum, with Lokiactin assembly being likely tuned by gelsolin and profilin variants. Potentially, calcium signalling may also contribute to the spatial and temporal aspects of Lokiactin dynamics, but demonstration of its role will require further studies.

# Supplementary references


1. Akıl, C. & Robinson, R. C. Genomes of Asgard archaea encode profilins that regulate actin. *Nature* **562**, 439–443 (2018).
2. Akıl, C. *et al.* Insights into the evolution of regulated actin dynamics via characterization of primitive gelsolin/cofilin proteins from Asgard archaea. *Proc. Natl. Acad. Sci.* **117**, 19904–19913 (2020).
3. Akıl, C. *et al.* Structural and biochemical evidence for the emergence of a calcium-regulated actin cytoskeleton prior to eukaryogenesis. *Commun. Biol.* **5**, 890 (2022).
4. Hou, X. *et al.* Phosphoinositide signaling at the cytoskeleton in the regulation of cell dynamics. *Cell Death Dis.* **16**, 296 (2025).
5. Esposti, M. D. Eukaryotes inherited inositol lipids from bacteria: implications for the models of eukaryogenesis. *FEBS Lett.* **597**, 2484–2496 (2023).
6. Michell, R. H. Inositol lipids: from an archaeal origin to phosphatidylinositol 3,5-bisphosphate faults in human disease. *FEBS J.* **280**, 6281–6294 (2013).
7. Ghoshdastider, U., Popp, D., Burtnick, L. D. & Robinson, R. C. The expanding superfamily of gelsolin homology domain proteins. *Cytoskeleton* **70**, 775–795 (2013).
8. Garg, S. G. & Hochberg, G. K. A. A General Substitution Matrix for Structural Phylogenetics. *Mol. Biol. Evol.* **42**, msaf124 (2025).

# Supplementary Figures

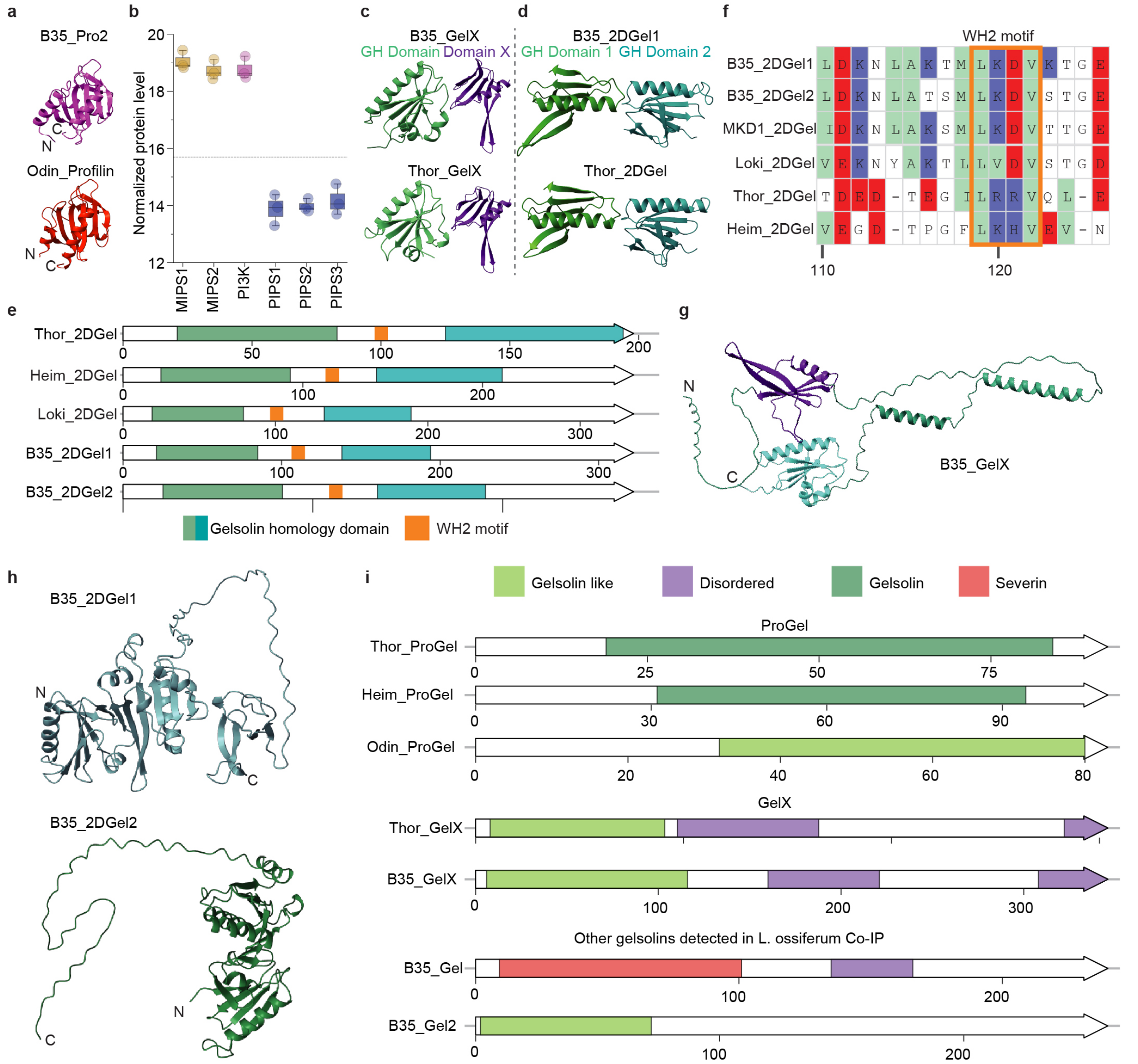


**Supplementary Figure 1: Protein expression, Co-IP and bioinformatic comparisons of gelsolins**

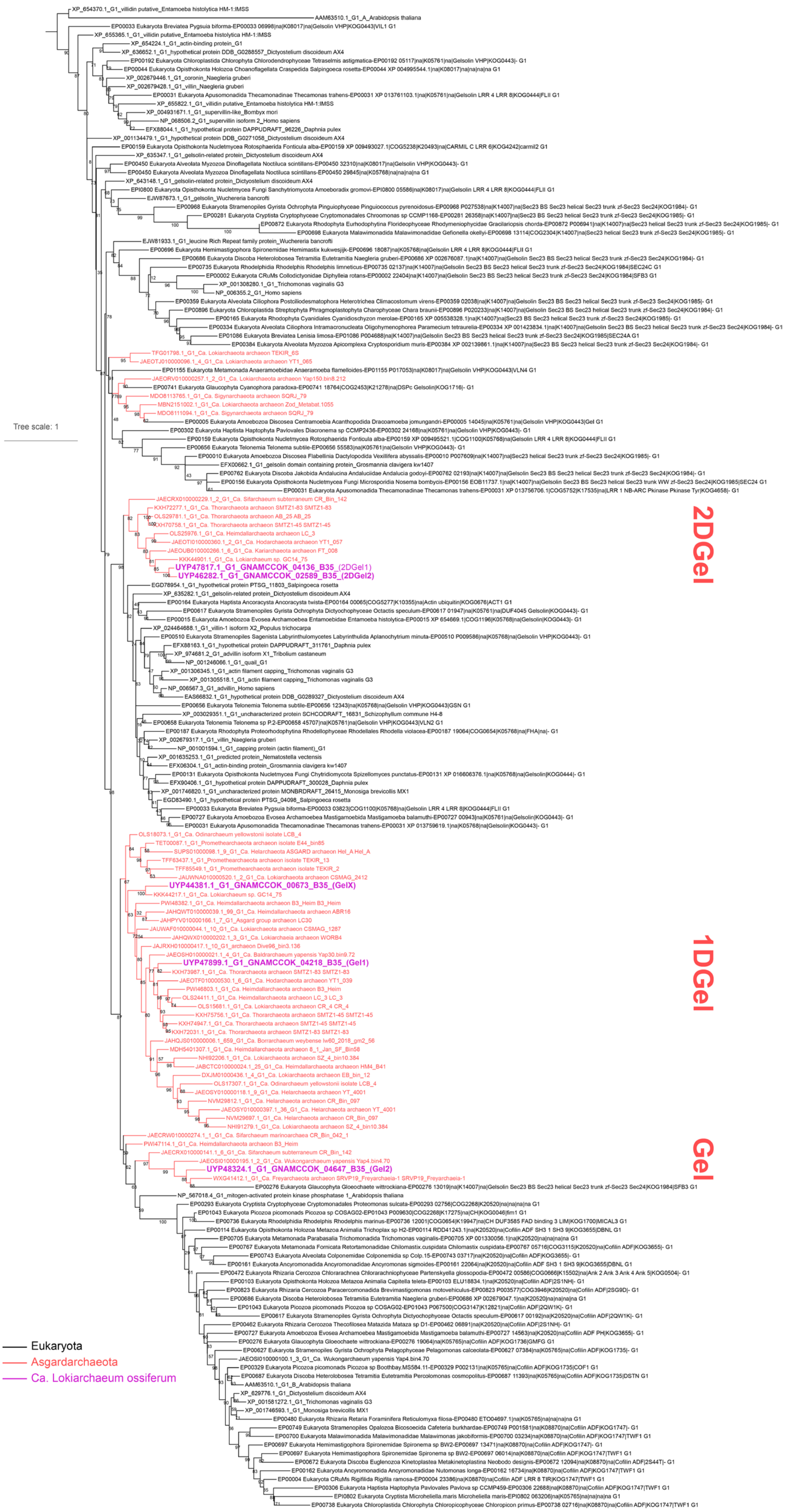


**Supplementary Figure 2: Structure-based phylogenetic tree of gelsolin homologues**
