# Supplementary material for: Dynamic protrusions mediate crawling motility in Asgard archaea
Source: Nature. 2026 Sep 30;658(8138):1218–25. doi: 10.1038/s41586-026-11063-9 (PMC13716822; doi:10.1038/s41586-026-11063-9)
Supplement: Supplementary file 3 [file 41586_2026_11063_MOESM2_ESM.pdf]

nature portfolio

Corresponding author(s): Christa Schleper

Last updated by author(s): Aug 10, 2026

Reporting Summary

Nature Portfolio wishes to improve the reproducibility of the work that we publish. This form provides structure for consistency and transparency in reporting. For further information on Nature Portfolio policies, see our Editorial Policies and the Editorial Policy Checklist.

Statistics

For all statistical analyses, confirm that the following items are present in the figure legend, table legend, main text, or Methods section.

n/a	Confirmed	
☐	☒	The exact sample size (*n*) for each experimental group/condition, given as a discrete number and unit of measurement
☐	☒	A statement on whether measurements were taken from distinct samples or whether the same sample was measured repeatedly
☐	☒	The statistical test(s) used AND whether they are one- or two-sided<br>*Only common tests should be described solely by name; describe more complex techniques in the Methods section.*
☒	☐	A description of all covariates tested
☒	☐	A description of any assumptions or corrections, such as tests of normality and adjustment for multiple comparisons
☐	☒	A full description of the statistical parameters including central tendency (e.g. means) or other basic estimates (e.g. regression coefficient) AND variation (e.g. standard deviation) or associated estimates of uncertainty (e.g. confidence intervals)
☐	☒	For null hypothesis testing, the test statistic (e.g. *F*, *t*, *r*) with confidence intervals, effect sizes, degrees of freedom and *P* value noted<br>*Give P values as exact values whenever suitable.*
☒	☐	For Bayesian analysis, information on the choice of priors and Markov chain Monte Carlo settings
☒	☐	For hierarchical and complex designs, identification of the appropriate level for tests and full reporting of outcomes
☒	☐	Estimates of effect sizes (e.g. Cohen's *d*, Pearson's *r*), indicating how they were calculated

Our web collection on statistics for biologists contains articles on many of the points above.

Software and code

Policy information about availability of computer code

Data collection	No software was used for data collection.
Data analysis	Microscopy data was analysed with FIJI (ImageJ 1.54p), Ilastik 1.4.2 and cell migration was tracked with the TrackMate plugin v 7.6. The data was collected in Excel and csv files and plotted with Jupyter Notebooks. Mass Spec analysis of Co-IP and proteomic experiments were analysed with FragPipe 23.1, MSFragger 4.3, IonQuant 1.11.12 and Philosopher 5.1.12. All these methods are published and accordingly cited in the manuscript. For final analysis the MassSpec facility of the Vienna BioCenter used their developed Pyhton code MsReport 0.0.30 (https://doi.org/10.5281/zenodo.15309090). For the structure based phylogenetic analysis MMseqs2, HT-ColabFold, 3DiPhy and IQ-TREE 3.1.0 were used and appropriately cited.

For manuscripts utilizing custom algorithms or software that are central to the research but not yet described in published literature, software must be made available to editors and reviewers. We strongly encourage code deposition in a community repository (e.g. GitHub). See the Nature Portfolio guidelines for submitting code & software for further information.

Data

Policy information about availability of data

All manuscripts must include a data availability statement. This statement should provide the following information, where applicable:

- Accession codes, unique identifiers, or web links for publicly available datasets
- A description of any restrictions on data availability
- For clinical datasets or third party data, please ensure that the statement adheres to our policy

Plots were created using Jupyter Notebook. Source data for all figures have been provided in data source tables. Microscopy raw data for the supplementary videos and alignments files from the phylogenetic analysis are available from figshare (https://figshare.com/s/eae416d8759ae4228ece?file=59768339). All further microscopy data is available from the first or corresponding author upon request (philipp.radler@univie.ac.at and christa.schleper@univie.ac.at). The mass spectrometry proteomics data have been deposited to the ProteomeXchange Consortium via the PRIDE partner repository with the dataset identifier PXD068820. The mass spectrometry proteomics data of the proteome analysis have been deposited with the dataset identifier PXD074767.

Research involving human participants, their data, or biological material

Policy information about studies with human participants or human data. See also policy information about sex, gender (identity/presentation), and sexual orientation and race, ethnicity and racism.

Reporting on sex and gender	N/A
Reporting on race, ethnicity, or other socially relevant groupings	N/A
Population characteristics	N/A
Recruitment	N/A
Ethics oversight	N/A

Note that full information on the approval of the study protocol must also be provided in the manuscript.

Field-specific reporting

Please select the one below that is the best fit for your research. If you are not sure, read the appropriate sections before making your selection.

☒ Life sciences ☐ Behavioural & social sciences ☐ Ecological, evolutionary & environmental sciences

For a reference copy of the document with all sections, see nature.com/documents/nr-reporting-summary-flat.pdf

Life sciences study design

All studies must disclose on these points even when the disclosure is negative.

Sample size	Sample sizes were not determined by a formal statistical power calculation but were guided by experimental feasibility, throughput and cell density. Microscopy experiments were performed using at least two independent cultures per experiment, and for quantitative analyses of cell dynamics, we aimed to include at least five independent cultures. The enrichment cultures used in this study are not synchronized, limiting precise control over cell-cycle stage. In addition, cell density varies between cultures, which limits standardization of the experimental input. Imaging experiments therefore used cultures ranging from early exponential to early stationary phase, as described in the Methods. For the initial characterization of cell behaviour, the number of independent cultures was considered sufficient to establish the reproducibility of the observed phenotypes.
Data exclusions	Microscopy data was excluded when the focus of the imaging plane was lost or too much background signal disturbed data analysis. For analysis of motility, only cells that showed displacement above 1µm in 10 minutes were analysed.
Replication	More than 20 independent biological replicates (cell cultures of Loki B-35 and HC1) were used across the different analyses presented in this paper. Following initial optimization of the experimental conditions, the dynamic behaviours of Loki-B35 and HC1 could be reproducibly observed across independent cultures. For individual analyses, multiple cultures were assessed for their ability to attach to glass surfaces. Cultures with higher relative abundances and greater numbers of adherent cells were selected for subsequent experiments to facilitate data acquisition. All experiments were performed using at least two independent biological replicates (independent cultures).
Randomization	Randomization was not applicable to this study because it did not involve participant groups or experimental group allocation. Moreover, random selection of cultures was not appropriate for this pilot study because cell abundance and attachment varied substantially between cultures. Given the low overall cell densities, selecting cultures with sufficient Loki-B35 abundance and numbers of adherent cells was necessary to ensure adequate data acquisition and reliable downstream analysis.
Blinding	Blinding was not applicable to this study because the experiments were microscopy-based and required researchers to verify sample identity

Blinding	and experimental controls during data acquisition. In addition, blinded data collection was not feasible because the abundance and density of Loki-B35 cells varied between cultures. For experiments requiring high biomass, such as proteomic analyses and co-immunoprecipitation (Co-IP), cultures with sufficiently high relative abundances and total cell densities had to be selected to obtain adequate material for analysis.

Reporting for specific materials, systems and methods

We require information from authors about some types of materials, experimental systems and methods used in many studies. Here, indicate whether each material, system or method listed is relevant to your study. If you are not sure if a list item applies to your research, read the appropriate section before selecting a response.

Materials & experimental systems

n/a	Involved in the study
☐	☒ Antibodies
☒	☐ Eukaryotic cell lines
☒	☐ Palaeontology and archaeology
☒	☐ Animals and other organisms
☒	☐ Clinical data
☒	☐ Dual use research of concern
☒	☐ Plants

Methods

n/a	Involved in the study
☒	☐ ChIP-seq
☒	☐ Flow cytometry
☒	☐ MRI-based neuroimaging

Antibodies

Antibodies used	Custom antibodies were obtained using the services of Eurogentec (order number 1000997858). The antibody used in this study was produced in rabbits (ab1: CTFYTDLRVDPSEHPV - lot number 2110517) and used at dilutions of 1:500. The secondary antibody used for immunofluorescence was goat anti-rabbit AF488, abcam ab150077 (1:1000 diluted).
Validation	The identity of the peptides was confirmed by LC-MS analysis. The antibodies were validated through ELISA assays using the services of Eurogentec and additionally tested on L. ossiferum cell lysate by Western blotting (see https://doi.org/10.1038/s41586-022-05550-y, Fig. 5f).

Plants

Seed stocks	N/A
Novel plant genotypes	N/A
Authentication	N/A
